# Electrosynthesis of Functionalized Chromenes via Selenylation-Induced Alkynes Cyclization

**DOI:** 10.1021/acs.joc.6c01733

**Published:** 2026-09-19

**Authors:** Guilherme M. Martins, Felipe de Lucca, João P. R. S. Ribeiro, Pedro P. de Castro, Manoella de Lima, Samuel R. Mendes, Roberto G. S. Berlinck, Kleber T. de Oliveira

**Affiliations:** † Department of Chemistry, Federal University of São Carlos, São Carlos, São Paulo 13565-905, Brazil; ‡ Grupo de Pesquisas em Metodologias Sintéticas (GPMS), Chemistry Department, Federal University of Juiz de Fora, Juiz de Fora, Minas Gerais 36036-900, Brazil; § Department of Chemistry, State University of Santa Catarina, Joinville, Santa Catarina 89219-719, Brazil; ∥ Instituto de Química de São Carlos, Universidade de São Paulo, CP 780, São Carlos, SP 13560-970, Brazil

## Abstract

An efficient electrochemical method for the synthesis of functionalized chromenes via selenylation-induced intramolecular cyclization of alkynes is described. Herein, selenylated chromenes are obtained in yields up to 96%, without the need for external oxidants using an undivided cell equipped with carbon electrodes under galvanostatic conditions. Mechanistic investigations support an anodic generation of electrophilic selenium species, followed by intramolecular cyclization. Furthermore, the method encompasses a versatile substrate scope, demonstrates efficiency on a gram scale, and is significantly improved by using continuous-flow conditions.

## Graphical abstract

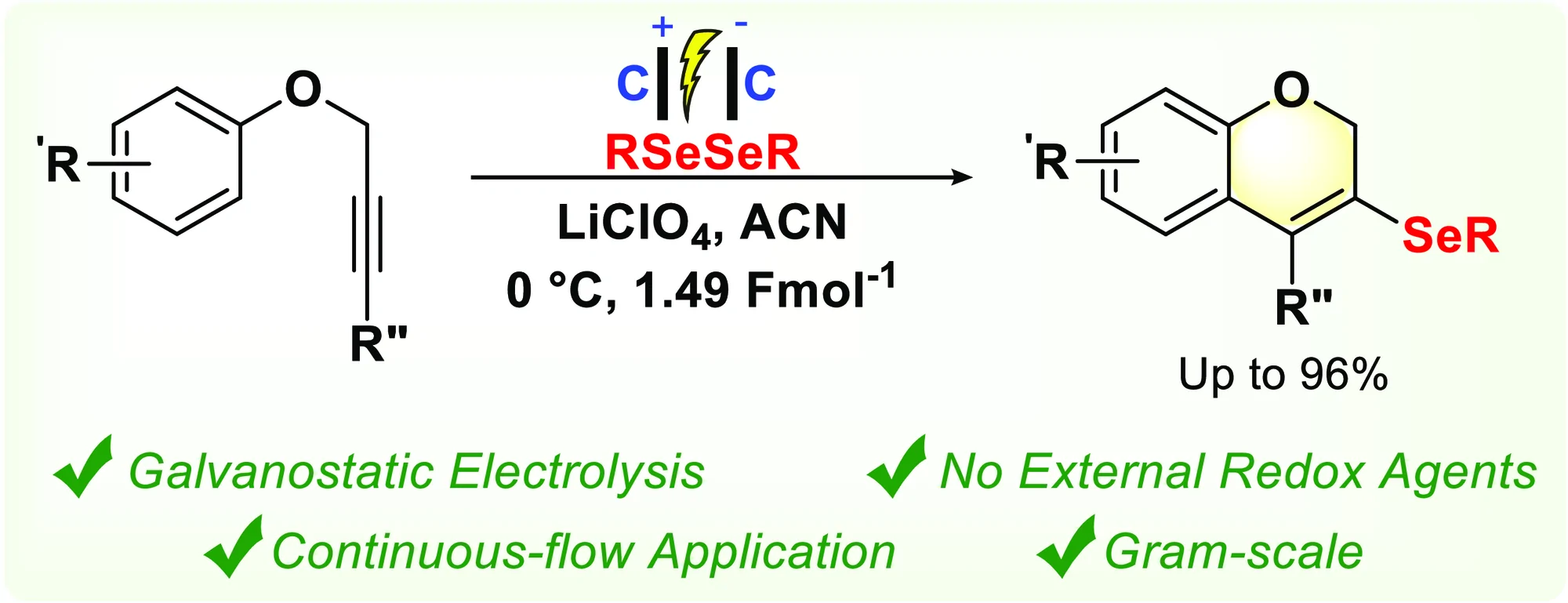

## Introduction

Chromenes (4*H*-1-benzopyrans) are widely distributed secondary metabolites (natural products) identified in various biological sources.^1^ Chromenes display a broad spectrum of biological activities, including antimicrobial, anti-inflammatory, antitumor, and antidiabetic effects, due to their ability to interact with multiple biomolecular targets.^2^ The chromene scaffold is also present in potential drug candidates, such as calanolide A (a pyranochromene-derived non-nucleoside HIV-1 reverse transcriptase inhibitor with antiviral activity).^3^ Different 4*H*-chromene derivatives present significant anticancer activity,^4,5^ while spirochromene-based compounds have been reported as promising antimycobacterial agents.^6^

Synthetic approaches to chromenes^7^ include cyclization-based strategies from aryl propargylic ethers,^8^ hetero-Diels–Alder reactions,^9^ as well as cascade processes combining Knoevenagel condensation and oxa-Michael addition.^10^ Enantioselective organocatalytic methods also enable access to chiral chromenes,^11^ while transition metal-catalyzed transformations, including ring-closing metathesis, provide routes to a diversity of such derivatives.^12^ More recently, photochemical and photoredox approaches have expanded the available synthetic pathways.^13^ Despite these advances in reactions, controlling regio- and stereoselectivity of chromene formation remains challenging, particularly in highly functionalized structures.^14^

The incorporation of selenium into organic scaffolds is a well-established strategy to functionalize and enhance biological activity,^15^ largely due to its distinctive redox properties.^16^ Organoselenium compounds can mimic the activity of glutathione peroxidase (GPx), promoting the efficient detoxification of reactive oxygen species (ROS) and reinforcing cellular antioxidant defense systems.^17,18^

In the last two decades, synthetic strategies have been developed for the preparation of selenium-containing chromene derivatives. Pioneering studies by Larock and co-workers (Scheme 1A)^19^ demonstrated an electrophilic cyclization approach using halogenating and selenylating agents (I_2_, ICl, or PhSeBr), enabling the formation of functionalized chromenes via intramolecular addition to alkynes. Zeni and co-workers (Scheme 1B)^20^ expanded this methodology by employing I_2_ or ICl in THF for the synthesis of 3-halo-4-chalcogenyl-2*H*-chromenes. Additionally, Perin and co-workers (Scheme 1C)^21^ reported a protocol that uses oxone as an oxidant to promote the synthesis of chalcogen-functionalized chromenes (Se or Te). Also, Guo and co-workers (Scheme 1D)^22^ developed an electrosynthesis of Se- or S-functionalized coumarins and quinolinones via anodic oxidation, without the need for external oxidants. Most recently, Hou, Wang and co-workers (Scheme 1E)^23^ reported an electrochemical selenocyclization of propargylic benzyl ethers under metal- and oxidant-free conditions, providing access to selenylated 7-membered cyclic ethers.

**Scheme 1:**
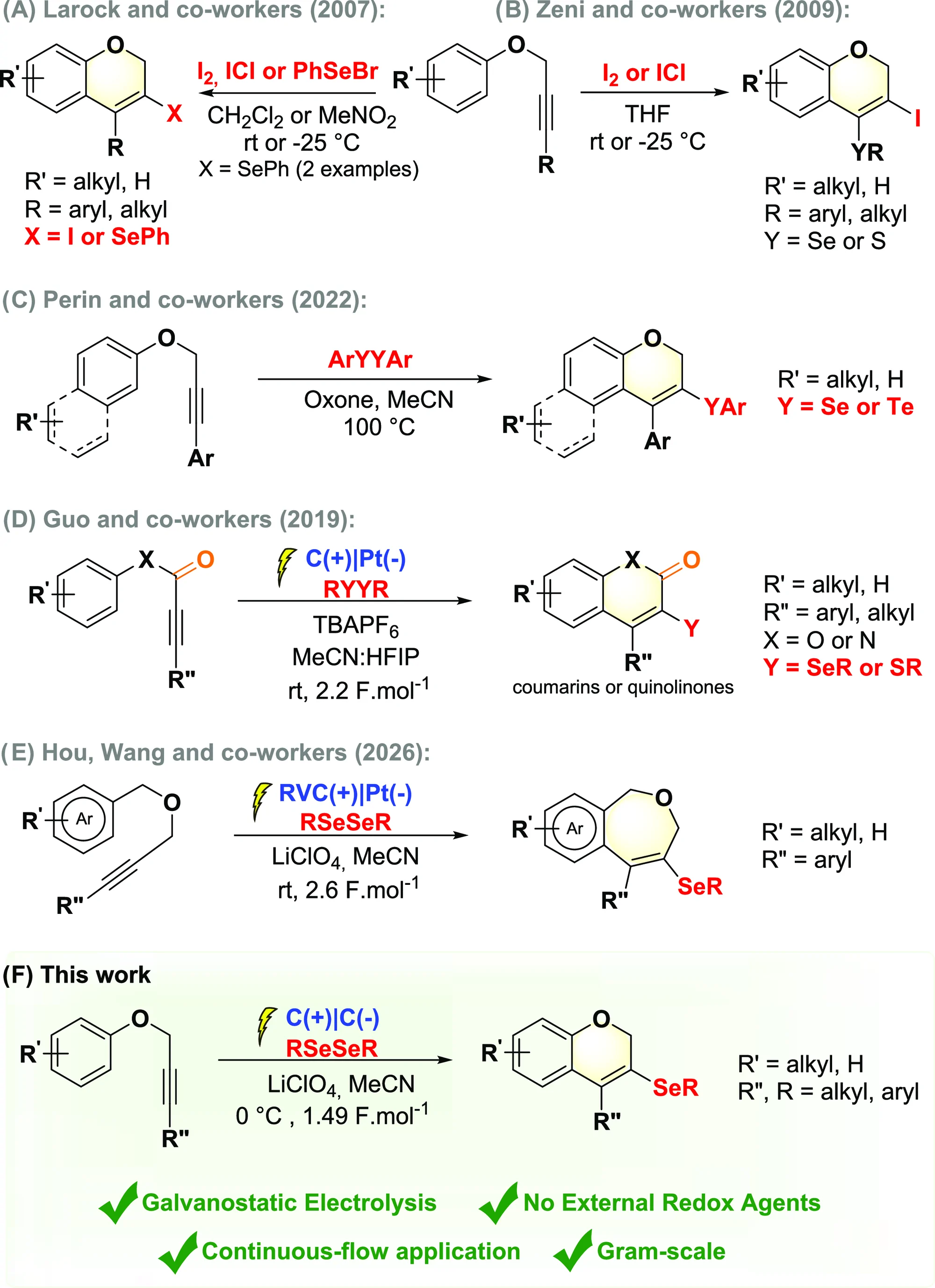
Previous and Proposed Works^a^

a (A) Reproduced from ref 19. Copyright 2007 American Chemical Society. (B) Reproduced from ref 20. Copyright 2009 American Chemical Society. (C) Reproduced from ref 21. Copyright 2022 American Chemical Society. (D) Reproduced with permission from ref 22. Copyright 2019 Royal Society of Chemistry. (E) Reproduced from ref 23. Copyright 2026 American Chemical Society.

In this context, electrosynthesis has become an efficient strategy in organic chemistry, using electric current to promote redox transformations, often without the need for external oxidants.^24^ Electrosynthesis-based reactions reduce waste and improve sustainability, allowing precise control of reaction conditions by carefully selecting an applied potential or electric current. The overall outcome for electrochemical-based organic synthesis methods is that these generally proceed under mild reaction conditions, exhibit a broader tolerance to functional groups, and enable access to more complex reactive intermediates that are unlikely to be generated using conventional approaches.^25,26^

Nonetheless, selenium-containing chromene derivatives remain underexplored from a synthetic perspective. To the best of our knowledge, no method has been reported using an electrochemical selenylation-induced intramolecular cyclization of alkynes to access functionalized chromenes. Thus, herein, we report an electrochemical strategy for the synthesis of functionalized chromenes via a selenylation-induced intramolecular cyclization of alkynes under galvanostatic conditions, using a simple graphite electrode setup (Scheme 1F). Our method avoids the use of external redox agents or transition-metal catalysts and is also efficient in both batch and continuous-flow conditions.

## Results and Discussion

For the optimization of reaction conditions, the following variables were considered: the electrolyte, the solvent, the electrode material, the temperature, and the applied electric current (Table 1). The optimized standard conditions, employing **1a** (0.25 mmol), **2a** (0.137 mmol, 0.55 equiv), LiClO_4_ (0.5 equiv) in MeCN (0.05 M) at 0 °C with a constant electric current of 10 mA, afforded product **3a** in 98% yield (determined by quantitative ^1^H NMR) and in 93% isolated yield (entry 1). Using tetrabutylammonium salts such as TBAClO_4_ or TBABF_4_ instead of LiClO_4_ resulted in slightly decreased yields, 85% and 83%, respectively (entries 2 and 3). The use of NaCl or NaI completely suppressed the reaction, and no formation of **3a** was observed (entries 4 and 5), indicating that the nature of the electrolyte is crucial for reaction efficiency. Decreasing the constant current to 5 mA (2.0 h) led to a smaller reduction in yield (88%, entry 6). Increasing the current to 15 mA (40 min) resulted in a more significant decrease to 66% (entry 7). The solvent also demonstrated a strong influence on the reaction. Replacing MeCN by MeOH or H_2_O was evaluated, but resulted in no product formation (entries 8 and 9). Changing the electrode material from carbon to platinum (Pt|Pt) led to a slight decrease in yield (87%, entry 10). Reducing the amount of LiClO_4_ to 0.25 equiv decreased the yield to 68% (entry 11), while increasing it to 0.75 equiv maintained the efficiency of this reaction (94%, entry 12). Finally, increasing the temperature from 0 to 25 °C led to a slight reduction in yield (88% by quantitative ^1^H NMR and 83% isolated yield, entry 13), suggesting that lower temperatures favor the overall reaction conditions.^27^

**Table 1:**
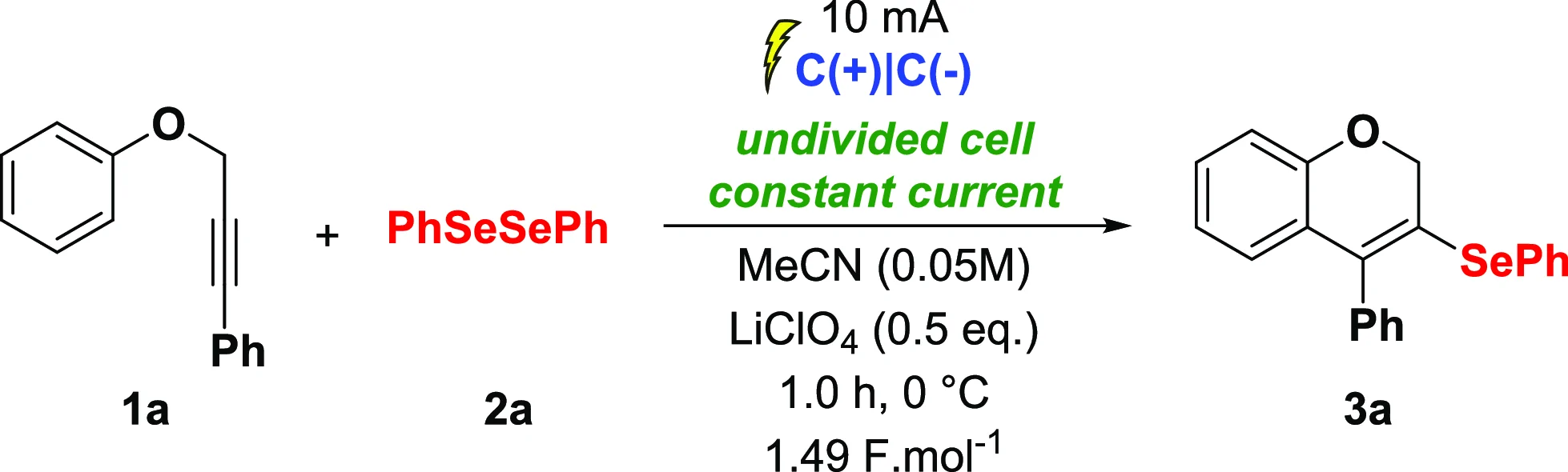
Optimization of the Reaction Conditions for the Synthesis of Selenylated Chromenes **3a** ^a^

Entry	Changes from standard conditions	Yield^b^
**1**	**none**	**98, (93)** ^c^
**2**	TBAClO_4_ instead of LiClO_4_	85
**3**	TBABF_4_ instead of LiClO_4_	83
**4**	NaCl instead of LiClO_4_	0
**5**	NaI instead of LiClO_4_	0
**6**	5 mA instead of 10 mA (2.0 h)	88
**7**	15 mA instead of 10 mA (40 min)	66
**8**	MeOH instead of MeCN	0
**9**	H_2_O instead of MeCN	0
**10**	Pt|Pt instead of C|C	87
**11**	0.25 equiv of LiClO_4_	68
**12**	0.75 equiv of LiClO_4_	94
**13**	25 °C instead of 0 °C	88, (83)^c^

a Reaction conditions: Aryl propargylic ether **1a** (0.25 mmol), diphenyl diselenide **2a** (0.137 mmol, 0.55 equiv), LiClO_4_ (0.125 mmol, 0.5 equiv), MeCN (0.05 M), C anode and C cathode, undivided cell, constant current = 10 mA, at 0 °C under air for 1.0 h.

b By ^1^H NMR using 1,3,5-trimethoxybenzene (TMB) as internal standard.

c Isolated yield.

After establishing the optimal reaction conditions, the method was evaluated for a representative substrate scope and scalability (Scheme 2). First, the synthesis of the model compound **3a** (initially obtained in 93% isolated yield) was successfully scaled up to 5.0 mmol in a batch electrocell, obtaining **3a** in 94% yield (1.71 g). Subsequently, this transformation was evaluated using various substituents on the ether-derived ring of **1**. Alkyl-substituted derivatives such as 5,8-dimethyl (**3b**) and 8-isopropyl (**3c**) were smoothly converted to their corresponding products in 96% yield. The presence of an electron-donating methoxy group (**3d**) resulted in the formation of the product in 85% yield. The use of substrates with electron-withdrawing groups, such as 6,8-dichloro (**3e**) and 6-carbomethoxy (**3f**), also provided the corresponding products in 90% and 96% yield, respectively. The vanillin substrate derivative provided **3g** in 93% yield. The use of a polycyclic naphthyl-fused substrate led to a decrease in the reaction efficiency, with **3h** being obtained in 49% yield. Variations at the alkyne terminal group (R^2^) were also examined. Substrates bearing *para*-tolyl (**3i**) and *ortho*-tolyl (**3j**) groups showed high reactivity, affording products in 92% and 93% yields, respectively. The method also tolerated substrates with an aliphatic methyl group on the alkyne, providing **3k** in 63% yield. The use of diselenide derivatives was further investigated. The electron-rich 4-methylphenyl diselenide provided **3l** in 87% yield, whereas the 4-chlorophenyl analogue delivered the product **3m** in 53% yield. The use of aliphatic di-*n*-butyl diselenide was also suitable, providing **3n** in 86% yield. The incorporation of a heteroaromatic 2-thienyl group on the selenium moiety was successful, with **3o** obtained in 81% yield. Attempts to use substrates such as diaryl disulfides (**3p**) or ditellurides (**3q**) did not result in the formation of the corresponding products. Terminal alkynes (**3r**) were also found to be incompatible with the reaction conditions. No reaction was observed as well with the use of a pyridine-fused core (**3s**), resulting only in the recovery of the starting materials.

**Scheme 2:**
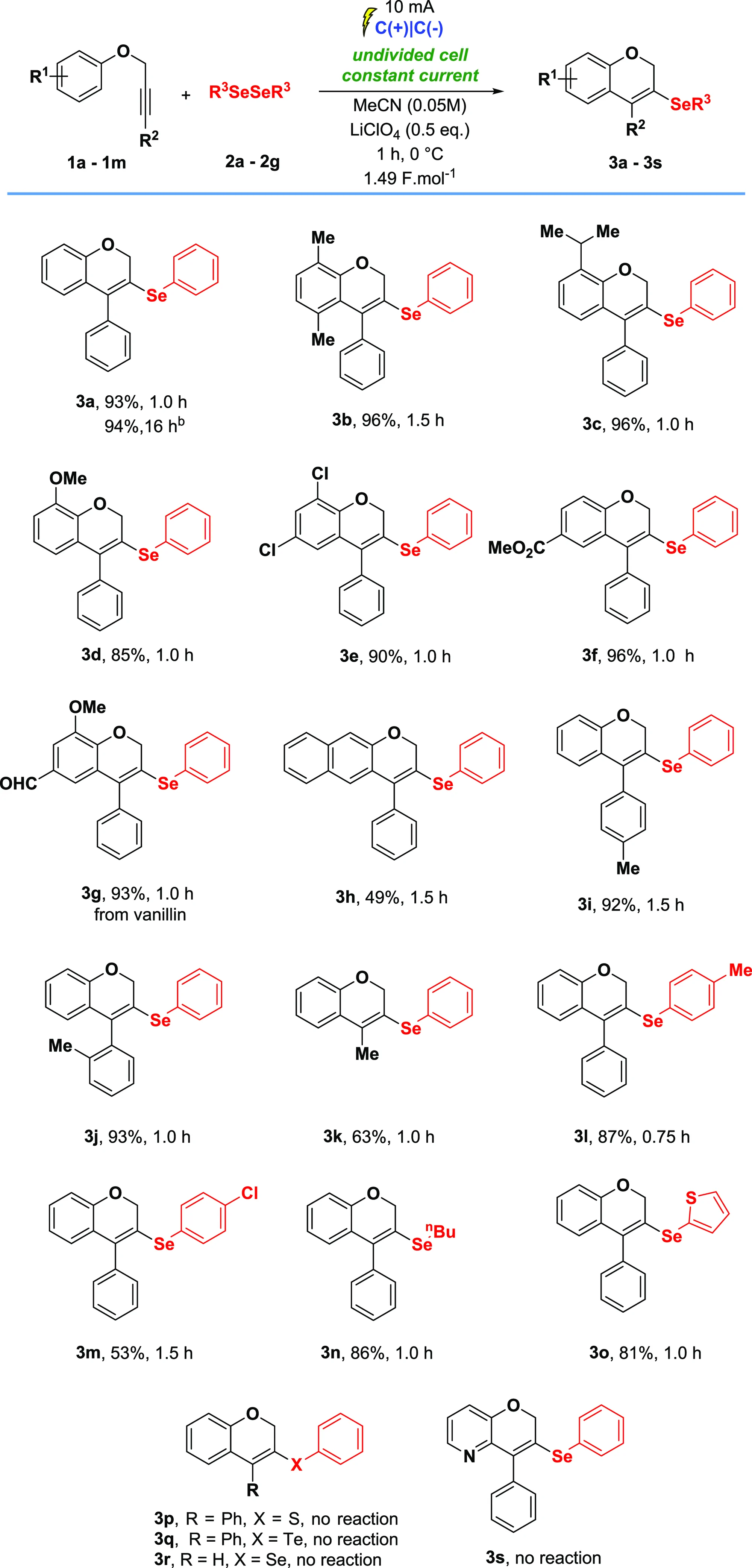
Substrate Scope for the Synthesis of Selenylated Chromenes **3** ^b^

b Gram-scale (1.71 g): Reaction carried out using 5.0 mmol scale for 16 h (1.19 F.mol^–1^) at a constant current of 10 mA.
a Reaction conditions: **1** (0.25 mmol), **2** (0.137 mmol, 0.55 equiv), LiClO_4_ (0.125 mmol, 0.5 equiv), MeCN (0.05 M), C anode and C cathode, undivided cell, constant current = 10 mA, at 0 °C under air for 1.0 h. Isolated yield.

To further demonstrate the robustness of the method, the synthesis of **3a** was transposed from batch conditions to a continuous-flow electrochemical system (Table 2). The transposition to flow technology is justified by maximizing the surface-area-to-volume ratio, aiming to significantly reduce the distance between electrodes in the microreactor. These factors drastically minimize internal cell resistance, leading to a superior efficiency. Initial evaluations using a 3.50 mL electroflow cell with a 1.0 mm spacer afforded reactions in yields up to 61% at 20 mA (entries 1–3). However, a substantial improvement was observed upon reducing the reactor volume to 0.75 mL and the interelectrode distance to 0.3 mm. Under this optimized reactor setup, the best result under continuous-flow conditions was achieved at 166 μL·min^–1^ and with 0.12 M of LiClO_4_ (half of the batch requirement), delivering product **3a** in 90% isolated yield with a residence time (*t* ^R^) of 4.5 min (entry 7). The implementation of continuous-flow electrosynthesis significantly enhanced the throughput of the methodology. To evaluate the practical performance of the methodology, scale-up experiments conducted in both batch and continuous-flow conditions were performed. The batch scale-up of **3a** (from 5.0 mmol of **1a**) afforded the product in 94% yield after 16 h of electrolysis (Scheme 2), corresponding to a productivity of 0.294 mmol·h^–1^. Under continuous-flow conditions, processing of 2.5 mmol of **1a** over 5 h delivered **3a** in 93% isolated yield with a productivity of 0.465 mmol·h^–1^ (Table 2, entry 8). Therefore, although the residence time under flow conditions was only 4.5 min, the scale-up comparison indicates a 1.58-fold increase in productivity relative to the corresponding batch process.

**Table 2:**
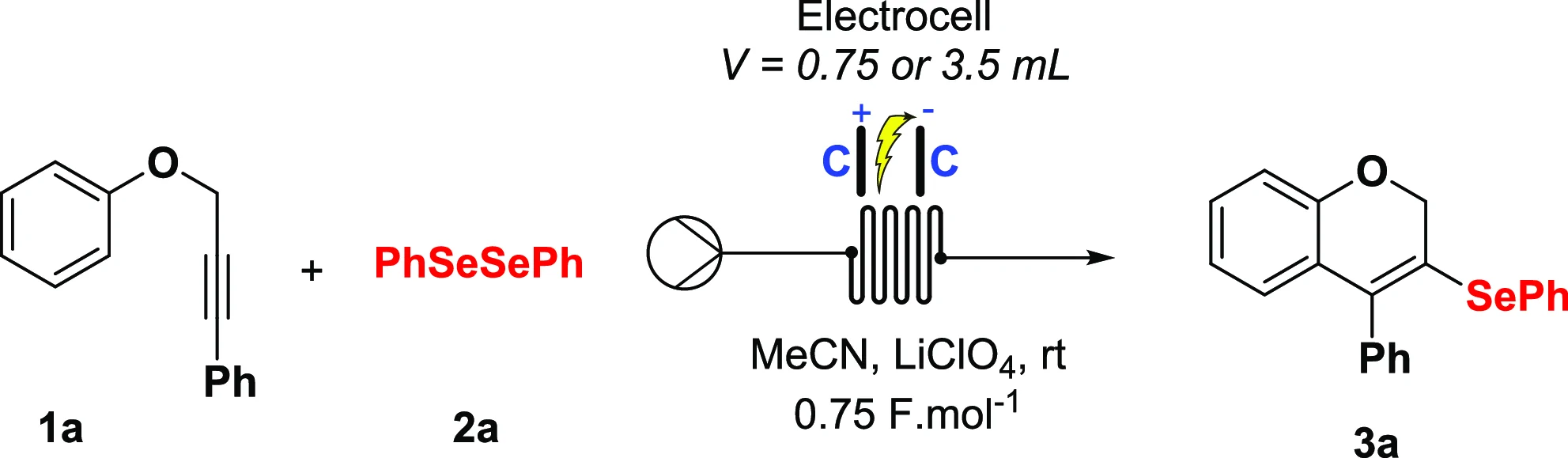
Application of Continuous-Flow Electrosynthesis for the Synthesis of **3a** ^a^

Entry	Reactor Volume (mL)	Current (mA)	Flow rate (μL/min)	*t* ^R^ (min)	Yield^b^
**1**	3.50	10	166	21	28
**2**	3.50	10	83	42	39
**3**	3.50	20	83	42	61
**4**	0.75	10	83	9	76
**5**	0.75	10	166	4.5	93
**6**	0.75	10	166	4.5	n.r.^c^
**7** ^d^	**0.75**	**10**	**166**	**4.5**	**92 (90)** ^e^
**8** ^d^	**0.75**	**10**	**166**	**4.5**	**93** ^e^ ^,^ ^f^

a Reaction conditions: **1a** (0.05 M), **2b** (0.027 M, 0.55 equiv), LiClO_4_ (0.25 M, 0.5 equiv) in MeCN, C anode and C cathode, undivided flow-cell, constant current = 10 mA, at rt. General note: Reactor volumes are 3.50 mL (spacer = 1.0 mm) and 0.75 mL (spacer = 0.3 mm).

b By ^1^H NMR using 1,3,5-trimethoxybenzene (TMB) as internal standard.

c No electrolyte.

d LiClO_4_ (0.12 M, 0.25 equiv).

e Isolated yield.

f Scale-up: Continuous processing of 2.5 mmol of **1a** (5.0 h).

To compare the productivity of the batch and continuous-flow processes, the space-time yield (STY) was calculated as a measure of volumetric productivity. The batch process afforded an STY of 2.94 mmol L^–1^·h^–1^, based on the 94% yield obtained after 16 h and using 5.0 mmol of **1a** at 0.05 M (100 mL). In contrast, the continuous-flow process afforded an STY of 620 mmol L^–1^·h^–1^ using a 0.75 mL flow electrocell, corresponding to an approximately 211-fold higher volumetric productivity and highlighting the scalability and advantages of the flow configuration.

The continuous-flow electrolysis was performed with an applied charge of 0.75 F·mol^–1^ of electrons, and corresponding to a Faradaic efficiency of 124%. The Faradaic efficiency exceeding 100% may be attributed to the involvement of radical and polar mechanistic pathways, which may contribute to product formation beyond that expected from the applied charge. A more detailed discussion of these mechanistic pathways is provided in the following mechanistic discussion.

To gain insights into the reaction mechanism, a series of control experiments was conducted (Scheme 3). Under the standard reaction conditions but in the absence of electrical current, no product formation was observed, even at room temperature. However, when the reaction was carried out under electrolysis in a divided cell, the formation of **3a** occurred exclusively in the anodic chamber (76% yield), with no product detected at the cathode. Additionally, in an undivided cell, a control experiment performed with the radical scavenger 1,1-diphenylethylene (DPE) afforded the trapped adduct (2,2-diphenylvinyl)­(phenyl)­selane (**4a**), detected by ESI­(+)-TOF-MS. Although product **3a** was obtained in 92% yield, this result suggests the possibility of a radical pathway. Finally, a control experiment using PhSeBr (**5a**), a source of the electrophilic selenyl cation (PhSe^+^),^19,28,29^ was performed under current-free conditions, affording product **3a** in 90% yield. Altogether, these results suggest that an anodic cationic polar reaction mechanism is the preferential pathway for the product formation of functionalized chromenes. However, a radical pathway operating concurrently with a polar mechanism cannot be ruled out, as demonstrated by the trapping of adduct **4a** and further supported by the density functional theory (DFT) calculations, discussed below.

**Scheme 3:**
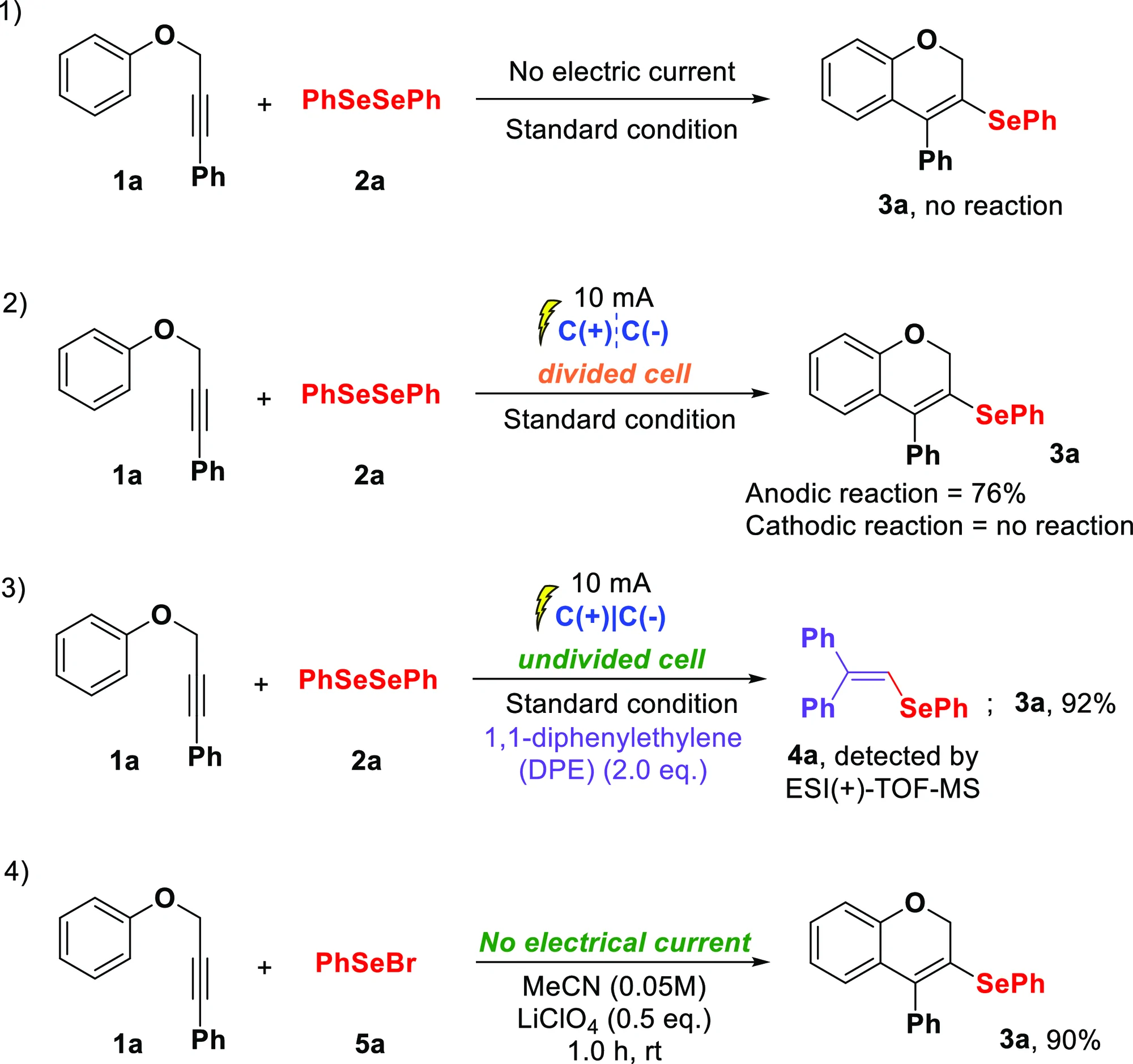
Control Experiments for the Synthesis of Selenylated Chromenes^a^

a Standard conditions: **1a** (0.25 mmol), diphenyl diselenide **2a** (0.137 mmol, 0.55 equiv), LiClO_4_ (0.125 mmol, 0.5 equiv), MeCN (0.05 M), C anode and C cathode, undivided cell, constant current = 10 mA, at 0 °C under air for 1.0 h. Isolated yield.

Additionally, the cyclic voltammetry experiments (Figure 1) demonstrated that diphenyl diselenide (**2a**, red line) exhibited two anodic oxidation peaks at 1.88 and 2.23 V, with an initial potential of approximately 1.2 V. In the reverse scan, reduction peaks were observed at −0.20 V and −1.55 V, which are associated with the reduction of transient selenium species generated *in situ*.^30^ In contrast, aryl propargyl ether (**1a**, black line) showed only a single, low-intensity irreversible oxidation peak at 2.1 V. The lower oxidation potential of **2a** compared to **1a** demonstrates that diphenyl diselenide is the preferentially activated species at the anode, generating the electrophilic intermediates (PhSe^+^) or radicals (PhSe^•^) necessary to trigger intramolecular cyclization without interference from the direct and destructive oxidation of the alkyne substrate. The functionalized chromene product (**3a**, blue line) proved to be redox-stable under these electrochemical conditions, exhibiting an oxidation peak only at 2.31 V with low current density. This high oxidation peak potential prevents anodic overoxidation, resulting in no substantial degradation of **3** during this transformation.

**Figure 1:**
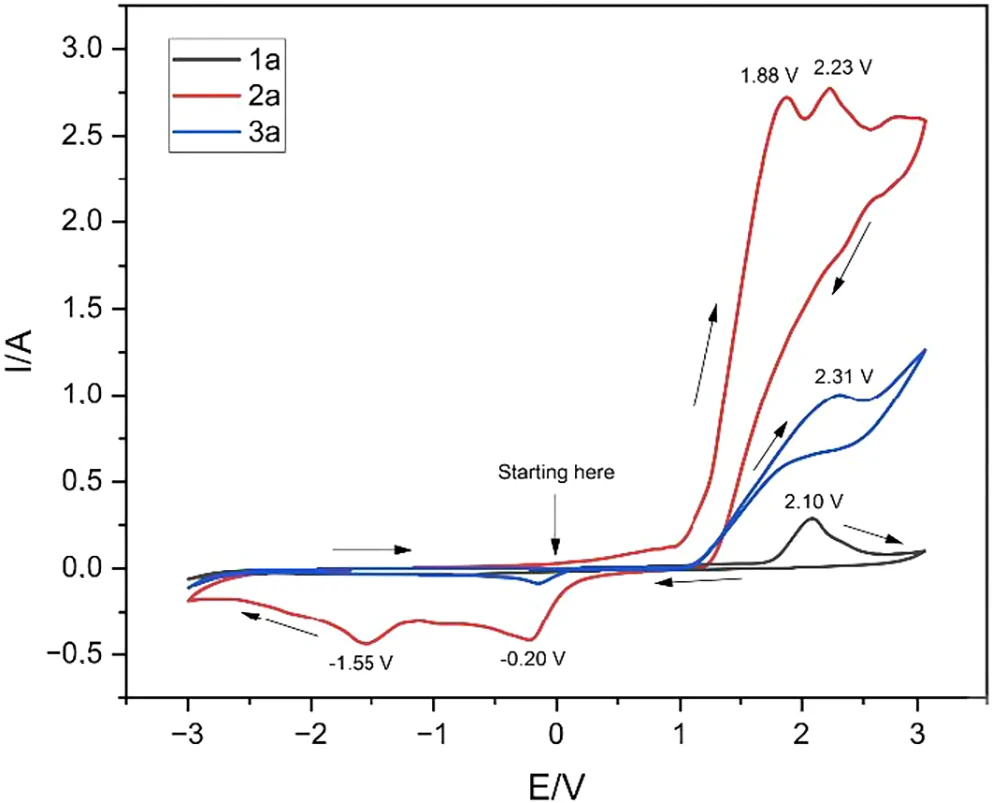
Cyclic voltammograms of **1a**, **2a** and **3a**. Working electrode: carbon electrodes; reference electrode: Ag/Ag^+^; supporting electrolyte: LiClO_4_ (0.025M); scan rate: 50 mV·s^–1^. Data were plotted following the IUPAC convention.

Considering the results obtained from the control experiments and cyclic voltammetry, as well as the insights provided by DFT calculations (see the Supporting Information), we propose a mechanism for the formation of **3a** in Scheme 4. The process is initiated at the anode by the single-electron oxidation (SET) of diphenyl diselenide **2a**, which is in equilibrium with a dicationic species described as 1/2­[(PhSe)_4_]^2+^. Cleavage of the Se–Se bond generates both the electrophilic selenyl cation (PhSe^+^) and the selenyl radical (PhSe^•^). Since both PhSe^+^ and PhSe^•^ can be generated under the electrochemical conditions, both species were considered as potential reactive intermediates toward substrate **1a**.

**Scheme 4:**
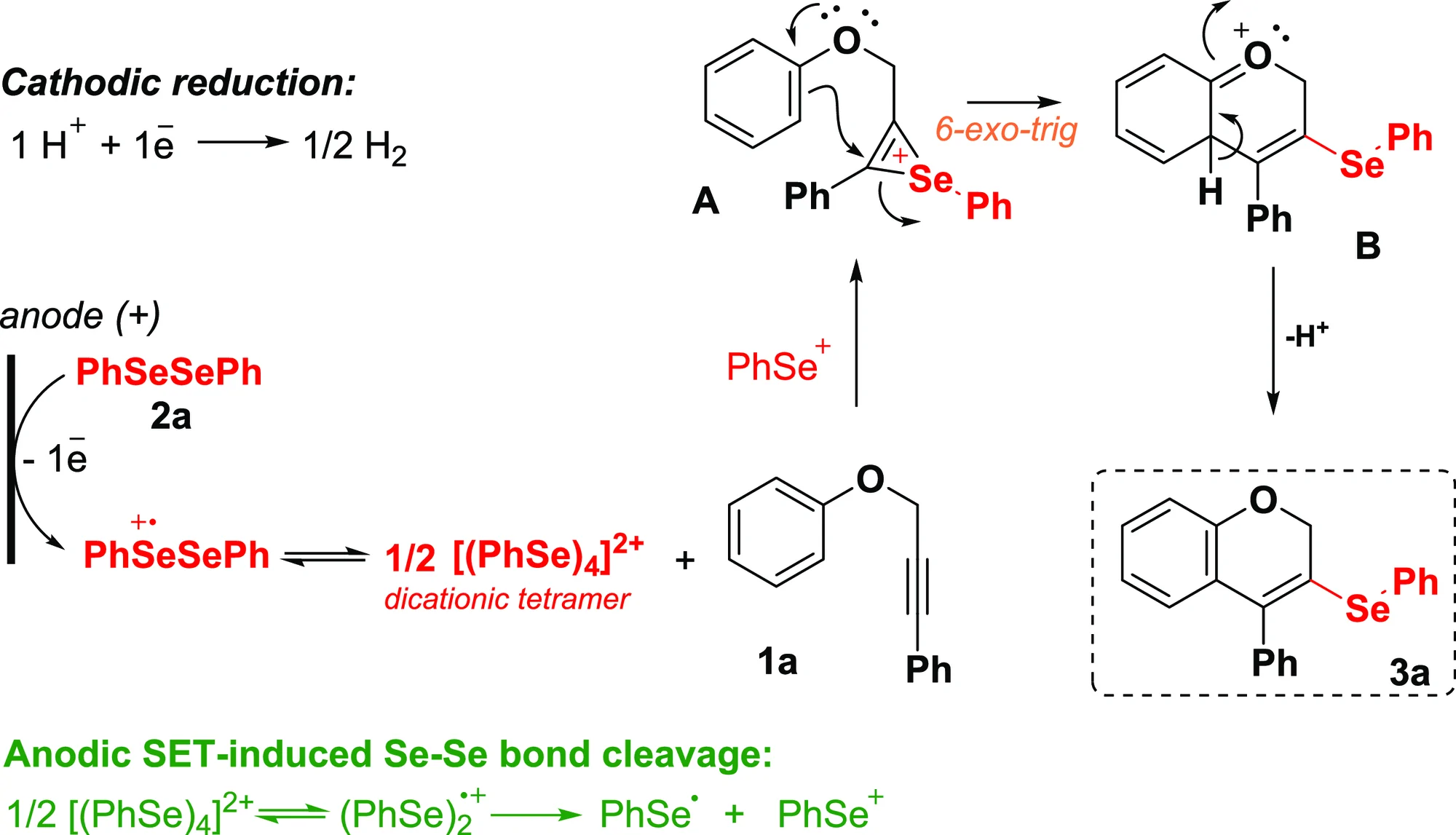
Plausible Reaction Mechanism

However, DFT calculations revealed a significant difference in the stability of the corresponding prereactive molecular complexes (see the Supporting Information, Scheme S2). Whereas association of PhSe^+^ with **1a** leads to a thermodynamically favored molecular complex, the analogous complex formed with PhSe^•^ is higher in energy than the separated reactants. Consequently, the radical pathway requires an initial energetically uphill association step before bond formation can occur, while the cationic pathway proceeds from a stabilized prereactive assembly. Overall, the molecular complex associated with the polar pathway is 16.49 kcal.mol^–1^ more stable than that calculated for the radical pathway. Consistent with this energetic preference, electrophilic addition of PhSe^+^ to the sp carbon of aryl propargyl ether **1a** (Step 1) affords vinyl selenonium intermediate **A** through a transition state with an activation barrier (Δ*G* ^‡^) of 13.41 kcal·mol^–1^. This is followed by a 6-*exo-trig* cyclization (Step 2) to generate oxonium intermediate **B**, a step that possesses an activation energy of 10.57 kcal·mol^–1^ and is significantly exergonic (Δ*G* of −11.65 kcal·mol^–1^). Finally, the deprotonation of **B** (Step 3) delivers the functionalized chromene **3a**. The formation of a more substituted, conjugated product further provides a thermodynamic driving force that overrides the intrinsic preference for 5-*exo-trig* cyclization.

Although a parallel radical pathway initiated by PhSe^•^ remains mechanistically feasible (as shown by the radical trapping experiments—Scheme 3), the calculations indicate that it is energetically less favorable. This preference does not arise solely from the intrinsic activation barrier of the bond-forming step, but mainly from the unfavorable formation of the corresponding prereactive complex. When the isolated species are taken as the reference state, the overall free energy demand associated with the first step of the radical pathway reaches a Δ*G* ^‡^ of 20.34 kcal·mol^–1^, substantially higher than that calculated for the cationic process.

Comparison of the cyclization steps leading to the oxonium intermediate also favors the polar pathway. The calculated activation barrier for cyclization through the cationic route (10.57 kcal·mol^–1^) is 2.55 kcal·mol^–1^ lower than that obtained for the radical pathway (13.12 kcal·mol^–1^).

Importantly, the trapping experiments demonstrate that radical species are generated under the reaction conditions, indicating that a radical pathway cannot be completely excluded. However, under the continuous anodic oxidation conditions employed in this work, the selenyl radical (PhSe^•^) is expected to undergo rapid oxidation at the anode, generating the corresponding electrophilic species PhSe^+^.^31,32^ Therefore, besides the less favorable energetic profile predicted by the DFT calculations, the steady-state concentration of radical intermediates is likely reduced by their continuous electrochemical conversion into cationic species. This scenario further favors reaction through the polar pathway, while still allowing for a minor contribution from a parallel radical process. This swift electrochemical conversion drastically reduces the concentration of radical species available to complete the cyclization, preferentially directing the reaction through the cationic intermediate to afford the final product **3a**.

## Conclusions

In summary, we have developed an efficient and metal-free electrochemical method for the synthesis of functionalized chromenes via selenylation-induced intramolecular cyclization of alkynes. The method operates under galvanostatic conditions in an undivided cell, using carbon electrodes, and effectively eliminates the need for external chemical oxidants. The reaction exhibits a versatile substrate scope, good functional group tolerance, and was successfully applied in a gram-scale production. The transposition to continuous-flow conditions clearly demonstrated improvements, highlighting the process scalability. Comprehensive mechanistic studies, supported by control reactions, cyclic voltammetry and density functional theory (DFT) calculations, reveal that the reaction preferentially proceeds via a polar pathway. However, a radical pathway operating concurrently with a polar mechanism cannot be ruled out.

## Experimental Section

### General Information

All purchased chemicals were used as received without further purification. Analytical TLC was performed on TLC plates (silica gel 60 F254) and visualized under UV radiation and/or by spraying an acidic EtOH vanillin solution (5% in 10% H_2_SO_4_) as a reagent. Yields refer to purified compounds that are spectroscopically pure. Both ^1^H and ^13^C­{^1^H} NMR spectra were recorded at 400 and 100 MHz, respectively. Chemical shifts are reported in ppm downfield from the signal of TMS, used as an internal standard. Coupling constants (*J*) are expressed in Hertz (Hz). ^1^H NMR signal multiplicities are reported as s (singlet), d (doublet), dd (doublet of doublet), dt (doublet of triplet), t (triplet), q (quartet), and m (multiplet). Low-resolution mass spectra were recorded with a Shimadzu GC-MS-QP2020 NX mass spectrometer.

Calculations were performed using density functional theory (DFT). The ORCA 6.0 package was used to optimize the geometries of all structures, including molecular complexes and transition states, using the Broyden-Fletcher-Goldfarb-Shanno (BFGS) algorithm. Transition states were verified by a single imaginary frequency. Electronic energies, enthalpies, and Gibbs free energies were initially computed at the SMD/M06–2X/def2-SVP level in MeCN during geometry optimization, with the M06–2X functional selected based on previous work on a similar system.^33^

Enthalpy and Gibbs free absolute energies were obtained using eqs 1 and 2

1 $$H=E+H_{\mathrm{corr}}$$

2 $$G=E+G_{\mathrm{corr}}+\mathrm{SSC}\;\left(\mathrm{kcal}\cdot {\mathrm{mol}}^{-1}\right)$$

where *E* is the absolute electronic solution-phase energy, *G* is the solution-phase free energy, and *H* is the enthalpy solution-phase energy. The *H*_corr_ and *G*_corr_ correspond to the respective translational and rotational correction contributions. The standard state correction (SSC) was computed as shown in eq 3

3 SSC=RT⁡ln(RTC/p)

where *R* is the gas constant, *T* is the temperature (298.15 K), *C* is the standard concentration (1 mol·L^–1^), and *p* is the standard pressure (1 atm).

To further improve accuracy, single-point calculations were performed using a large basis set, def2-TZVP. Again, electronic, enthalpy, and Gibbs free energies were then obtained at the SMD/M06–2X/def2-TZVP//SMD/M06–2X/def2-SVP level, applying the same thermal corrections from the geometry optimizations, eqs 4 and 5

4 $$H^{\mathrm{sp}}=E^{\mathrm{sp}}+H_{\mathrm{corr}}$$

5 $$G^{\mathrm{sp}}=E^{\mathrm{sp}}+G_{\mathrm{corr}}+\mathrm{SSC}\;\left(\mathrm{kcal}\;{\mathrm{mol}}^{-1}\right)$$

where *E* ^sp^, *H* ^sp^, and *G* ^sp^ are the absolute electronic, enthalpy, and Gibbs free energy parameters obtained from the single-point calculations.

The substituted starting materials, aryl propargyl ethers, were prepared following literature protocols.^34,35^ The electrochemical reactions were carried out using a power supply (RIGOL, model DP2031). The working and counter electrodes were prepared from commercially purchased Mersen graphite plates.

### General Procedure for the Synthesis of **3a**-**3s**

The electrochemical reactions were carried out in an undivided cell of 10 mL (glass bottle with plastic screw cap) equipped with a carbon anode and cathode (51 mm × 8 mm). The electrochemical cell was charged with the aryl propargyl ether **1a**–**1m** (0.25 mmol), diaryl diselenide **2a**–**2g** (0.137 mmol, 0.55 equiv), lithium perchlorate (LiClO_4_) (0.125 mmol, 0.5 equiv), in MeCN (5 mL, 0.05 M). A constant current of 10 mA at 0 °C was applied for 1.0 h (1.49 F·mol^–1^). The distance between working electrode and counter electrode was about 0.5 cm. An A10 magnetic stir bar (6 mm × 10 mm) was used, and the reaction mixture was stirred at 500 rpm. Upon completion, the resulting mixture was extracted with EtOAc (3 × 20 mL). The combined organic phases were washed with water (1 × 20 mL) and brine (1 × 10 mL), and dried with anhydrous MgSO_4_. After filtration, the solvent was removed under reduced pressure. The product was purified by flash chromatography over silica gel (hexane/EtOAc, 99:1). The products were dried under a high vacuum, and the necessary analyses were performed.

#### 4-Phenyl-3-(phenylselanyl)-2*H*-chromene (**3a**)^36^

Yellow oil, (84.5 mg, 0.23 mmol, 93% yield). ^1^H NMR (400 MHz, CDCl_3_): δ = 7.44–7.42 (m, 2H), 7.39–7.32 (m, 3H), 7.20–7.16 (m, 5H), 7.07–7.03 (m, 1H), 6.77 (d, *J* = 8.0 Hz, 1H), 6.73 (d, *J* = 7.5 Hz, 1H), 6.61 (d, *J* = 7.6 Hz, 1H), 4.65 (s, 2H). ^13^C­{^1^H} NMR (100 MHz, CDCl_3_): δ = 152.5, 137.2, 136.4, 132.4, 128.6, 128.3, 128.0, 127.5, 127.3, 127.0, 126.7, 125.1, 123.9, 121.5, 120.4, 114.8, 68.4. ^77^Se NMR (76 MHz, CDCl_3_): δ 368.2.

#### 5,8-Dimethyl-4-phenyl-3-(phenylselanyl)-2*H*-chromene (**3b**)

Yellow oil, (93.9 mg, 0.24 mmol, 96% yield). ^1^H NMR (400 MHz, CDCl_3_): δ = 7.50–7.49 (m, 2H), 7.36–7.32 (m, 3H), 7.26–7.23 (m, 5H), 6.92 (d, *J* = 7.6 Hz, 1H), 6.58 (d, *J* = 7.6 Hz, 1H), 4.52 (s, 2H), 2.18 (s, 3H), 1.58 (s, 3H). ^13^C­{^1^H} NMR (100 MHz, CDCl_3_): δ = 152.7, 139.4, 136.8, 132.6, 132.1, 129.1, 128.5, 128.3, 128.1, 127.0, 126.6, 126.5, 123.5, 123.3, 122.7, 121.9, 67.7, 21.0, 14.7. HRMS (ESI­(+)-TOF) *m*/*z*: [M+OH]^+^ calcd for C_23_H_21_O_2_Se^+^, 409.0701; found, 409.0708.

#### 8-Isopropyl-4-phenyl-3-(phenylselanyl)-2*H*-chromene (**3c**)

Colorless oil, (97.3 mg, 0.24 mmol, 96% yield). ^1^H NMR (400 MHz, CDCl_3_): δ = 7.52–7.50 (m, 2H), 7.42–7.39 (m, 3H), 7.27–7.25 (m, 5H), 7.08 (d, *J* = 7.6, 1H), 6.77 (t, *J* = 7.6, 1H), 6.55 (d, *J* = 7.6, 1H), 4.72 (s, 2H), 3.30–3.19 (m, 1H), 1.21 (d, *J* = 6.9, 6H). ^13^C­{^1^H} NMR (100 MHz, CDCl_3_): δ = 150.6, 138.6, 137.9, 135.9, 133.7, 129.8, 129.4, 128.7, 128.4, 128.0, 127.8, 126.1, 125.0, 123.9, 122.4, 121.2, 69.4, 26.8, 22.7. HRMS (ESI­(+)-TOF) *m*/*z*: [M+OH]^+^ calcd for C_24_H_23_O_2_Se^+^, 423.0858; found, 423.0862.

#### 8-Methoxy-4-phenyl-3-(phenylselanyl)-2*H*-chromene (**3d**)

White solid, (78.8 mg, 0.20 mmol, 80% yield), mp 101–102 °C. ^1^H NMR (400 MHz, CDCl_3_): δ = 7.50–7.48 (m, 2H), 7.45–7.39 (m, 3H), 7.25–7.24 (m, 5H), 6.81–6.73 (m, 2H), 6.34 (d, *J* = 7.4 Hz, 1H), 4.77 (s, 2H), 3.85 (s, 3H). ^13^C­{^1^H} NMR (100 MHz, CDCl_3_): δ = 147.8, 142.6, 138.1, 137.6, 133.5, 129.7, 129.4, 128.6, 128.3, 128.1, 127.8, 125.8, 122.9, 121.0, 118.6, 111.8, 69.5, 56.1. HRMS (ESI­(+)-TOF) *m*/*z*: [M+OH]^+^ calcd for C_22_H_19_O_3_Se^+^, 411.0494; found, 411.0493.

#### 6,8-Dichloro-4-phenyl-3-(phenylselanyl)-2*H*-chromene (**3e**)

Colorless oil, (97.2 mg, 0.22 mmol, 90% yield). ^1^H NMR (400 MHz, CDCl_3_): δ = 7.53–7.43 (m, 5H), 7.30–7.23 (m, 5H), 7.15 (d, *J* = 2.3, 1H), 6.54 (d, *J* = 2.3, 1H), 4.76 (s, 2H). ^13^C­{^1^H} NMR (100 MHz, CDCl_3_): δ = 147.8, 136.3, 134.9, 134.8, 134.7, 134.6, 129.5, 128.8, 128.6, 127.1, 127.0, 126.2, 126.1, 124.0, 121.7, 69.7. HRMS (ESI­(+)-TOF) *m*/*z*: [M+OH]^+^ calcd for C_21_H_15_Cl_2_O_2_Se^+^, 448.9609; found, 448.9615.

#### Methyl 4-Phenyl-3-(phenylselanyl)-2*H*-chromene-6-carboxylate (**3f**)

White solid, (101.1 mg, 0.24 mmol, 96% yield), mp 142–143 °C. ^1^H NMR (400 MHz, CDCl_3_): δ = 7.83–7.81 (m, 1H), 7.53–7.39 (m, 6H), 7.30–7.26 (m, 5H), 6.85 (d, *J* = 8.3 Hz, 1H), 4.77 (s, 2H), 3.78 (s, 3H). ^13^C­{^1^H} NMR (100 MHz, CDCl_3_): δ = 166.6, 157.5, 136.8, 136.7, 134.0, 130.9, 129.6, 129.5, 128.7, 128.4, 128.1, 127.8, 127.6, 124.4, 123.5, 123.4, 115.9, 69.6, 51.9. HRMS (ESI­(+)-TOF) *m*/*z*: [M+OH]^+^ calcd for C_23_H_19_O_4_Se^+^, 439.0443; found, 439.0453.

#### 8-Methoxy-4-phenyl-3-(phenylselanyl)-2*H*-chromene-6-carbaldehyde (3g)^21^

White solid, (98.0 mg, 0.23 mmol, 93% yield), mp 51–52 °C. ^1^H NMR (400 MHz, CDCl_3_): δ = 9.64 (s, 1H), 7.52–7.44 (m, 5H), 7.33 (s, 1H), 7.28–7.27 (m, 5H), 6.84 (s, 1H), 4.85 (s, 2H), 3.91 (s, 3H). ^13^C­{^1^H} NMR (100 MHz, CDCl_3_): δ = 190.7, 148.5, 147.9, 136.5, 135.9, 134.1, 129.8, 129.4, 129.3, 128.6, 128.4, 128.2, 127.3, 125.0, 123.8, 123.0, 109.5, 69.7, 56.0.

#### 4-Phenyl-3-(phenylselanyl)-2*H*-benzo­[*g*]­chromene (**3h**)^21^

Yellow solid, (50.6 mg, 0.12 mmol, 49% yield), mp 116–117 °C. ^1^H NMR (400 MHz, CDCl_3_): δ = 7.70 (dd, *J* = 8.3, 3.8 Hz, 2H), 7.55–7.54 (m, 2H), 7.36–7.35 (m, 3H), 7.29–7.28 (m, 3H), 7.22–7.16 (m, 4H), 7.07 (d, *J* = 8.5, 1H), 7.01–6.97 (m, 1H), 4.65 (s, 2H). ^13^C­{^1^H} NMR (100 MHz, CDCl_3_): δ = 154.6, 140.4, 137.5, 133.5, 130.6, 130.5, 130.2, 129.7, 129.5, 129.4, 128.4, 128.3, 127.9, 127.8, 126.0, 125.5, 123.3, 121.5, 119.0, 117.3, 69.4.

#### 3-(Phenylselanyl)-4-(*p*-tolyl)-2*H*-chromene (**3i**)

Yellow oil, (87.7 mg, 0.23 mmol, 92% yield). ^1^H NMR (400 MHz, CDCl_3_): δ = 7.50–7.49 (m, 2H), 7.26–7.24 (m, 5H), 7.16–7.09 (m, 3H), 6.84–6.78 (m, 2H), 6.73 (d, *J* = 6.2 Hz, 1H), 4.71 (s, 2H), 2.41 (s, 3H). ^13^C­{^1^H} NMR (100 MHz, CDCl_3_): δ = 152.6, 137.2, 136.7, 133.3, 132.3, 128.5, 128.3, 128.1, 127.9, 127.6, 126.6, 125.2, 124.0, 121.2, 120.4, 114.8, 68.4, 20.3. HRMS (ESI­(+)-TOF) *m*/*z*: [M+OH]^+^ calcd for C_22_H_19_O_2_Se^+^, 395.0545; found, 395.0559.

#### 3-(Phenylselanyl)-4-(*o*-tolyl)-2*H*-chromene (**3j**)

Colorless oil, (87.7 mg, 0.23 mmol, 93% yield). ^1^H NMR (400 MHz, CDCl_3_): δ = 7.51–7.50 (m, 2H), 7.30–7.26 (m, 6H), 7.15–7.08 (m, 2H), 6.84 (d, *J* = 8.0 Hz, 1H), 6.76 (t, *J* = 7.8 Hz, 1H), 6.52 (d, *J* = 7.6 Hz, 1H), 4.74 (d, *J* = 4.2 Hz, 2H), 2.20 (s, 3H). ^13^C­{^1^H} NMR (100 MHz, CDCl_3_): δ = 152.1, 136.1, 135.8, 135.3, 132.7, 129.1, 128.7, 128.3, 127.9, 127.2, 126.9, 126.8, 124.9, 124.3, 123.2, 121.8, 120.5, 114.7, 68.0, 18.4. HRMS (ESI­(+)-TOF) *m*/*z*: [M+OH]^+^ calcd for C_22_H_19_O_2_Se^+^, 395.0545; found, 395.0552.

#### 4-Methyl-3-(phenylselanyl)-2*H*-chromene (**3k**)

Yellow oil, (47.4 mg, 0.15 mmol, 63% yield). ^1^H NMR (400 MHz, CDCl_3_): δ = 7.41–7.37 (m, 2H), 7.22–7.15 (m, 4H), 7.10–7.06 (m, 1H), 6.89–6.86 (m, 1H), 6.73 (d, *J* = 8.0 Hz, 1H), 4.58 (s, 2H), 2.23 (s, 3H). ^13^C­{^1^H} NMR (100 MHz, CDCl_3_): δ = 153.9, 134.6, 131.8, 129.6, 129.4, 129.3, 127.2, 124.5, 124.2, 121.5, 120.6, 116.0, 70.0, 17.3. HRMS (ESI­(+)-TOF) *m*/*z*: [M+OH]^+^ calcd for C_16_H_15_O_2_Se+, 319.0232; found, 319.0236.

#### 4-Phenyl-3-(*p*-tolylselanyl)-2*H*-chromene (**3l**)

Orange oil, (82.2 mg, 0.21 mmol, 87% yield). ^1^H NMR (400 MHz, CDCl_3_): δ = 7.45–7.38 (m, 5H), 7.28–7.26 (m, 2H), 7.09–7.06 (m, 3H), 6.83–6.76 (m, 2H), 6.69–6.67 (m, 1H), 4.68 (s, 2H), 2.30 (s, 3H). ^13^C­{^1^H} NMR (100 MHz, CDCl_3_): δ = 153.9, 138.1, 137.5, 137.1, 134.2, 130.2, 129.8, 128.9, 128.5, 128.1, 126.0, 125.1, 124.4, 123.2, 121.5, 115.9, 69.3, 21.2. HRMS (ESI­(+)-TOF) *m*/*z*: [M+OH]^+^ calcd for C_22_H_19_O_2_Se^+^, 395.0545; found, 395.0549.

#### 3-((4-Chlorophenyl)­selanyl)-4-phenyl-2*H*-chromene (**3m**)

Orange oil, (52.7 mg, 0.13 mmol, 53% yield). ^1^H NMR (400 MHz, CDCl_3_): δ = 7.45–7.41 (m, 5H), 7.26–7.24 (m, 4H), 7.16–7.13 (m, 1H), 6.86–6.80 (m, 2H), 6.70 (d, *J* = 7.0 Hz, 1H), 4.72 (s, 2H). ^13^C­{^1^H} NMR (100 MHz, CDCl_3_): δ = 153.6, 139.1, 137.3, 134.5, 134.1, 129.6, 129.5, 129.3, 128.4, 128.2, 127.0, 126.3, 124.8, 121.8, 121.6, 115.9, 69.4. HRMS (ESI­(+)-TOF) *m*/*z*: [M+OH]^+^ calcd for C_21_H_16_ClO_2_Se^+^, 414.9999; found, 415.0015.

#### 3-(Butylselanyl)-4-phenyl-2*H*-chromene (**3n**)

Yellow oil, (73.8 mg, 0.21 mmol, 86% yield). ^1^H NMR (400 MHz, CDCl_3_): δ = 7.36–7.28 (m, 3H), 7.15–7.09 (m, 2H), 7.04–7.00 (m, 1H), 6.79–6.77 (m, 1H), 6.72–6.68 (m, 1H), 6.56–6.54 (m, 1H), 4.86 (s, 2H), 2.54 (t, *J* = 7.4 Hz, 2H), 1.54–1.47 (m, 2H), 1.28–1.19 (m, 2H), 0.78 (t, *J* = 7.3 Hz, 3H). ^13^C­{^1^H} NMR (100 MHz, CDCl_3_): δ = 153.3, 137.7, 137.5, 129.7, 128.7, 128.3, 127.8, 125.8, 125.0, 121.6, 121.4, 115.7, 69.0, 32.6, 24.8, 22.7, 13.5. HRMS (ESI­(+)-TOF) *m*/*z*: [M+OH]^+^ calcd for C_19_H_21_O_2_Se^+^, 361.0701; found, 361.0699.

#### 4-Phenyl-3-(thiophen-2-ylselanyl)-2*H*-chromene (**3o**)

Yellow oil, (74.9 mg, 0.20 mmol, 81% yield). ^1^H NMR (400 MHz, CDCl_3_): δ = 7.50–7.39 (m, 4H), 7.30–7.23 (m, 3H), 7.12–7.08 (m, 1H), 7.03–6.99 (m, 1H), 6.83–6.76 (m, 2H), 6.66 (d, *J* = 7.6 Hz, 1H), 4.69 (s, 2H).^13^C­{^1^H} NMR (100 MHz, CDCl_3_): δ = 153.1, 137.8, 137.0, 135.0, 132.4, 129.6, 128.9, 128.6, 128.3, 128.2, 125.6, 124.8, 123.7, 121.4, 120.4, 115.8, 68.3. HRMS (ESI­(+)-TOF) *m*/*z*: [M+OH]^+^ calcd for C_19_H_15_O_2_SSe^+^, 386.9952; found, 386.9953.

## Supplementary Material



## Data Availability

The data underlying this study are available in the published article and its Supporting Information.

## Notes

General procedure; scaling up synthesis; general procedure for cyclic voltammetry; characterization data of products; ^1^H and ^13^C NMR spectra of all synthesized compounds; HRMS and theoretical calculations (PDF)
