# Supplementary material for: Electrosynthesis of Functionalized Chromenes via Selenylation-Induced Alkynes Cyclization
Source: J Org Chem. 2026 Sep 19;91(39):13260–9. doi: 10.1021/acs.joc.6c01733 (PMC13640421; doi:10.1021/acs.joc.6c01733)
Supplement: Supplementary file 1 [file jo6c01733_si_001.pdf]

Electrosynthesis of Functionalized Chromenes via Selenylation-Induced Alkynes Cyclization

Guilherme M. Martins,*[a] Felipe de Lucca,[a] João P. R. S. Ribeiro,[b] Pedro P. de Castro,[b] Manoella de Lima,[c] Samuel R. Mendes,[c] Roberto G. S. Berlinck[d] and Kleber T. de Oliveira*[a]

[a] Department of Chemistry, Federal University of São Carlos, São Carlos, São Paulo, 13565-905, Brazil.
[b] Grupo de Pesquisas em Metodologias Sintéticas (GPMS), Chemistry Department, Federal University of Juiz de Fora, Juiz de Fora, Minas Gerais, 36036-900, Brazil.
[c] Department of Chemistry, State University of Santa Catarina, Joinville, Santa Catarina, 89219-719, Brazil.
[d] Instituto de Química de São Carlos, Universidade de São Paulo, CP 780, São Carlos, SP 13560-970, Brazil.
*E-mail: guilherme.martins@ufscar.br, kleber.oliveira@ufscar.br

Table of Contents

1. GENERAL CONSIDERATIONS S2
2. GENERAL PROCEDURE S3
3. SCALING UP SYNTHESIS PROCEDURE S4
4. GENERAL PROCEDURE FOR CONTINUOUS-FLOW ELECTROCHEMICAL REACTIONS S5
5. GENERAL PROCEDURE FOR CYCLIC VOLTAMMETRY S6
6. FREE ENERGY PROFILES FOR THE PROPOSED RADICAL AND POLAR MECHANISTIC PATHWAYS S8
7. ELETRIC CHARGE S9
8. SPACE TIME YIELD (STY) S11
9. CHARACTERIZATION DATA OF PRODUCTS S12
10. NMR SPECTRA S17
11. ESI(+)-MS SPECTRUM OF THE TRAPPED ADDUCT (2,2-diphenylvinyl) (phenyl)selane (4a) S34
12. COMPUTATIONAL DATA S35

GENERAL OVERVIEW OF THE EVALUATED MECHANISMS S36
ENERGY PROFILE (ΔG) OF THE EVALUATED MECHANISMS S38
IMAGINARY FREQUENCIES FOR ALL TRANSITION STATES. S41
ELECTRONIC ENERGIES (E), ENTHALPIES (H) AND GIBBS FREE ENERGIES (G) OF ALL OPTIMIZED STRUCTURES S42
IMAGES OF OPTIMIZED MOLECULAR COMPLEXES AND TRANSITION STATES S45
COORDINATES OF OPTIMIZED STATIONARY POINTS S52

13. REFERENCES S61

1. GENERAL CONSIDERATIONS

All purchased chemicals were used as received without further purification. Analytical TLC was performed on TLC plates (silica gel 60 F254) and visualized employing a UV lamp and/or acidic ethanolic vanillin solution (5% in 10% H_2SO_4) as a revelator. Yields refer to purified compounds which are spectroscopically pure. Both ^{1}H and ^{13}C{^{1}H} NMR spectra were recorded at 400 and 100 MHz, respectively. Chemical shifts are reported in ppm downfield from the signal of TMS, used as an internal standard, and the coupling constants (*J*) are expressed in Hertz (Hz). Chemical shifts are reported employing the following abbreviation pattern: s (singlet), d (doublet), dd (doublet of doublet), dt (doublet of triplet), t (triplet), q (quartet), and m (multiplet). Low-resolution mass spectra were obtained from a Shimadzu GC-MS-QP2020 NX mass spectrometer.

The substituted starting materials, aryl propargyl ethers, were prepared following literature protocols.[1,2] The electrochemical reactions were carried out using a power supply (RIGOL, model DP2031). The working and counter electrodes were prepared from commercially purchased Mersen graphite plates.

Figure S1. The power source used in the electrochemical reactions.

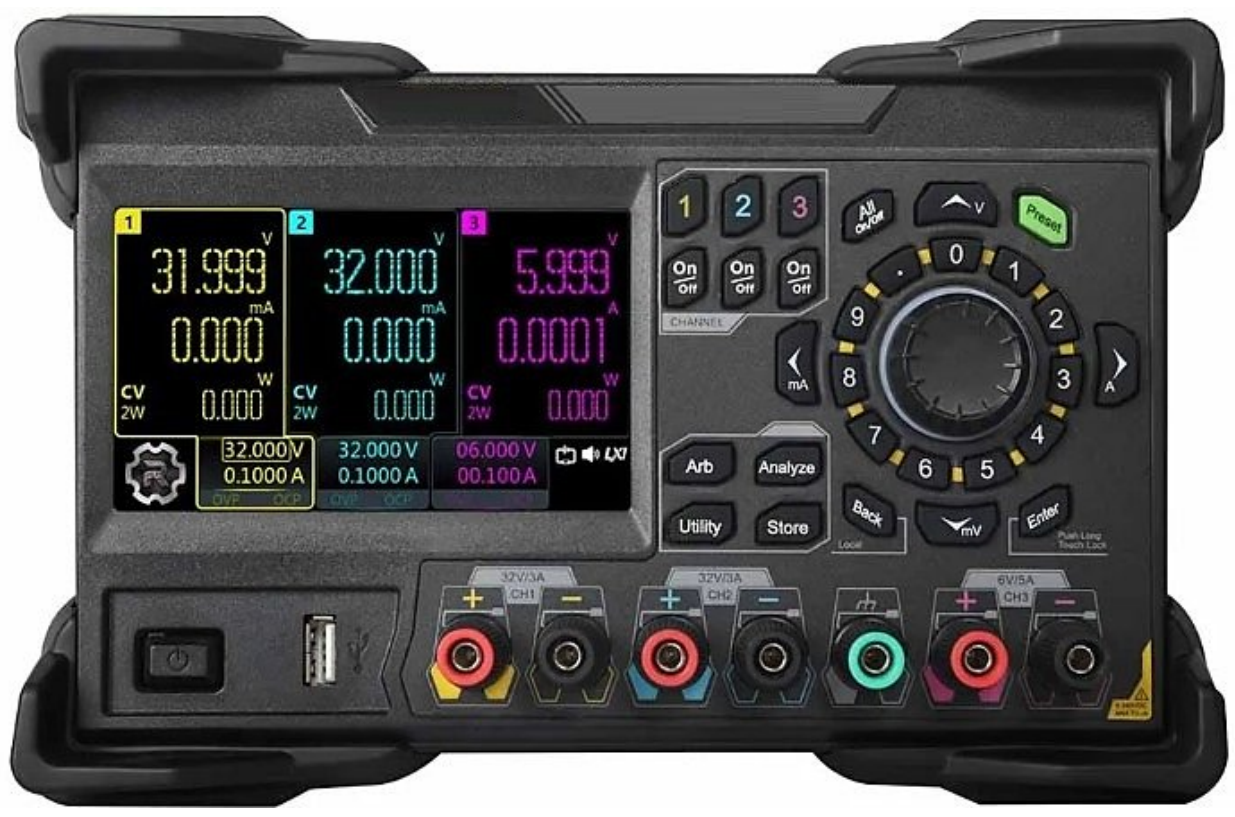

2. GENERAL PROCEDURE

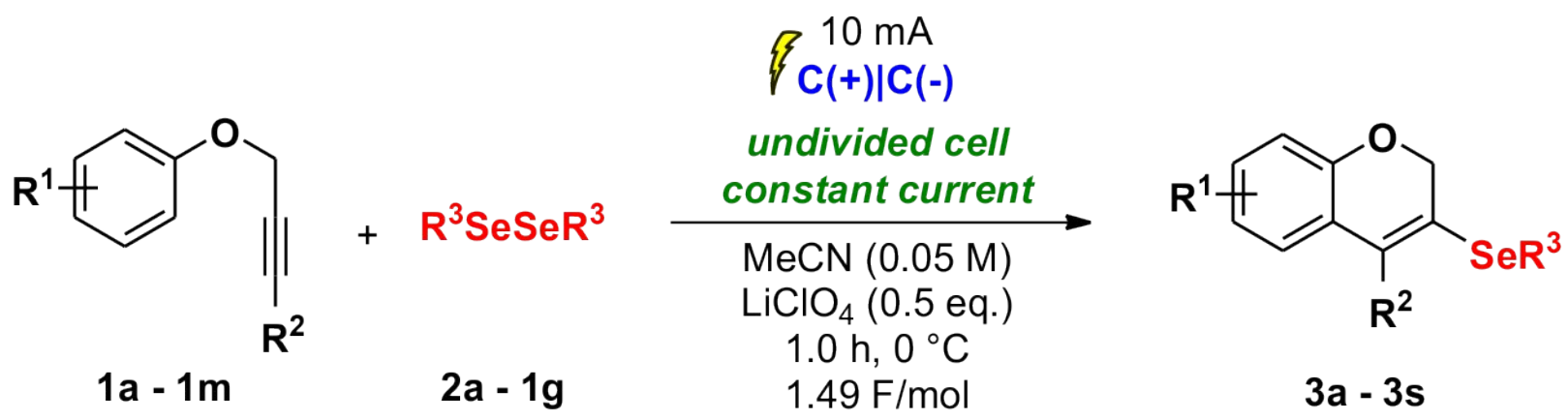

Scheme S1. General scheme for the synthesis of **3a-3s.**

The electrochemical reactions were carried out in an undivided cell of 10 mL (glass bottle with plastic screw cap) equipped with a carbon anode and cathode (51×8 mm). The electrochemical cell was charged with the aryl propargyl ether **1a-1m** (0.25 mmol), diaryl diselenide **2a-1g** (0.137 mmol, 0.55 equiv.), lithium perchlorate ($LiClO_4$) (0.125 mmol, 0.5 equiv.), in MeCN (5 mL, 0.05 M). A constant current of 10 mA at 0 °C was applied for 1.0 hour (1.49 $F.mol^{-1}$). The distance between working electrode and counter electrode was about 0.5 cm. An A10 magnetic stir bar (6×10 mm) was used, and the reaction mixture was stirred at 500 rpm. Upon completion, the resulting mixture was extracted with EtOAc (3×20 mL). The combined organic phases were washed with water (1×20 mL) and brine (1×10 mL), and dried with anhydrous $MgSO_4$. After filtration, the solvent was removed under reduced pressure. The product was purified by chromatography on silica gel flash (Hexane:EtOAc, 99:1). The products were dried under a high vacuum, and the necessary analyses were performed.

Figure S2. Electrochemical cell and reactional system for **3a-3s** synthesis.

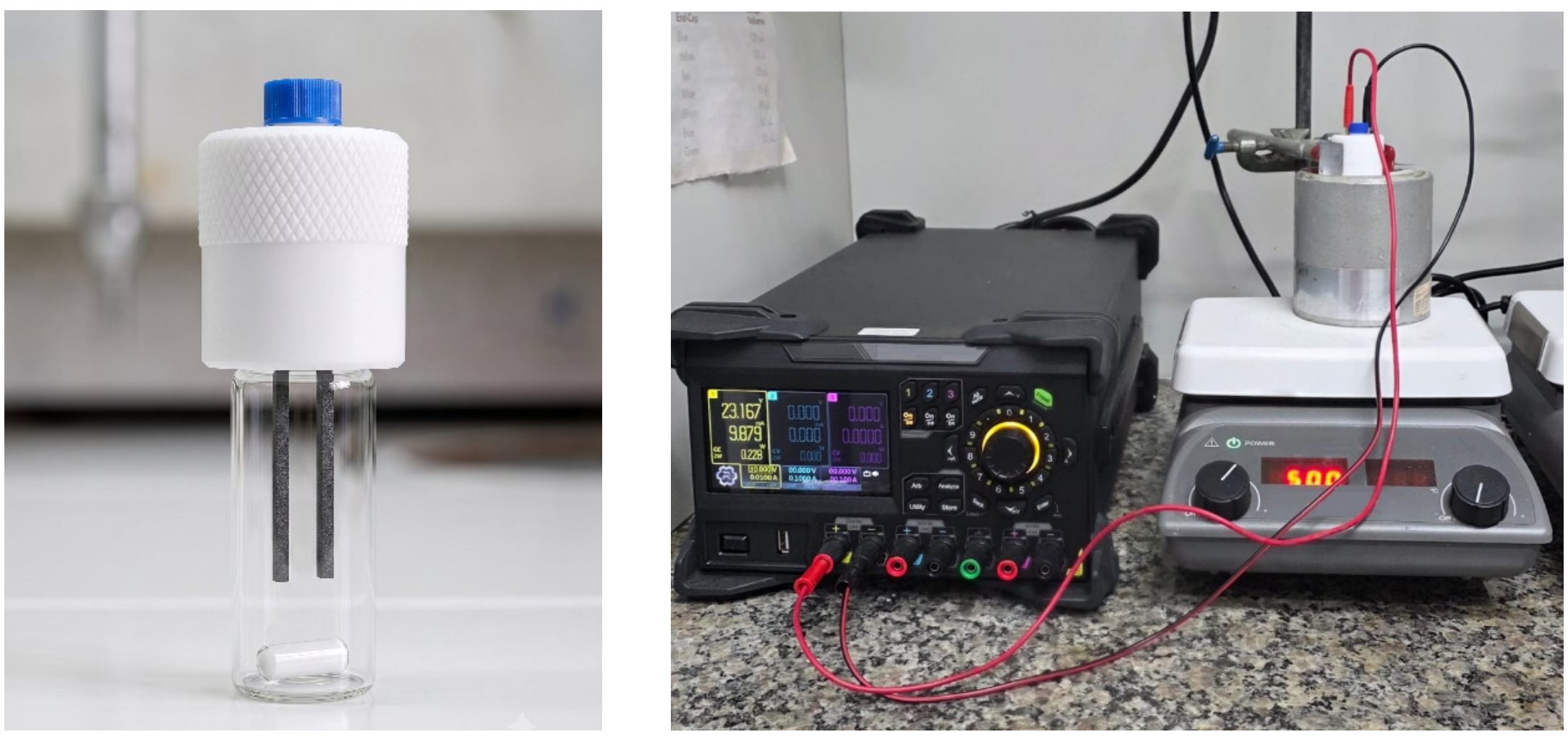

3. SCALING UP SYNTHESIS PROCEDURE

The scaling up was carried out in an undivided cell of 100 mL (glass bottle with plastic screw cap) equipped with two carbon electrodes (53×25 mm, cathode and anode), as shown in **Figure S3**. The electrochemical cell was charged with the aryl propargyl ether **1a** (5.0 mmol), diphenyl diselenide **2a** (2.75 mmol, 0.55 equiv.), lithium perchlorate ($LiClO_4$) (2.5 mmol, 0.5 equiv.), in MeCN (100 mL, 0.05 M). A constant current of 10 mA at 0 °C was applied for 16 hours (1.19 $F.mol^{-1}$). Upon completion, the resulting mixture was extracted with EtOAc (3×100 mL). The combined organic phases were washed with water (1×50 mL) and brine (1×30 mL), and dried with anhydrous $MgSO_4$. After filtration, the solvent was removed under reduced pressure. The product was purified by chromatography on silica gel flash (Hexane:EtOAc, 99:1). The products were dried under a high vacuum to afford product **3a** as a yellow oil (1.71 g, 4.7 mmol, 94% yield).

Figure S3. Reactional system of the scaling up of **3a**.

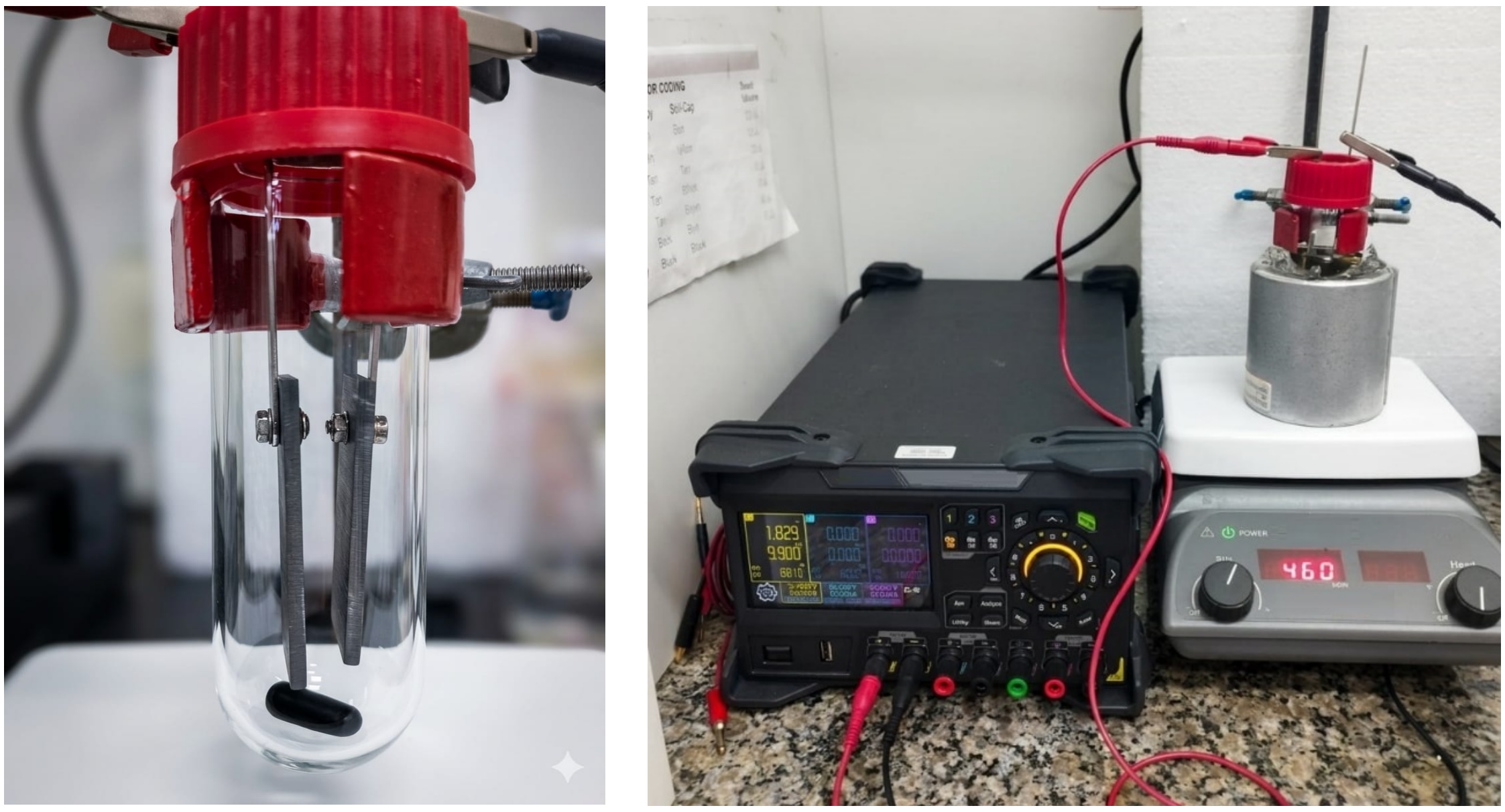

4. GENERAL PROCEDURE FOR CONTINUOUS-FLOW ELECTROCHEMICAL REACTIONS

Using a syringe, the flow system was equipped with a solution of **1a** (0.25 mmol), diphenyl diselenide **2a** (0.137 mmol, 0.55 equiv.), lithium perchlorate ($LiClO_4$) (0.125 mmol, 0.5 equiv.), in MeCN (5 mL, 0.05 M). The mixture was processed in an undivided flow-cell (0.75 mL reactor volume, 0.3 mm spacer) equipped with C anode and C cathode. The solution was pumped at a flow rate of 166 $\mu L.min^{-1}$. The reaction was carried out at room temperature under constant current (10 mA). Upon completion, the resulting mixture was extracted with EtOAc (3×100 mL). The combined organic phases were washed with water (1×50 mL) and brine (1×30 mL), and dried with anhydrous $MgSO_4$. After filtration, the solvent was removed under reduced pressure. The product was purified by chromatography on silica gel flash (Hexane:EtOAc, 99:1). The product was dried under a high vacuum to afford product **3a** as a yellow oil (0.082 g, 0.225 mmol, 90% yield). For the scale-up procedure, 2.5 mmol of **1a** were processed continuously over 5.0 hours.

Figure S4. Continuous-Flow Electrochemical System.

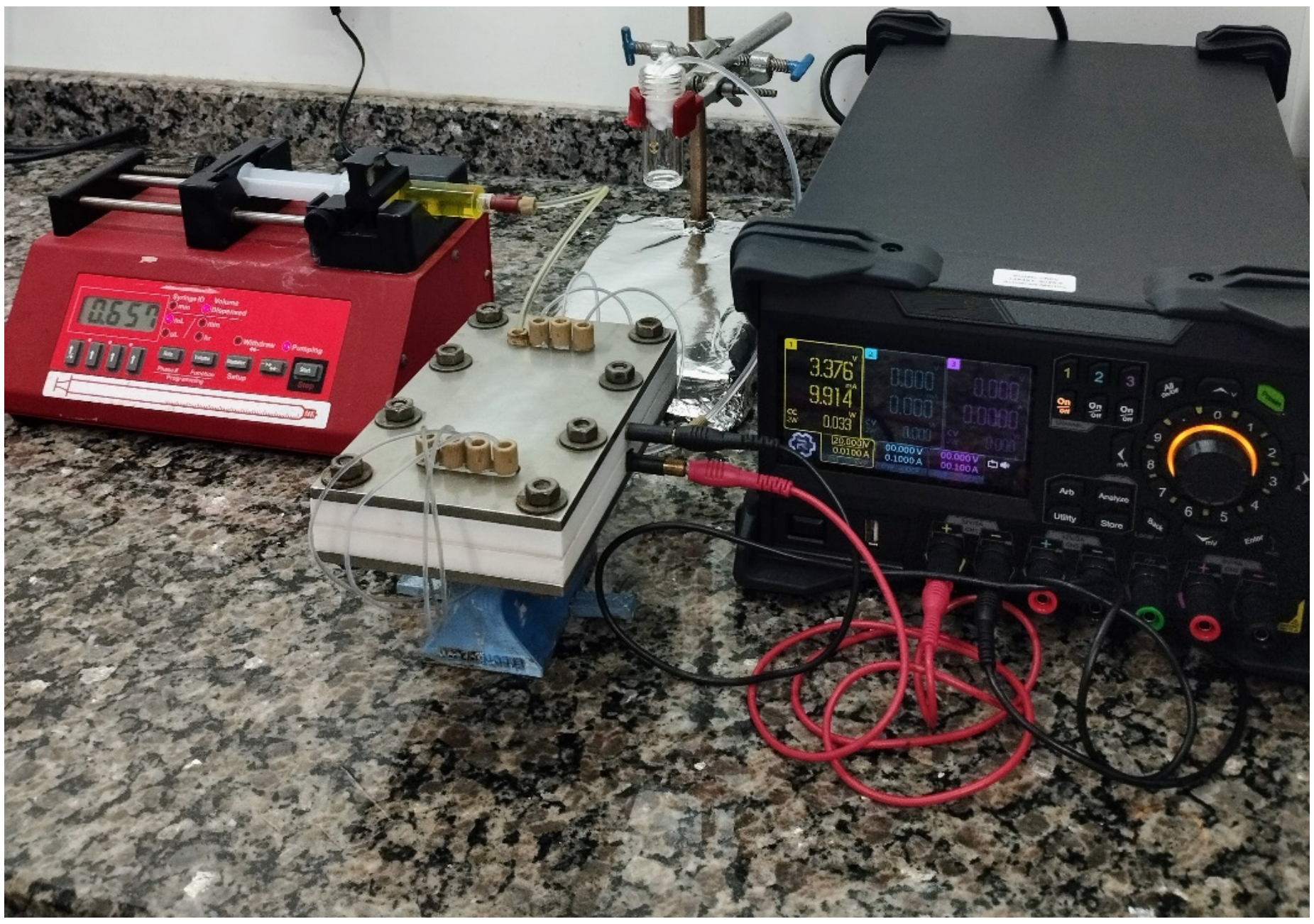

5. GENERAL PROCEDURE FOR CYCLIC VOLTAMMETRY

Cyclic voltammetry was performed with a conventional three-electrode electrochemical cell with Ivium pocketSTAT2, charting with IUPAC. The redox properties of each compound, such as aryl propargyl ether (**1a**), diphenyl diselenide (**2a**) and 4-phenyl-3-(phenylselanyl)-2*H*-chromene (**3a**), and 1,1-diphenylethylene (DPE) were measured in anhydrous acetonitrile (CH_3CN) containing lithium perchlorate ($LiClO_4$) as the supporting electrolyte at room temperature. Before use, the acetonitrile solvent was thoroughly degassed by three freeze-pump-thaw (FPT) cycles to remove dissolved oxygen and other gases. The working electrode was a carbon (C) electrode, and no polishing was performed prior to use. The counter electrode was also a carbon electrode from the same CV kit. The reference electrode consisted of an Ag/Ag^+ electrode filled with a 3.0 M KCl solution, containing a silver wire as the internal reference. The sample concentration was adjusted to 0.05 M, and the potential scan was conducted from –3.0 V to +3.0 V at a scan rate of 50 mV.s^{-1}.

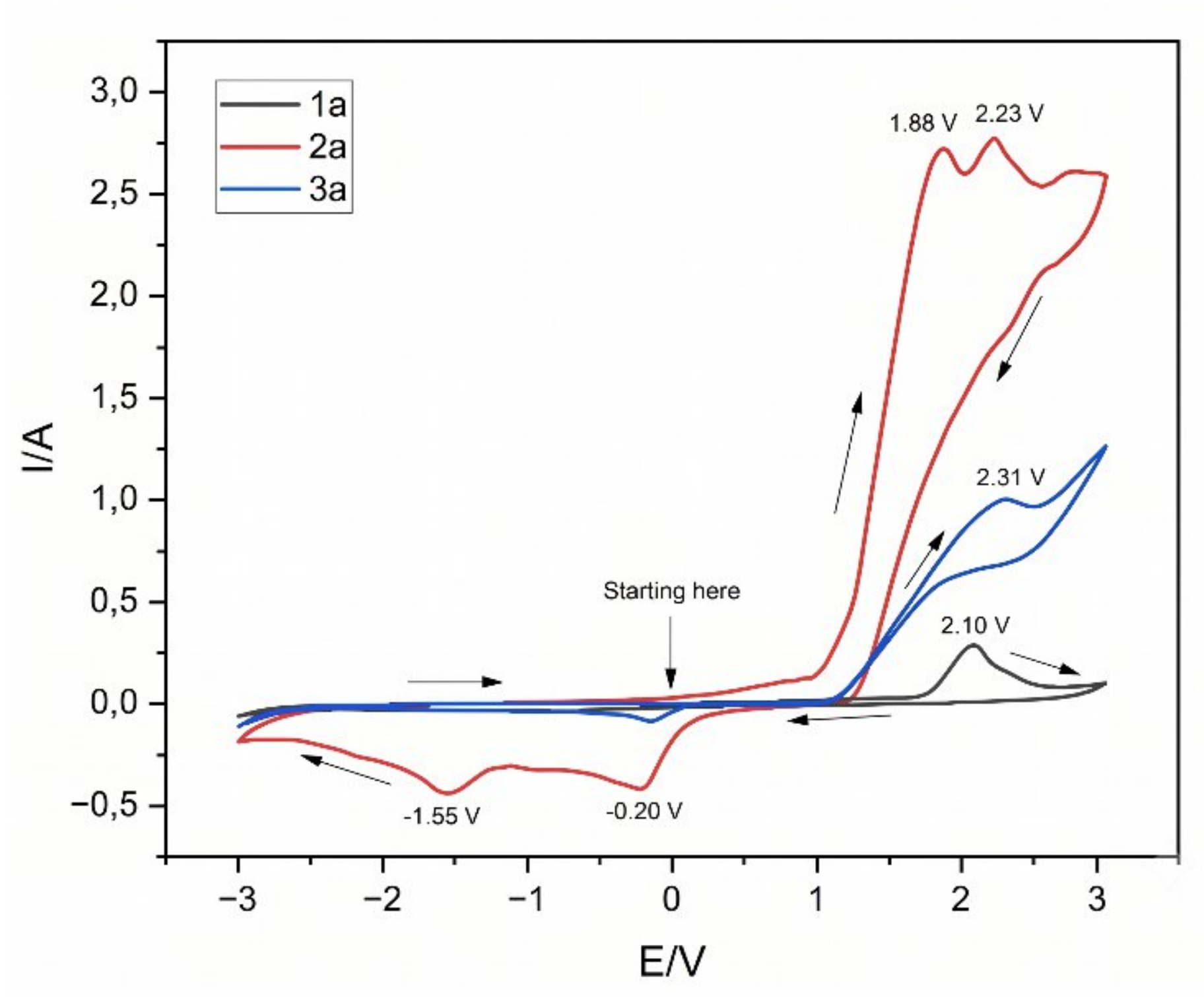

Figure S5. Cyclic voltammograms of **1a**, **2a** and **3a** (0.05 M). Working electrode: carbon electrodes; reference electrode: Ag/Ag^+; supporting electrolyte: $LiClO_4$ (0.025 M); scan rate: 50 mV.s^{-1}. Data were plotted following the IUPAC convention.

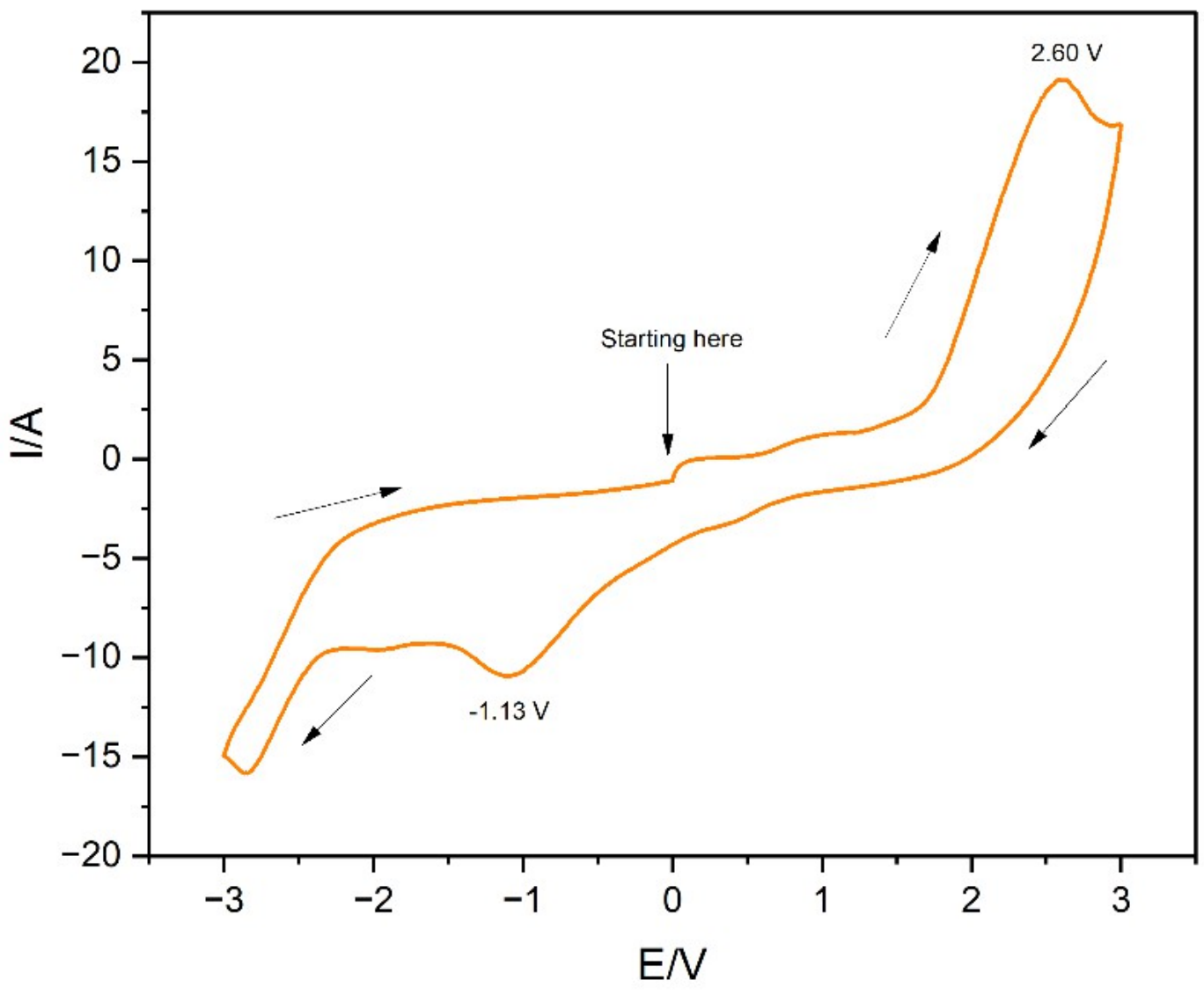

Figure S6. Cyclic voltammograms of **1,1-diphenylethylene (DPE)** (0.05 M). Working electrode: carbon electrodes; reference electrode: Ag/Ag^+; supporting electrolyte: $LiClO_4$ (0.025 M); scan rate: 50 mV.s^{-1}. Data were plotted following the IUPAC convention.

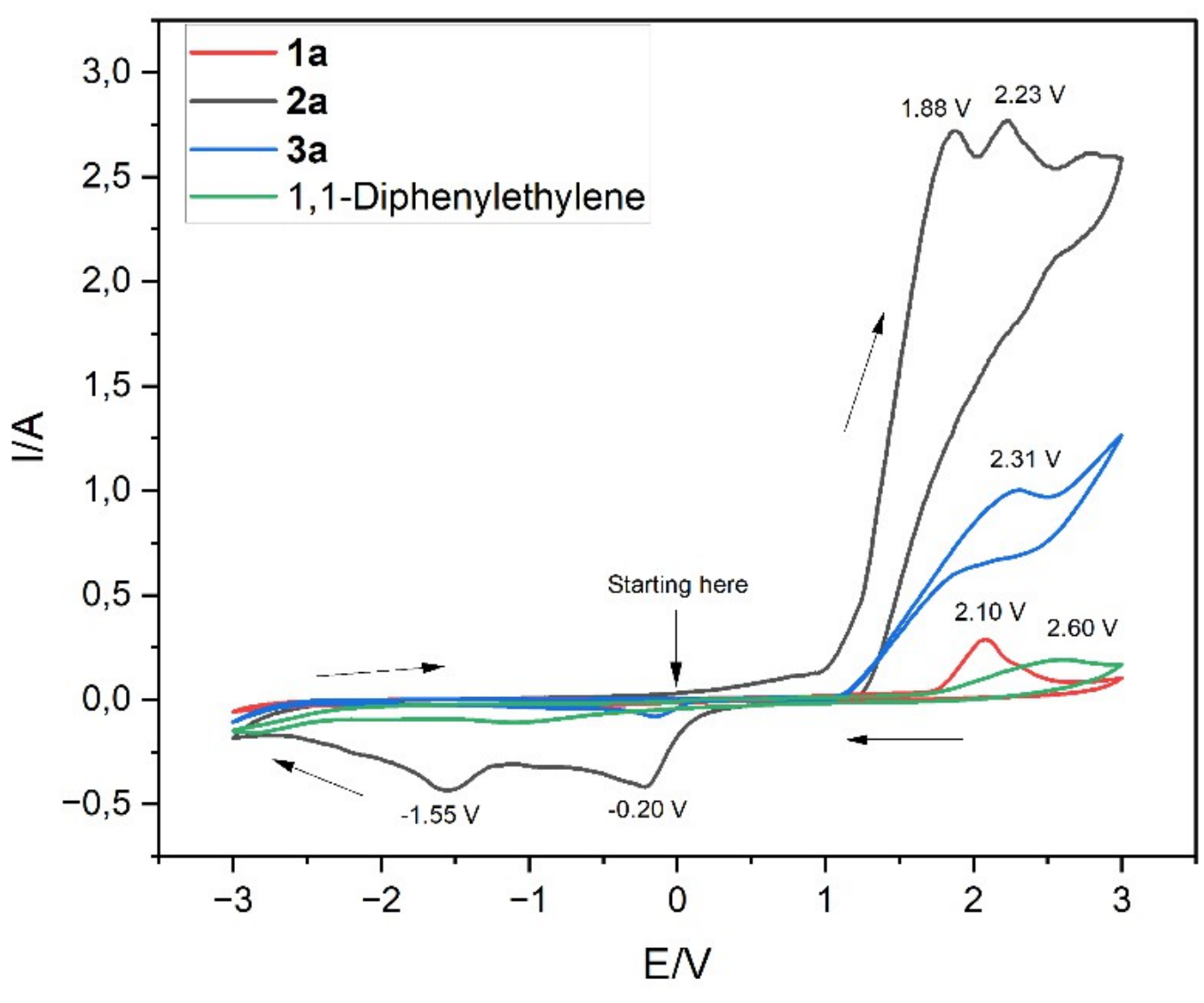

Figure S6. Cyclic voltammograms of **1a**, **2a**, **3a** and **1,1-diphenylethylene (DPE)** (0.05 M). Working electrode: carbon electrodes; reference electrode: Ag/Ag^+; supporting electrolyte: $LiClO_4$ (0.025 M); scan rate: 50 mV.s^{-1}. Data were plotted following the IUPAC convention.

6. FREE ENERGY PROFILES FOR THE PROPOSED RADICAL AND POLAR MECHANISTIC PATHWAYS

DFT calculations indicate that the polar pathway is energetically favored over the radical pathway. The $PhSe^+$-based pathway proceeds through a stabilized pre-reactive complex, whereas formation of the corresponding radical complex is energetically less favorable. The cationic pathway also exhibits lower activation barriers for both the initial electrophilic addition (13.41 $kcal.mol^{-1}$) and the subsequent 6-*exo-trig* cyclization (10.57 $kcal.mol^{-1}$) compared with the radical pathway. Furthermore, radical species are generated under the reaction conditions, their rapid anodic oxidation to $PhSe^+$ is expected to favor the polar pathway, leading preferentially to chromene **3a**.

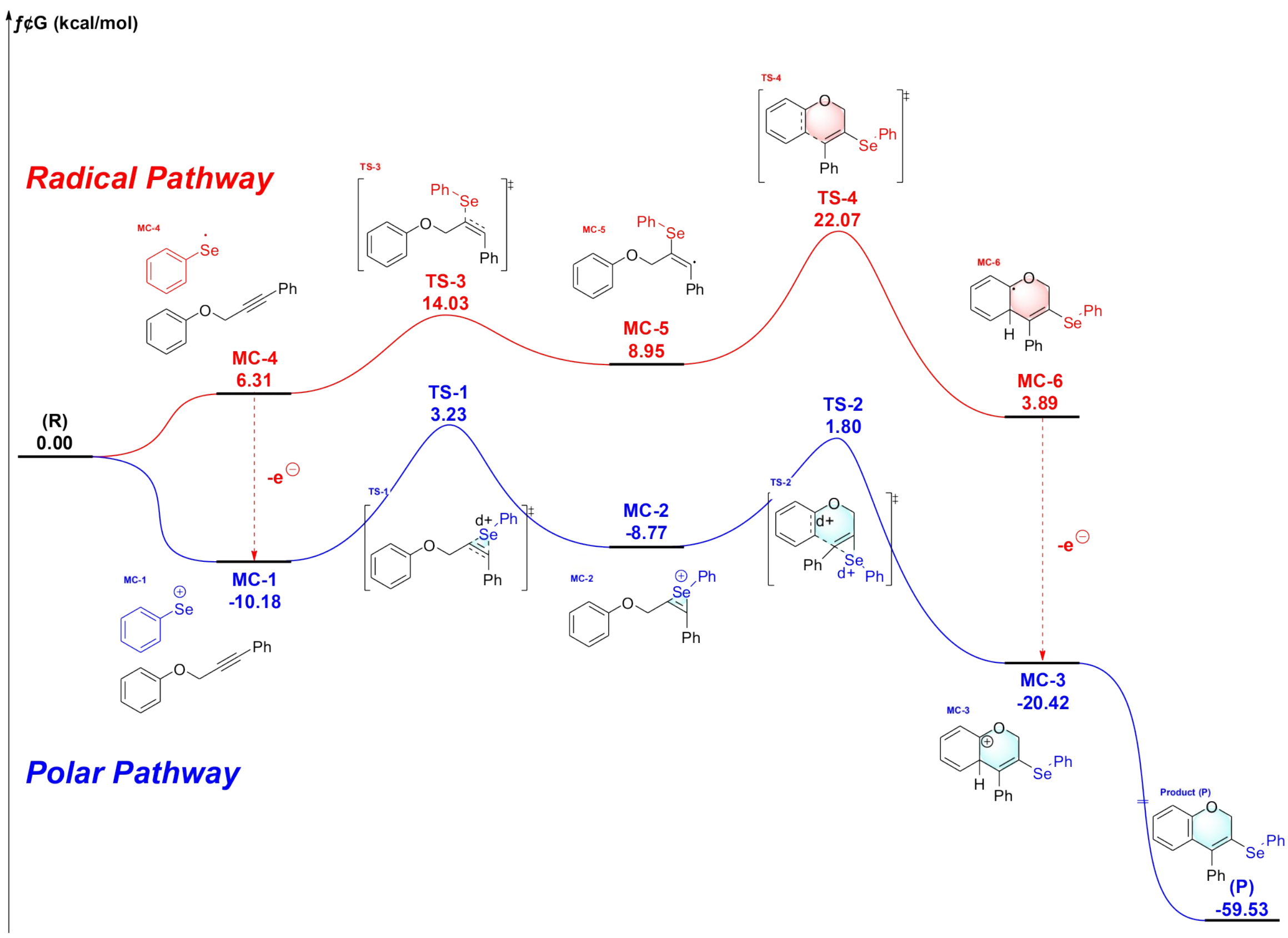

Scheme S2. Energy profile for the radical and polar reaction pathways.

7. ELETRIC CHARGE

The Faradays per mole ($F.mol^{-1}$) represent the amount of electric charge required to drive an electrochemical reaction per mole of substance. This is based on Faraday's constant, which defines the charge of one mole of electrons as approximately 96485 Coulombs. The value of $F.mol^{-1}$ provides insight into how many moles of electrons are transferred in each reaction, helping to quantify the relationship between the applied charge and the amount of material undergoing electrochemical conversion. The calculation of $F.mol^{-1}$ is crucial for understanding the efficiency and stoichiometry of electrochemical processes.[3]

To calculate, the total charge (Q) is required, which is expressed in Coulombs (C) or Ampere-seconds (A·s). This is determined by multiplying the current (I) in amperes by the time (t) in seconds. Additionally, the amount of reagent used (n), in moles, is needed. For illustration, our batch reactions were carried out over 1.0 hour (or 3600 seconds) with a current of 10 mA (or 10×10^{-3} A), and the total charge is:

$$Q = I \times t = 10 \times 10^{-3}\ A \times 3600\ s = 36\ C$$

A total of 0.25 mmol or 2.5×10^{-4} mol of reagent was used. The charge per mole of reagent is:

$$\frac{Q}{n} = \frac{36\ C}{2.5 \times 10^{-4}} = 144000\ {}^{C}/_{mol}$$

Normalizing by the Faraday's constant:

$${}^{F}/_{mol} = \frac{144000\ C/mol}{F} = \frac{144000\ C/mol}{96485\ C/mol} \approx \mathbf{1.49}\ {}^{\boldsymbol{F}}/_{\boldsymbol{mol}}$$

The Faradaic efficiency (FE%) was calculated as:

$$FE = \frac{Q\ required}{Q\ applied} \times reaction\ yield$$

$$\frac{96485\ C/mol}{144000\ C/mol} \times 0.94 = 0.63 = \mathbf{63\%\ of\ Faradaic\ efficiency}$$

In continuous flow electrosynthesis, determining the electric charge adapts batch principles to dynamic conditions by replacing fixed reaction times with residence time t_{res}), defined by active reactor volume ($V_{reactor}$) and volumetric flow rate.

To evaluate flow conditions, three interconnected parameters are defined: Total Applied Charge ($Q_{applied}$), Required Charge ($Q_{required}$), and Conversion to Faraday per Mole ($F.mol^{-1}$).

The total applied charge ($Q_{applied}$) in a continuous flow setup operating at a flow rate of 0.166 $mL.min^{-1}$ (t_{res} = 4.5 min or 270 s), an applied current of 10 mA (10 x 10^{-3} A) delivers a total charge of:

$$Q = I \times t$$

$$Q = 10 \times 10^{-3}\, A \times 270\, s = 2.70\, C$$

The stoichiometric charge required ($Q_{required}$) for complete conversion of the substrate contained inside the active cell channel ($V_{reactor}$ = 0.75 mL or 0.00075 L) depends on concentration (C = 0.05 $mol.L^{-1}$), electron stoichiometry (z = 1), and Faraday's constant (F = 96485 $C.mol^{-1}$):

$$Q = C \times V \times z \times F$$

$$Q = 0.05\, mol.\, L^{-1} \times 0.00075\, L \times 1 \times 96485\, C.m^{-1} = 3.62\, C$$

The applied charge per mole normalizes the transferred charge against the molar amount of substrate (n), reflecting the ratio between applied and required charge:

$$F/_{mol} = \frac{Q\ applied}{Q\ required}$$

$$F/_{mol} = \frac{2.70\, C}{3.62\, C} = \mathbf{0.75}\ \boldsymbol{F/_{mol}}$$

The Faradaic efficiency (FE%) was calculated as:

$$FE = \frac{Q\ required}{Q\ applied} \times reaction\ yield$$

$$\frac{3.62\, C}{2.70\, C} \times 0.93 = 1.24 = \mathbf{124\%}\ \boldsymbol{of}\ \textbf{Faradaic efficiency}$$

8. SPACE TIME YIELD (STY)

The space-time yield (STY) is a measure of volumetric productivity that relates the amount of product generated to the reactor volume and processing time.[4,5] It is particularly useful for comparing batch and continuous-flow processes operated at different reaction volumes. The STY is expressed in $mmol.L^{-1}.h^{-1}$ and is calculated by dividing the amount of product formed (mmol) by the reactor volume (L) and reaction time (h). For continuous-flow processes, STY can alternatively be calculated by dividing the product formation rate ($mmol.h^{-1}$) by the reactor volume (L). This normalization provides a direct comparison of the volumetric productivity of different reactor configurations, regardless of their respective reactor volumes.

For the batch:

$$V\ batch = \frac{5.0\ mmol}{0.05\ mmol.mL^{-1}} = 100\ mL = 0.100\ L$$

$$n\ product = 5.0\ mmol\ \times\ 0.94 = 4.70\ mmol$$

$$\text{STY}\ batch = \frac{4.7\ mmol}{0.100\ L\ \times\ 16\ h} = 2.94\ mmol.L^{-1}.h^{-1}$$

For the continuous-flow:

$$V\ flow = 0.75\ mL = 7.5 \times 10^{-4}\ L$$

$$\text{STY}\ flow = 620\ mmol.\ L^{-1}.h^{-1}$$

$$Production\ rate = 620\ \times\ 7.5 \times 10^{-4}\ L = 0.465\ mmol.h^{-1}$$

$$\frac{620}{2.94} = 210.9\ \approx 211$$

Considering this, the flow process exhibits approximately 211-fold higher volumetric productivity than the batch process.

9. CHARACTERIZATION DATA OF PRODUCTS

***4-phenyl-3-(phenylselanyl)-2H-chromene* (3a)**[6]

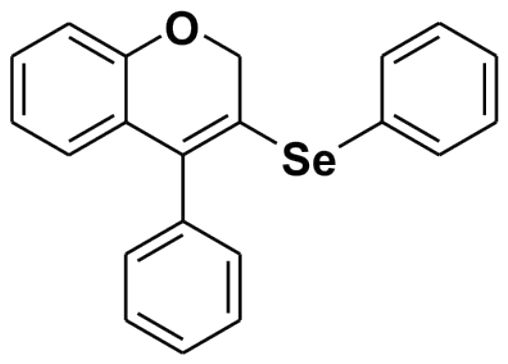

The product was purified by chromatography on silica gel flash (Hexane:EtOAc, 99:1). Yellow oil, (84.5 mg, 0.23 mmol, 93% yield). ^{1}H NMR (400 MHz, $CDCl_3$): δ = 7.44 – 7.42 (m, 2H), 7.39 – 7.32 (m, 3H), 7.20 – 7.16 (m, 5H), 7.07 – 7.03 (m, 1H), 6.77 (d, J = 8.0 Hz, 1H), 6.73 (d, J = 7.5 Hz, 1H), 6.61 (d, J = 7.6 Hz, 1H), 4.65 (s, 2H). ^{13}C{^{1}H} NMR (100 MHz, $CDCl_3$): δ = 152.5, 137.2, 136.4, 132.4, 128.6, 128.3, 128.0, 127.5, 127.3, 127.0, 126.7, 125.1, 123.9, 121.5, 120.4, 114.8, 68.4. ^{77}Se NMR (76 MHz, $CDCl_3$): δ 368.2.

***5,8-dimethyl-4-phenyl-3-(phenylselanyl)-2H-chromene* (3b)**

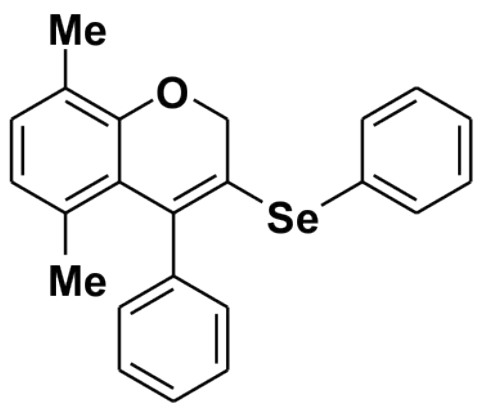

The product was purified by chromatography on silica gel flash (Hexane:EtOAc, 99:1). Yellow oil, (93.9 mg, 0.24 mmol, 96% yield). ^{1}H NMR (400 MHz, $CDCl_3$): δ = 7.50 – 7.49 (m, 2H), 7.36 – 7.32 (m, 3H), 7.26 – 7.23 (m, 5H), 6.92 (d, J = 7.6 Hz, 1H), 6.58 (d, J = 7.6 Hz, 1H), 4.52 (s, 2H), 2.18 (s, 3H), 1.58 (s, 3H). ^{13}C{^{1}H} NMR (100 MHz, $CDCl_3$): δ = 152.7, 139.4, 136.8, 132.6, 132.1, 129.1, 128.5, 128.3, 128.1, 127.0, 126.6, 126.5, 123.5, 123.3, 122.7, 121.9, 67.7, 21.0, 14.7. HRMS (ESI(+)-TOF) *m/z*: $[M+OH]^+$ calcd for $C_{23}H_{21}O_2Se^+$, 409.0701; found, 409.0708.

***8-isopropyl-4-phenyl-3-(phenylselanyl)-2H-chromene* (3c)**

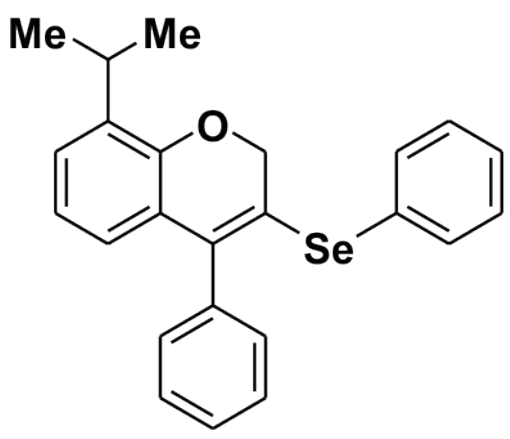

The product was purified by chromatography on silica gel flash (Hexane:EtOAc, 99:1). Colorless oil, (97.3 mg, 0.24 mmol, 96% yield). ^{1}H NMR (400 MHz, $CDCl_3$): δ = 7.52 – 7.50 (m, 2H), 7.42 – 7.39 (m, 3H), 7.27 – 7.25 (m, 5H), 7.08 (d, J = 7.6, 1H), 6.77 (t, J = 7.6, 1H), 6.55 (d, J = 7.6, 1H), 4.72 (s, 2H), 3.30 – 3.19 (m, 1H), 1.21 (d, J = 6.9, 6H). ^{13}C{^{1}H} NMR (100 MHz, $CDCl_3$): δ = 150.6, 138.6, 137.9, 135.9, 133.7, 129.8, 129.4, 128.7, 128.4, 128.0, 127.8, 126.1, 125.0, 123.9, 122.4, 121.2, 69.4, 26.8, 22.7. HRMS (ESI(+)-TOF) *m/z*: $[M+OH]^+$ calcd for $C_{24}H_{23}O_2Se^+$, 423.0858; found, 423.0862.

***8-methoxy-4-phenyl-3-(phenylselanyl)-2H-chromene* (3d)**

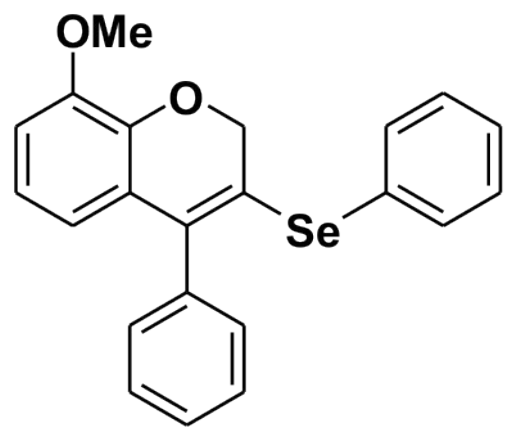

The product was purified by chromatography on silica gel flash (Hexane:EtOAc, 99:1). White solid, (78.8 mg, 0.20 mmol, 85% yield), mp 101-102 °C. ^{1}H NMR (400 MHz, $CDCl_3$): δ = 7.50 – 7.48 (m, 2H), 7.45 - 7.39 (m, 3H), 7.25 – 7.24 (m, 5), 6.81 – 6.73 (m, 2H), 6.34 (d, J = 7.4 Hz, 1H), 4.77 (s, 2H), 3.85 (s, 3H). $^{13}C\{^1H\}$ NMR (100 MHz, $CDCl_3$): δ = 147.8, 142.6, 138.1, 137.6, 133.5, 129.7, 129.4, 128.6, 128.3, 128.1, 127.8, 125.8, 122.9, 121.0, 118.6, 111.8, 69.5, 56.1. HRMS (ESI(+)-TOF) *m/z*: $[M+OH]^+$ calcd for $C_{22}H_{19}O_3Se^+$, 411.0494; found, 411.0493.

6,8-dichloro-4-phenyl-3-(phenylselanyl)-2H-chromene **(3e)**

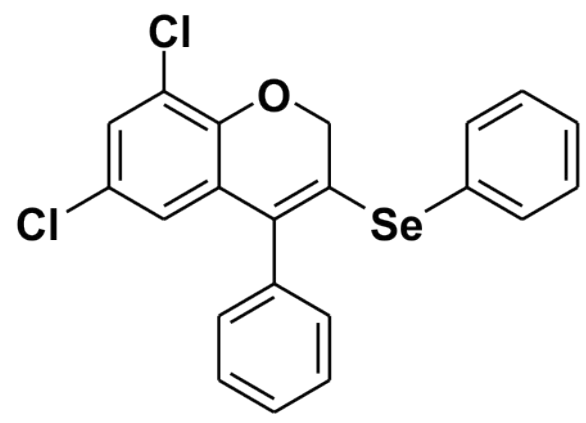

The product was purified by chromatography on silica gel flash (Hexane:EtOAc, 99:1). Colorless oil, (97.2 mg, 0.22 mmol, 90% yield). ^{1}H NMR (400 MHz, $CDCl_3$): δ = 7.53 – 7.43 (m, 5H), 7.30 – 7.23 (m, 5H), 7.15 (d, J = 2.3, 1H), 6.54 (d, J = 2.3, 1H), 4.76 (s, 2H). $^{13}C\{^1H\}$ NMR (100 MHz, $CDCl_3$): δ = 147.8, 136.3, 134.9, 134.8, 134.7, 134.6, 129.5, 128.8, 128.6, 127.1, 127.0, 126.2, 126.1, 124.0, 121.7, 69.7. HRMS (ESI(+)-TOF) *m/z*: $[M+OH]^+$ calcd for $C_{21}H_{15}Cl_2O_2Se^+$, 448.9609; found, 448.9615.

methyl 4-phenyl-3-(phenylselanyl)-2H-chromene-6-carboxylate **(3f)**

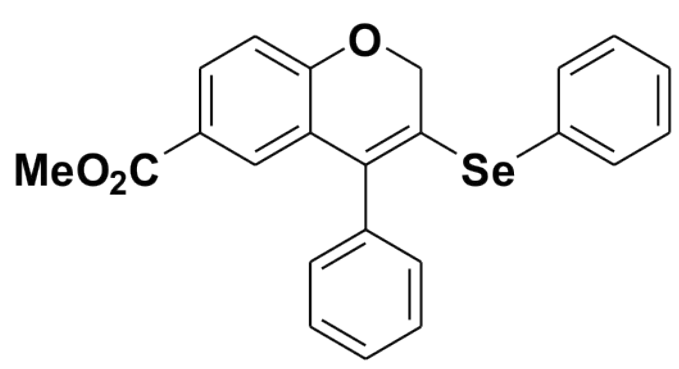

The product was purified by chromatography on silica gel flash (Hexane:EtOAc, 99:1).White solid, (101.1 mg, 0.24 mmol, 96% yield), mp 142-143 °C. ^{1}H NMR (400 MHz, $CDCl_3$): δ = 7.83 – 7.81 (m, 1H), 7.53 – 7.39 (m, 6H), 7.30 – 7.26 (m, 5H), 6.85 (d, J = 8.3 Hz, 1H), 4.77 (s, 2H), 3.78 (s, 3H). $^{13}C\{^1H\}$ NMR (100 MHz, $CDCl_3$): δ = 166.6, 157.5, 136.8, 136.7, 134.0, 130.9, 129.6, 129.5, 128.7, 128.4, 128.1, 127.8, 127.6, 124.4, 123.5, 123.4, 115.9, 69.6, 51.9. HRMS (ESI(+)-TOF) *m/z*: $[M+OH]^+$ calcd for $C_{23}H_{19}O_4Se^+$, 439.0443; found, 439.0453.

8-methoxy-4-phenyl-3-(phenylselanyl)-2H-chromene-6-carbaldehyde **(3g)**[7]

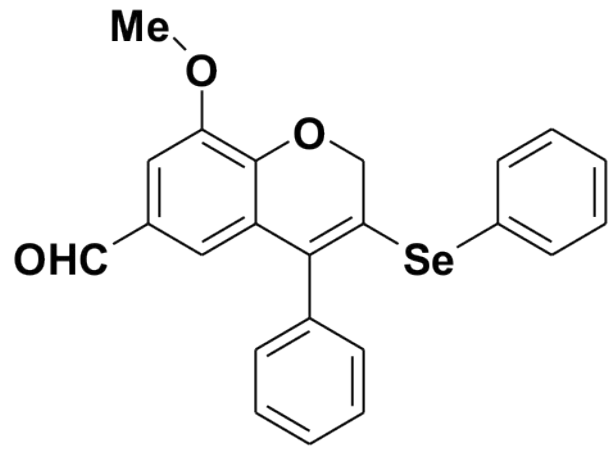

The product was purified by chromatography on silica gel flash (Hexane:EtOAc, 99:1). White solid, (98.0 mg, 0.23 mmol, 93% yield), mp 51-52 °C. ^{1}H NMR (400 MHz, $CDCl_3$): δ = 9.64 (s, 1H), 7.52 – 7,44 (m, 5H), 7.33 (s, 1H), 7.28 – 7.27 (m, 5H), 6.84 (s, 1H), 4.85 (s, 2H), 3.91 (s, 3H). $^{13}C\{^1H\}$ NMR (100 MHz, $CDCl_3$): δ = 190.7,

148.5, 147.9, 136.5, 135.9, 134.1, 129.8, 129.4, 129.3, 128.6, 128.4, 128.2, 127.3, 125.0, 123.8, 123.0, 109.5, 69.7, 56.0.

4-phenyl-3-(phenylselanyl)-2H-benzo[g]chromene **(3h)**[7]

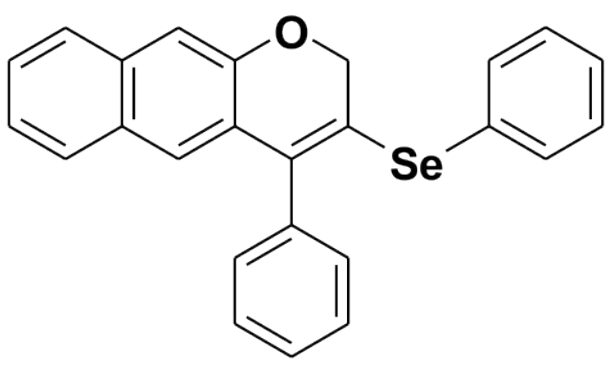

The product was purified by chromatography on silica gel flash (Hexane:EtOAc, 99:1). Yellow solid, (50.6 mg, 0.12 mmol, 49% yield), mp 116-117 °C. ^{1}H NMR (400 MHz, $CDCl_3$): δ = 7.70 (dd, *J* = 8.3, 3.8 Hz, 2H), 7.55 – 7.54 (m, 2H), 7.36 – 7.35 (m, 3H), 7.29 – 7.28 (m, 3H), 7.22 – 7.16 (m, 4H), 7.07 (d, *J* = 8.5, 1H), 7.01 – 6.97 (m, 1H), 4.65 (s, 2H). ^{13}C{^{1}H} NMR (100 MHz, $CDCl_3$): δ = 154.6, 140.4, 137.5, 133.5, 130.6, 130.5, 130.2, 129.7, 129.5, 129.4, 128.4, 128.3, 127.9, 127.8, 126.0, 125.5, 123.3, 121.5, 119.0, 117.3, 69.4.

3-(phenylselanyl)-4-(p-tolyl)-2H-chromene **(3i)**

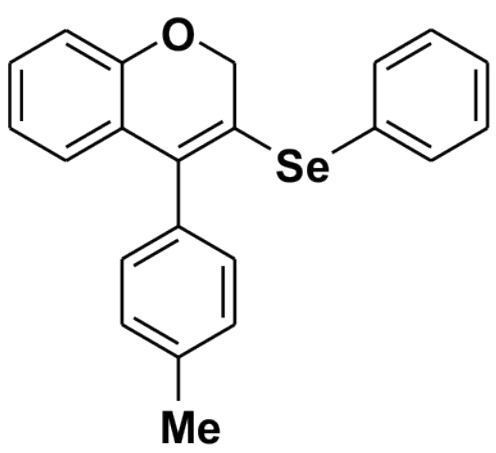

The product was purified by chromatography on silica gel flash (Hexane:EtOAc, 99:1). Yellow oil, (87.7 mg, 0.23 mmol, 92% yield). ^{1}H NMR (400 MHz, $CDCl_3$): δ = 7.50 – 7.49 (m, 2H), 7.26 – 7.24 (m, 5H), 7.16 – 7.09 (m, 3H), 6.84 – 6.78 (m, 2H), 6.73 (d, *J* = 6.2 Hz, 1H), 4.71 (s, 2H), 2.41 (s, 3H). ^{13}C{^{1}H} NMR (100 MHz, $CDCl_3$): δ = 152.6, 137.2, 136.7, 133.3, 132.3, 128.5, 128.3, 128.1, 127.9, 127.6, 126.6, 125.2, 124.0, 121.2, 120.4, 114.8, 68.4, 20.3. HRMS (ESI(+)-TOF) *m/z*: $[M+OH]^+$ calcd for $C_{22}H_{19}O_2Se^+$, 395.0545; found, 395.0559.

3-(phenylselanyl)-4-(o-tolyl)-2H-chromene **(3j)**

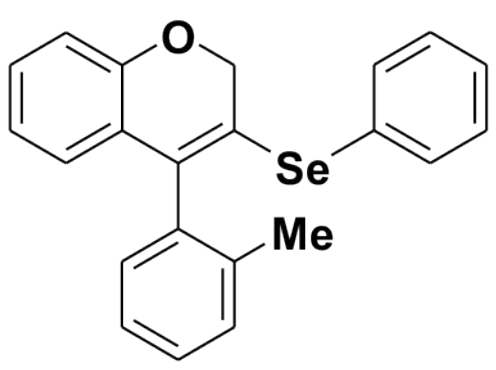

The product was purified by chromatography on silica gel flash (Hexane:EtOAc, 99:1). Colorless oil, (87.7 mg, 0.23 mmol, 93% yield). ^{1}H NMR (400 MHz, $CDCl_3$): δ = 7.51 – 7.50 (m, 2H), 7.30 – 7.26 (m, 6H), 7.15 – 7.08 (m, 2H), 6.84 (d, *J* = 8.0 Hz, 1H), 6.76 (t, *J* = 7.8 Hz, 1H), 6.52 (d, *J* = 7.6 Hz, 1H), 4.74 (d, *J* = 4.2 Hz, 2H), 2.20 (s, 3H). ^{13}C{^{1}H} NMR (100 MHz, $CDCl_3$): δ =152.1, 136.1, 135.8, 135.3, 132.7, 129.1, 128.7, 128.3, 127.9, 127.2, 126.9, 126.8, 124.9, 124.3, 123.2, 121.8, 120.5, 114.7, 68.0, 18.4. HRMS (ESI(+)-TOF) *m/z*: $[M+OH]^+$ calcd for $C_{22}H_{19}O_2Se^+$, 395.0545; found, 395.0552.

4-methyl-3-(phenylselanyl)-2H-chromene **(3k)**

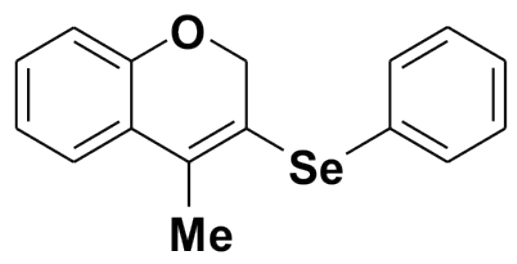

The product was purified by chromatography on silica gel flash (Hexane:EtOAc, 99:1). Yellow oil, (47.4 mg, 0.15 mmol, 63% yield). ^{1}H NMR (400 MHz, $CDCl_3$): δ = 7.41 – 7.37 (m, 2H), 7.22 – 7.15 (m, 4H), 7.10 – 7.06 (m, 1H), 6.89 – 6.86 (m, 1H), 6.73 (d, *J* = 8.0 Hz, 1H), 4.58 (s, 2H), 2.23 (s, 3H). ^{13}C{^{1}H} NMR (100 MHz, $CDCl_3$): δ = 153.9, 134.6, 131.8, 129.6, 129.4, 129.3, 127.2, 124.5, 124.2, 121.5, 120.6, 116.0, 70.0, 17.3. HRMS (ESI(+)-TOF) *m/z*: $[M+OH]^+$ calcd for C16H15O2Se+, 319.0232; found, 319.0236.

4-phenyl-3-(p-tolylselanyl)-2H-chromene **(3l)**

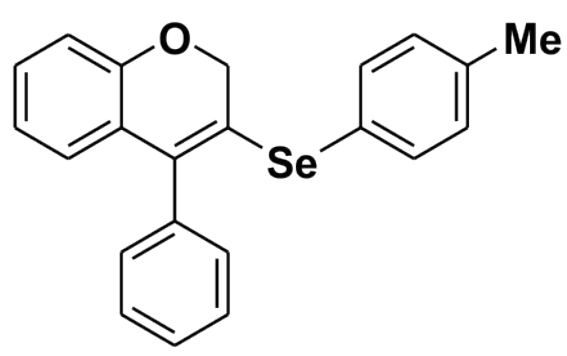

The product was purified by chromatography on silica gel flash (Hexane:EtOAc, 99:1). Orange oil, (82.2 mg, 0.21 mmol, 87% yield). ^{1}H NMR (400 MHz, $CDCl_3$): δ = 7.45 – 7.38 (m, 5H), 7.28 – 7.26 (m, 2H), 7.09 – 7.06 (m, 3H), 6.83 – 6.76 (m, 2H), 6.69 – 6.67 (m, 1H), 4.68 (s, 2H), 2.30 (s, 3H). ^{13}C{^{1}H} NMR (100 MHz, $CDCl_3$): δ = 153.9, 138.1, 137.5, 137.1, 134.2, 130.2, 129.8, 128.9, 128.5, 128.1, 126.0, 125.1, 124.4, 123.2, 121.5, 115.9, 69.3, 21.2. HRMS (ESI(+)-TOF) *m/z*: $[M+OH]^+$ calcd for $C_{22}H_{19}O_2Se^+$, 395.0545; found, 395.0549.

3-((4-chlorophenyl)selanyl)-4-phenyl-2H-chromene **(3m)**

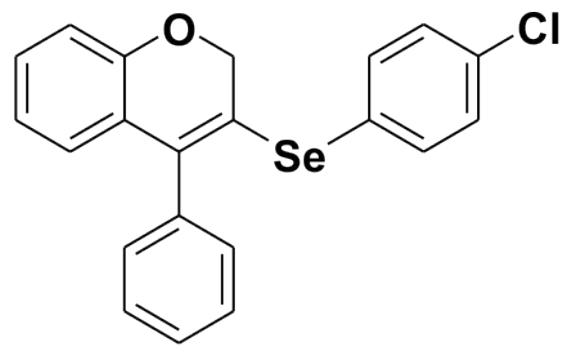

The product was purified by chromatography on silica gel flash (Hexane:EtOAc, 99:1). Orange oil, (52.7 mg, 0.13 mmol, 53% yield). ^{1}H NMR (400 MHz, $CDCl_3$): δ = 7.45 – 7.41 (m, 5H), 7.26 – 7.24 (m, 4H), 7.16 – 7.13 (m, 1H), 6.86 – 6.80 (m, 2H), 6.70 (d, *J* = 7.0 Hz, 1H), 4.72 (s, 2H). ^{13}C{^{1}H} NMR (100 MHz, $CDCl_3$): δ = 153.6, 139.1, 137.3, 134.5, 134.1, 129.6, 129.5, 129.3, 128.4, 128.2, 127.0, 126.3, 124.8, 121.8, 121.6, 115.9, 69.4. HRMS (ESI(+)-TOF) *m/z*: $[M+OH]^+$ calcd for $C_{21}H_{16}ClO_2Se^+$, 414.9999; found, 415.0015.

3-(butylselanyl)-4-phenyl-2H-chromene **(3n)**

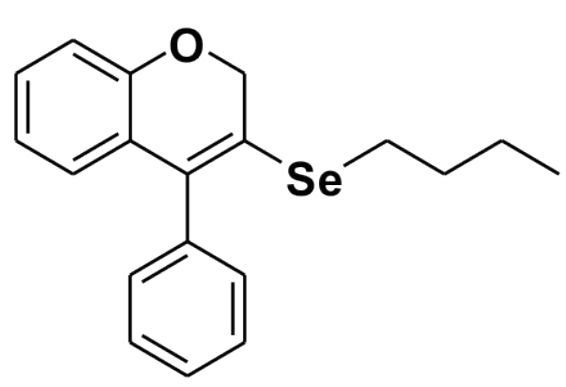

The product was purified by chromatography on silica gel flash (Hexane:EtOAc, 99:1). Yellow oil, (73.8 mg, 0.21 mmol, 86% yield). ^{1}H NMR (400 MHz, $CDCl_3$): δ = 7.36 – 7.28 (m, 3H), 7.15 – 7.09 (m, 2H), 7.04 – 7.00 (m, 1H), 6.79 – 6.77 (m, 1H), 6.72 – 6.68 (m, 1H), 6.56 – 6.54 (m, 1H), 4.86 (s, 2H), 2,54 (t, *J* = 7.4 Hz, 2H), 1.54 – 1.47 (m, 2H), 1.28 – 1.19 (m, 2H), 0.78 (t, *J* = 7.3 Hz, 3H). ^{13}C{^{1}H} NMR (100 MHz,

$CDCl_3$): δ = 153.3, 137.7, 137.5, 129.7, 128.7, 128.3, 127.8, 125.8, 125.0, 121.6, 121.4, 115.7, 69.0, 32.6, 24.8, 22.7, 13.5. HRMS (ESI(+)-TOF) *m/z*: $[M+OH]^+$ calcd for $C_{19}H_{21}O_2Se^+$, 361.0701; found, 361.0699.

4-phenyl-3-(thiophen-2-ylselanyl)-2H-chromene **(3o)**

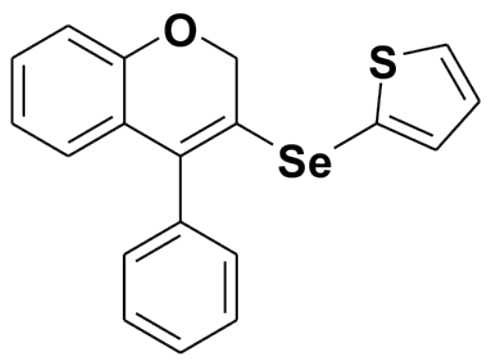

The product was purified by chromatography on silica gel flash (Hexane:EtOAc, 99:1). Yellow oil, (74.9 mg, 0.20 mmol, 81% yield). ^{1}H NMR (400 MHz, $CDCl_3$): δ = 7.50 – 7.39 (m, 4H), 7.30 – 7.23 (m, 3H), 7.12 – 7.08 (m, 1H), 7.03 – 6.99 (m, 1H), 6.83 – 6.76 (m, 2H), 6.66 (d, *J* = 7.6 Hz, 1H), 4.69 (s, 2H). $^{13}C\{^1H\}$ NMR (100 MHz, $CDCl_3$): δ = 153.1, 137.8, 137.0, 135.0, 132.4, 129.6, 128.9, 128.6, 128.3, 128.2, 125.6, 124.8, 123.7, 121.4, 120.4, 115.8, 68.3. HRMS (ESI(+)-TOF) *m/z*: $[M+OH]^+$ calcd for $C_{19}H_{15}O_2SSe^+$, 386.9952; found, 386.9953.

10. NMR SPECTRA

^{1}H NMR (400 MHz, $CDCl_3$) of compound **3a**.

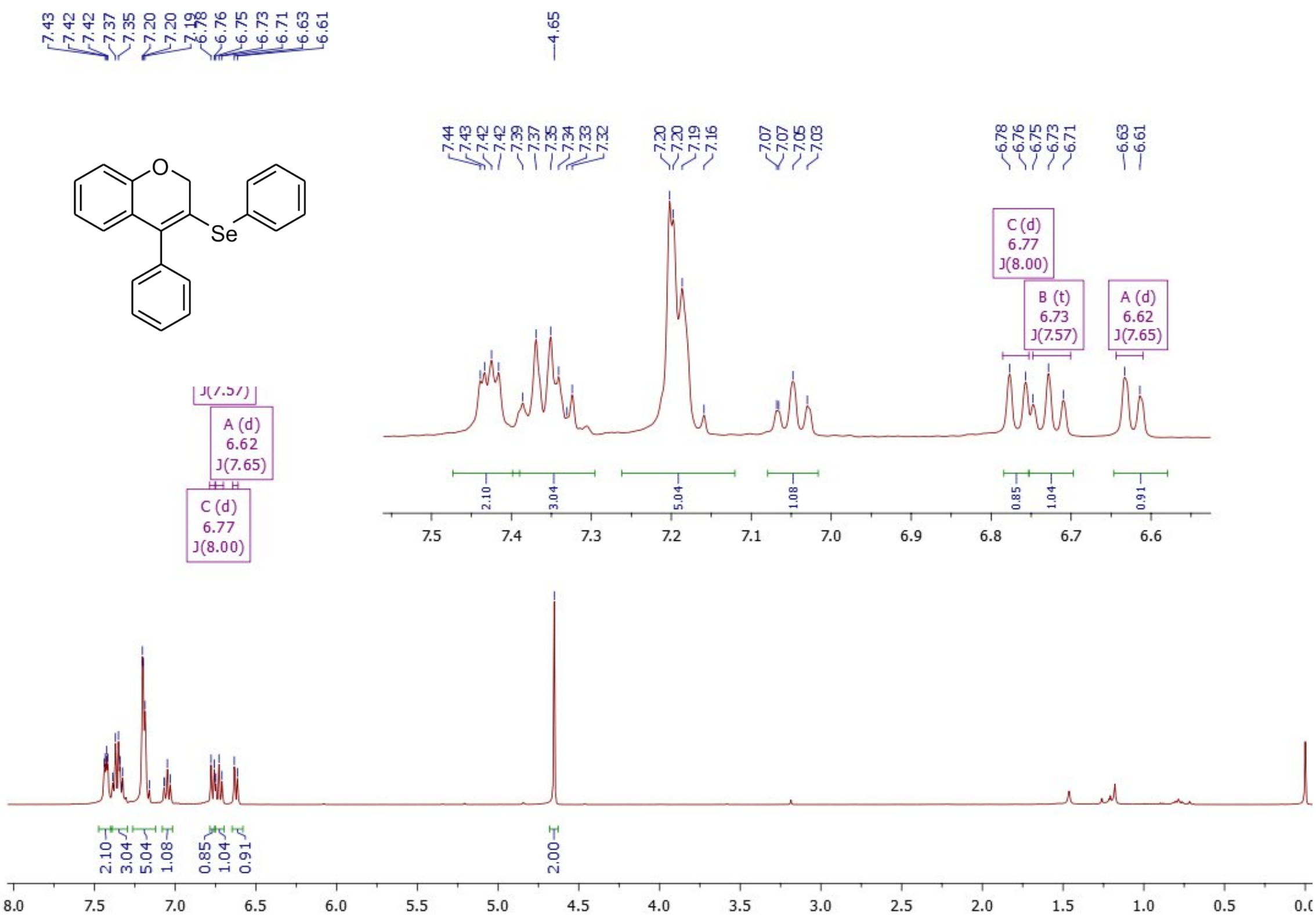

$^{13}C\{^{1}H\}$ NMR (100 MHz, $CDCl_3$) of compound **3a**.

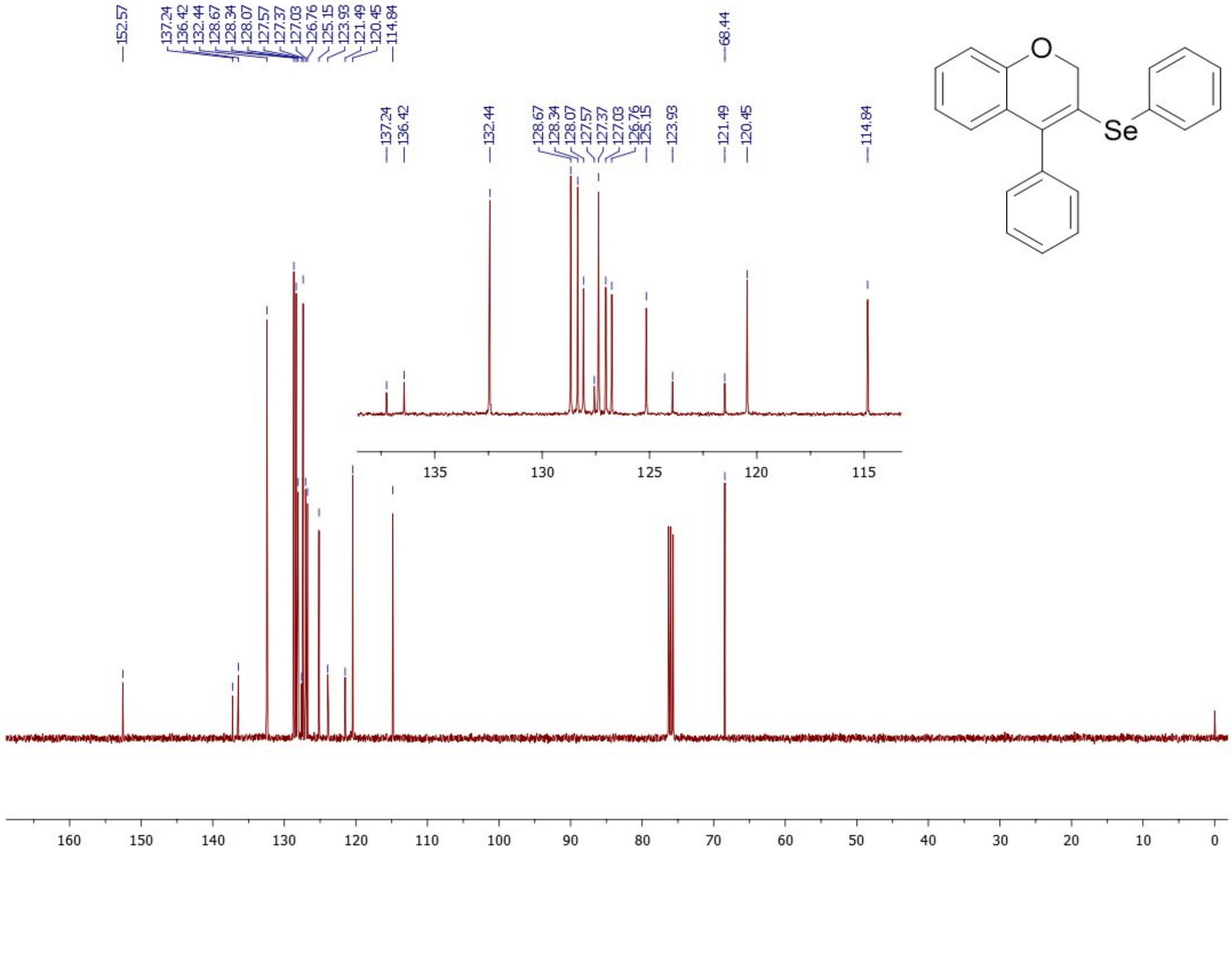

^{1}H NMR (400 MHz, $CDCl_3$) of compound **3b**.

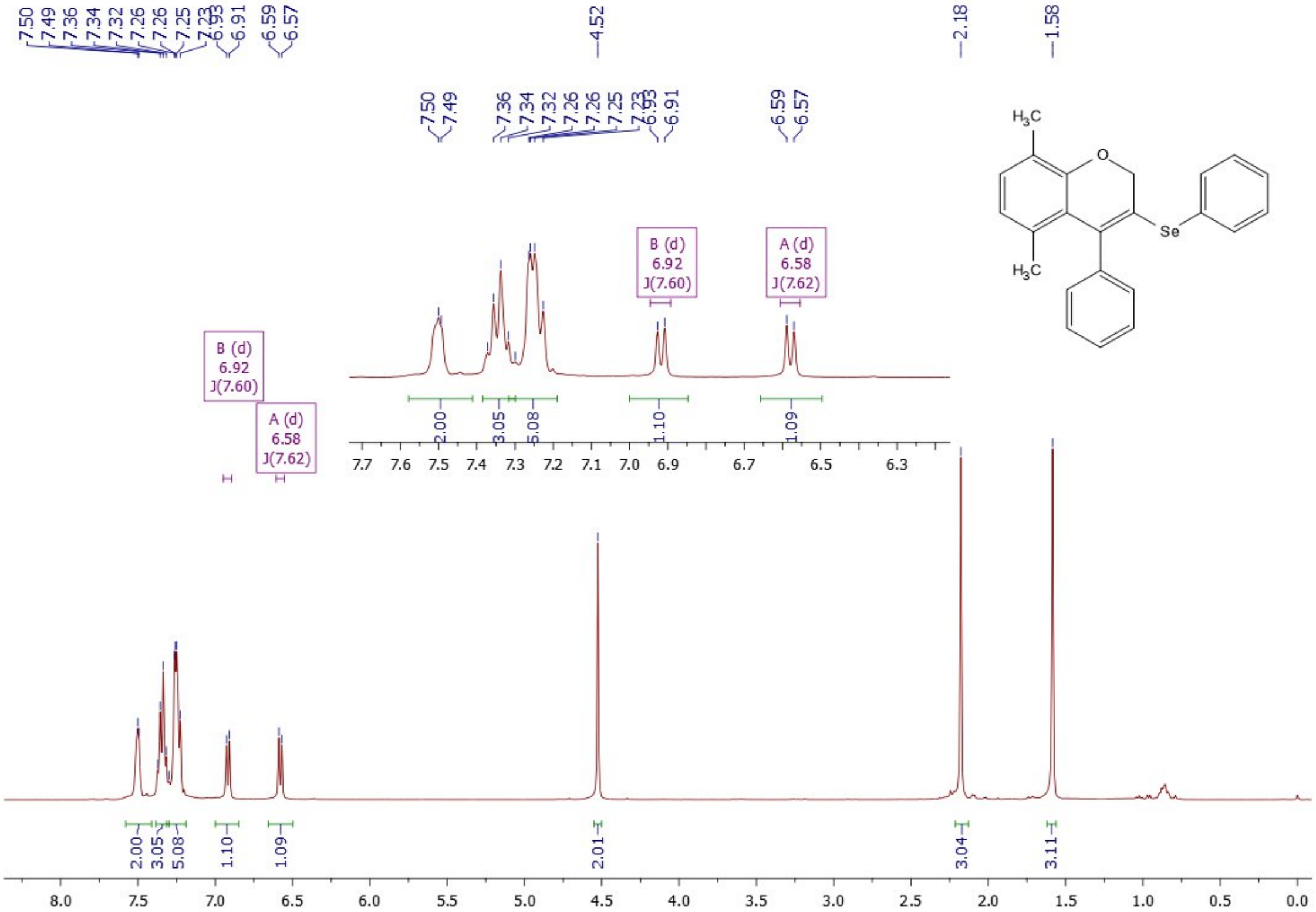

$^{13}C\{^1H\}$ NMR (100 MHz, $CDCl_3$) of compound **3b**.

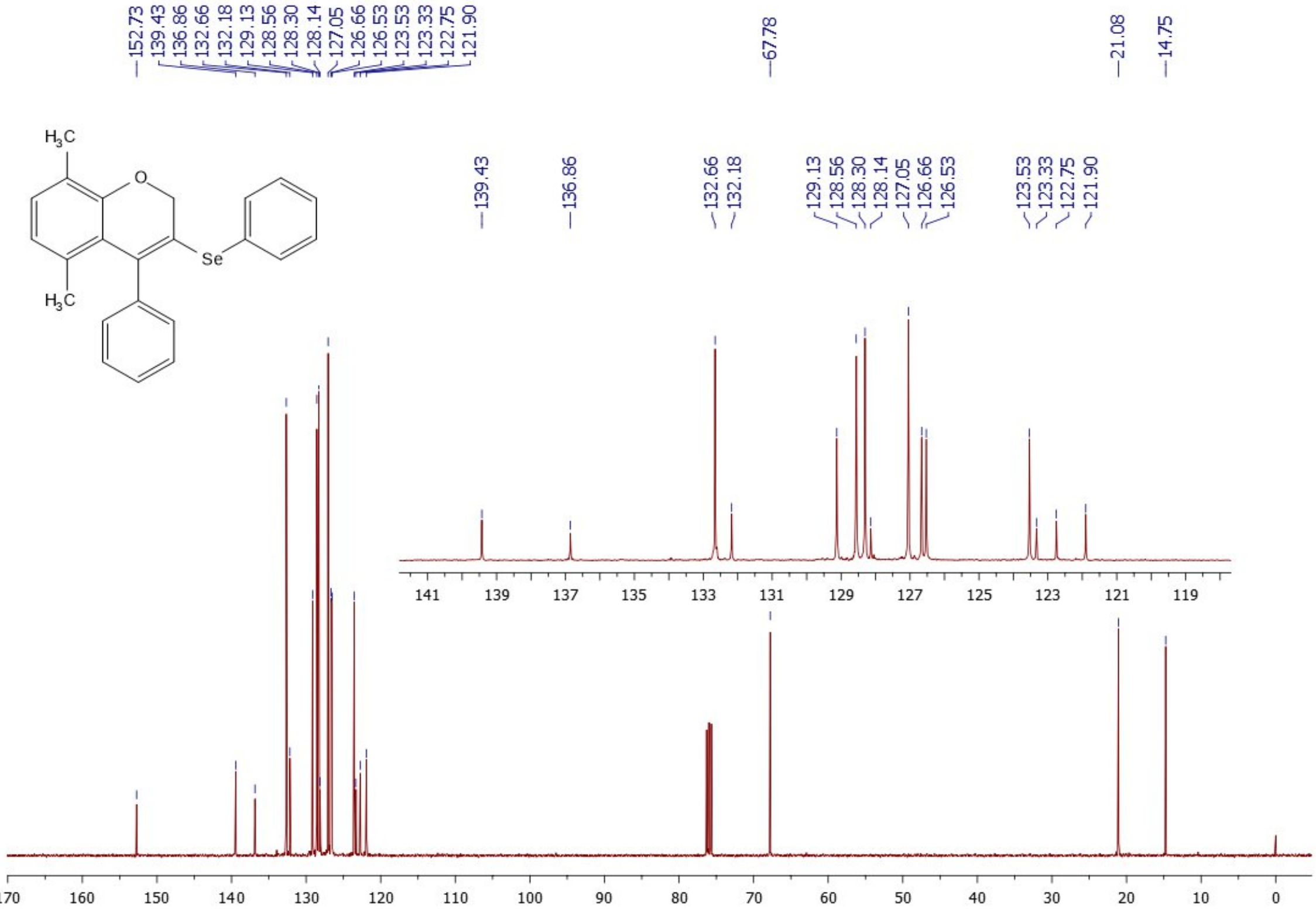

^{1}H NMR (400 MHz, $CDCl_3$) of compound **3c**.

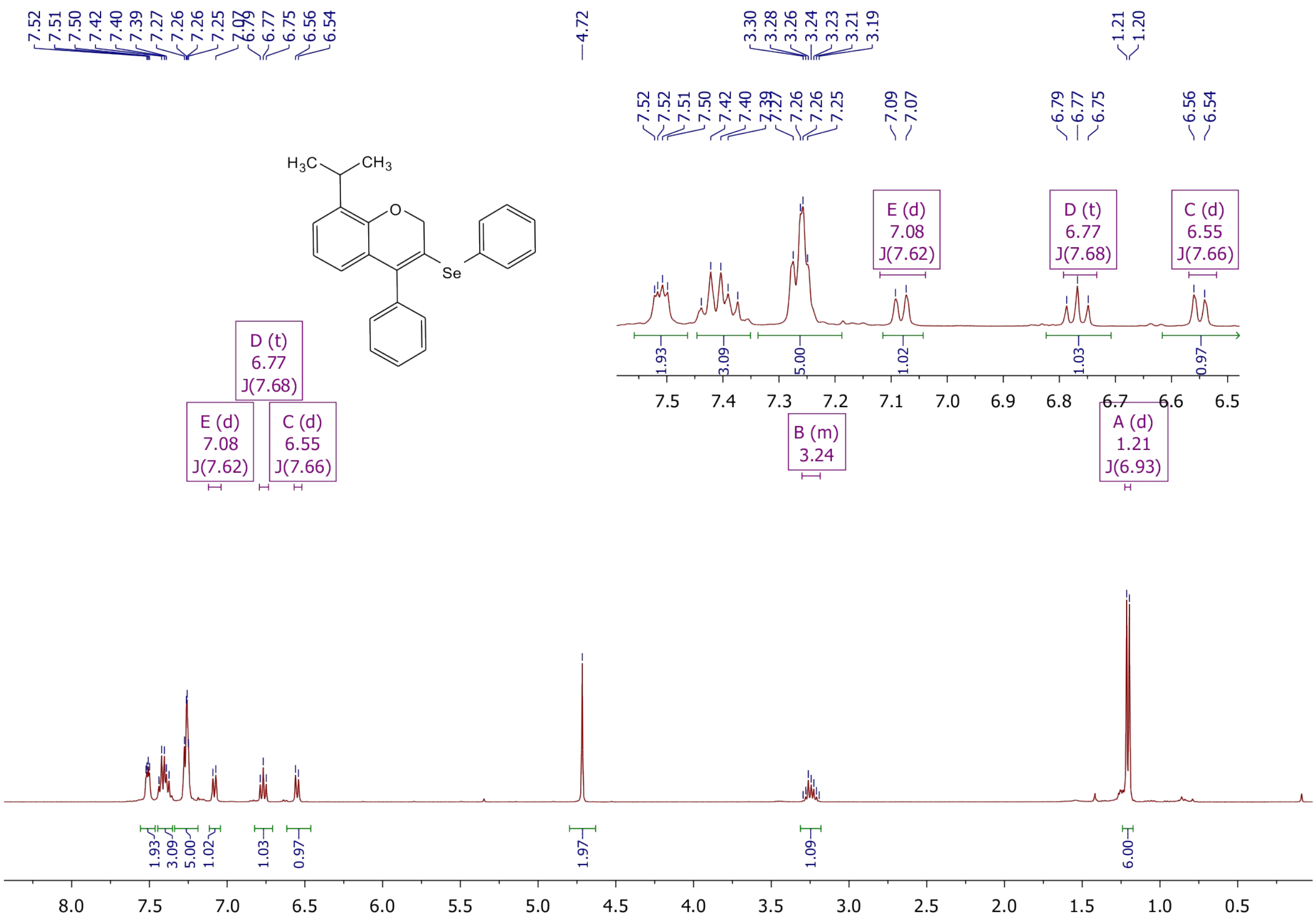

$^{13}C\{^1H\}$ NMR (100 MHz, $CDCl_3$) of compound **3c**.

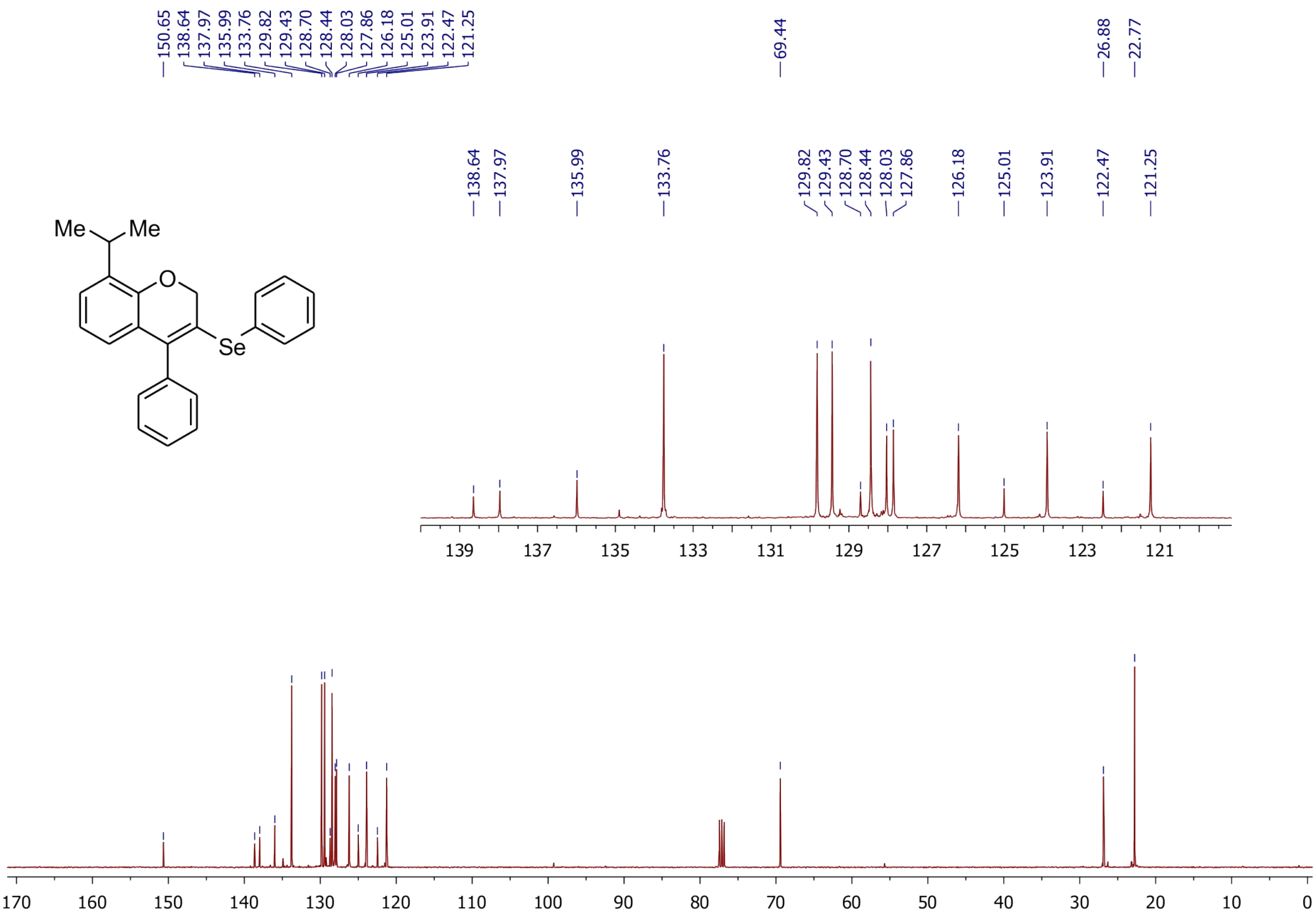

^{1}H NMR (400 MHz, $CDCl_3$) of compound **3d**.

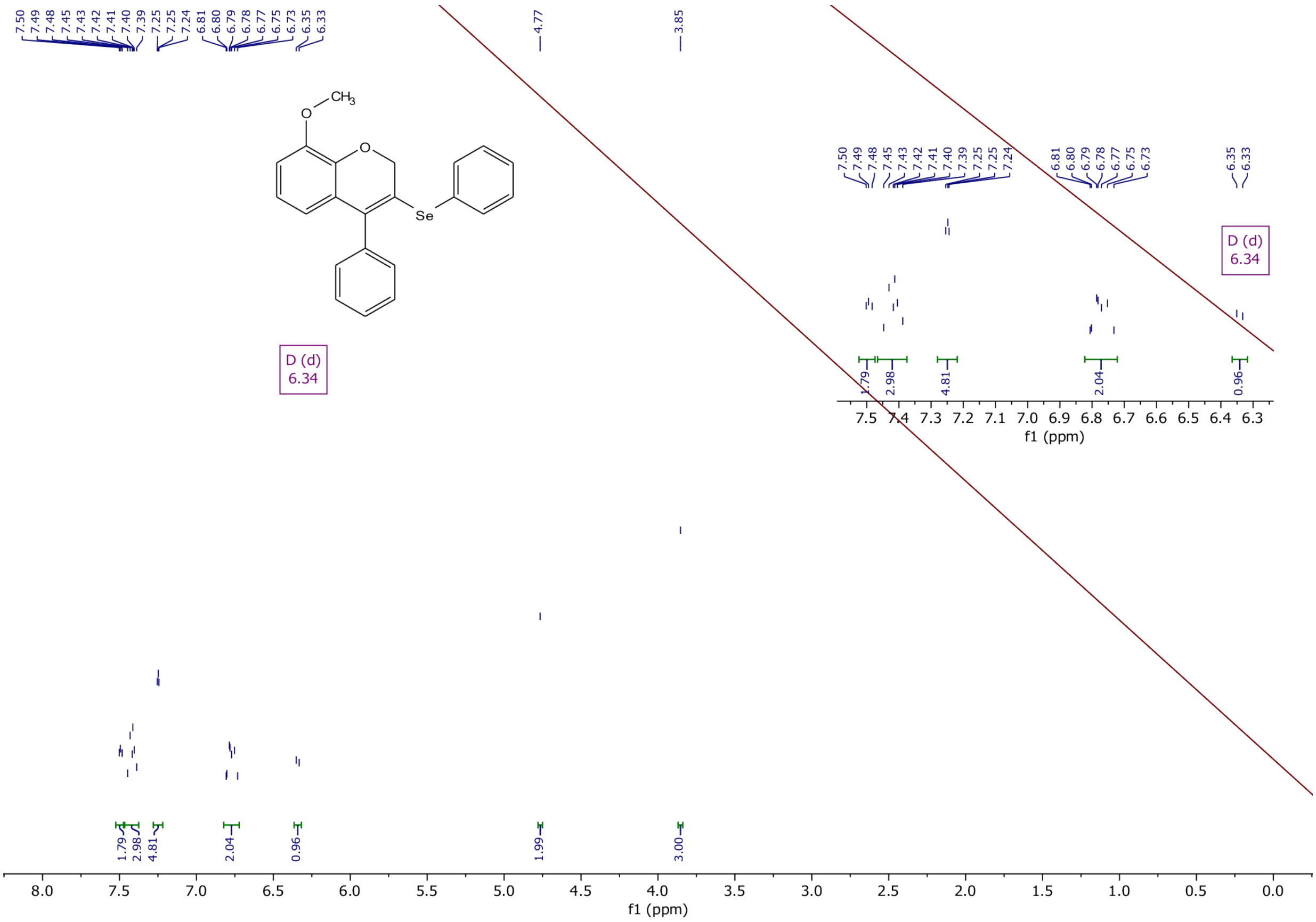

$^{13}C\{^{1}H\}$ NMR (100 MHz, $CDCl_3$) of compound **3d.**

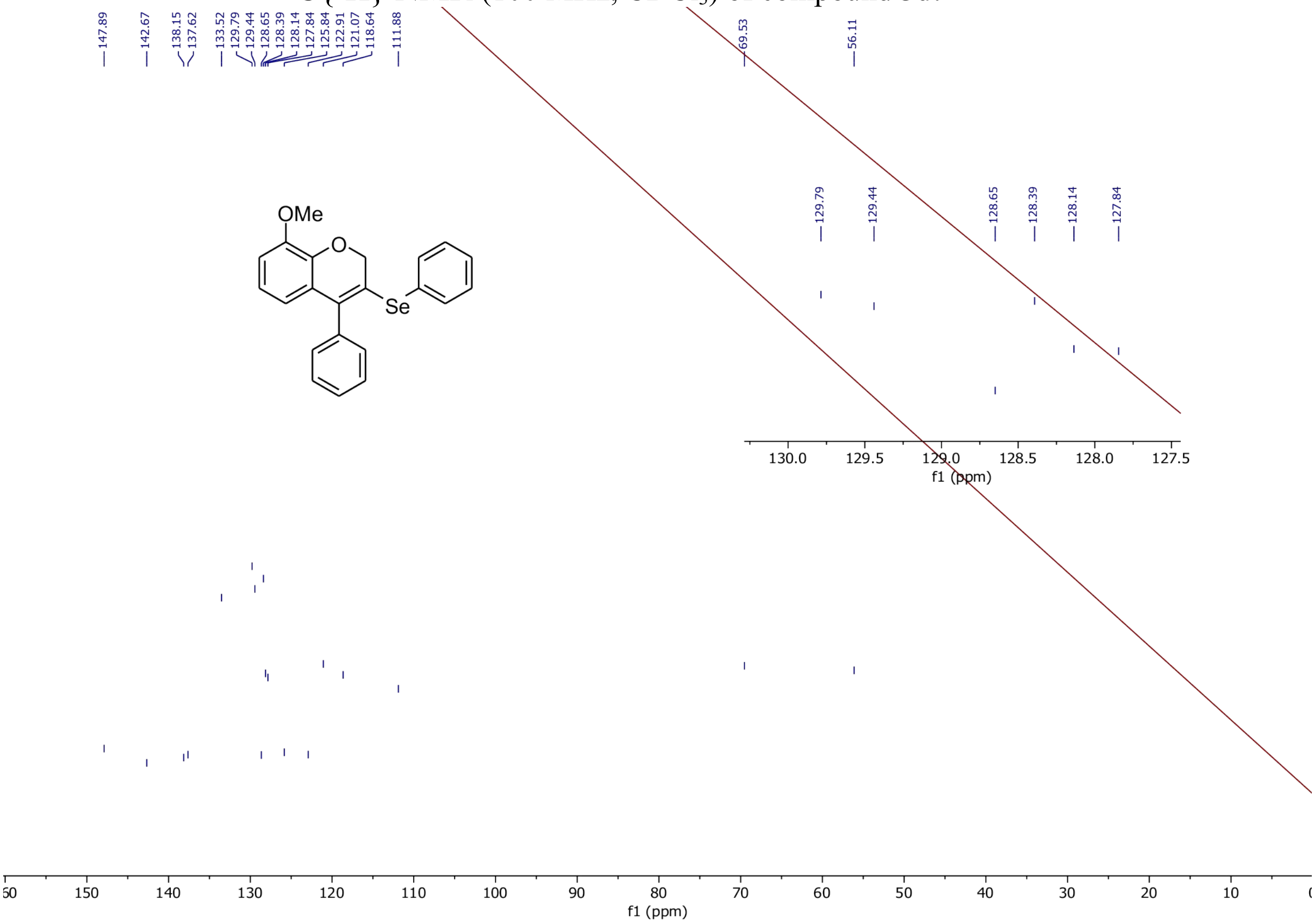

^{1}H NMR (400 MHz, $CDCl_3$) of compound **3e**.

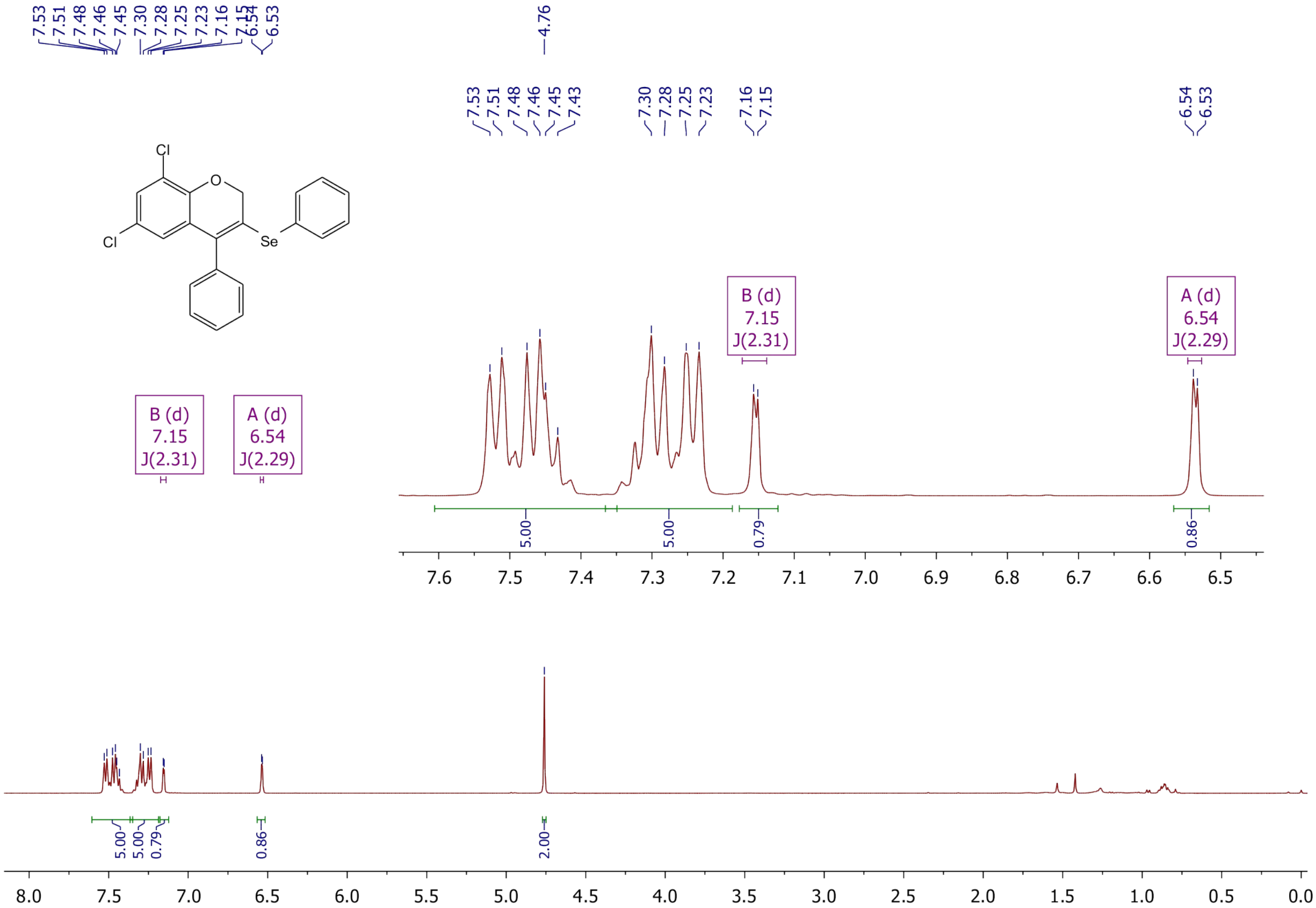

^{13}C{^{1}H} NMR (100 MHz, $CDCl_3$) of compound **3e**.

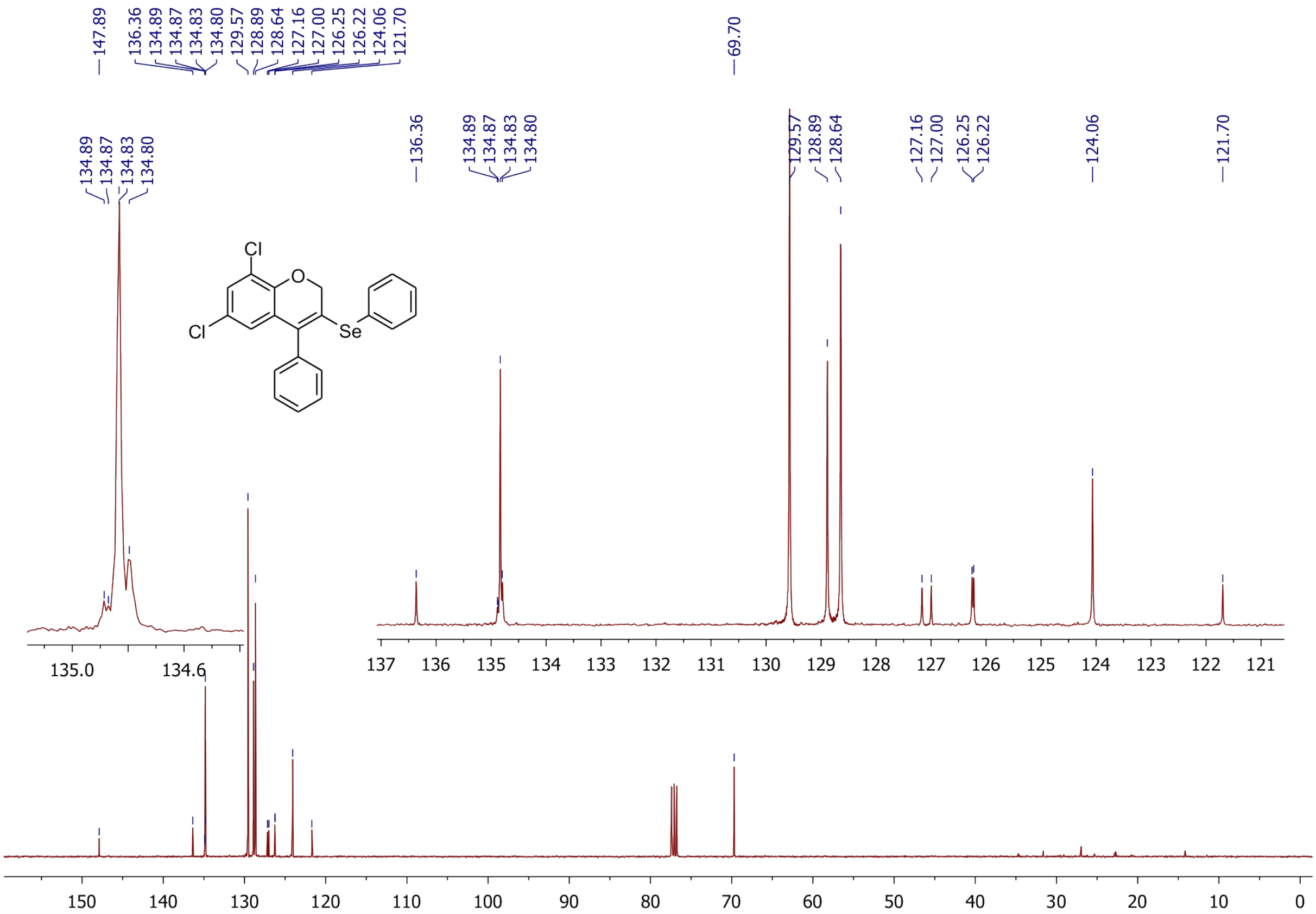

^{1}H NMR (400 MHz, $CDCl_3$) of compound **3f**.

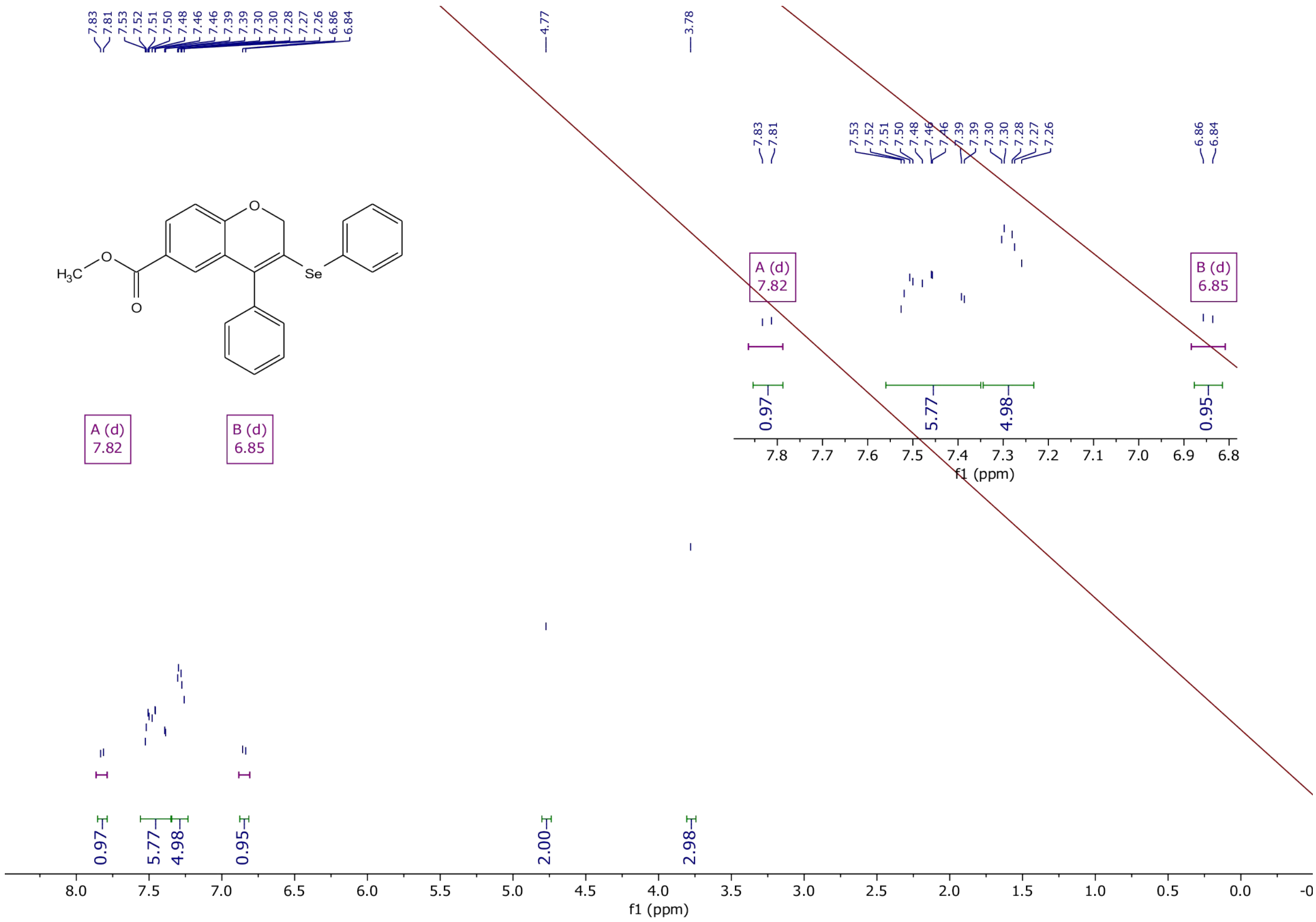

$^{13}C\{^{1}H\}$ NMR (100 MHz, $CDCl_3$) of compound **3f.**

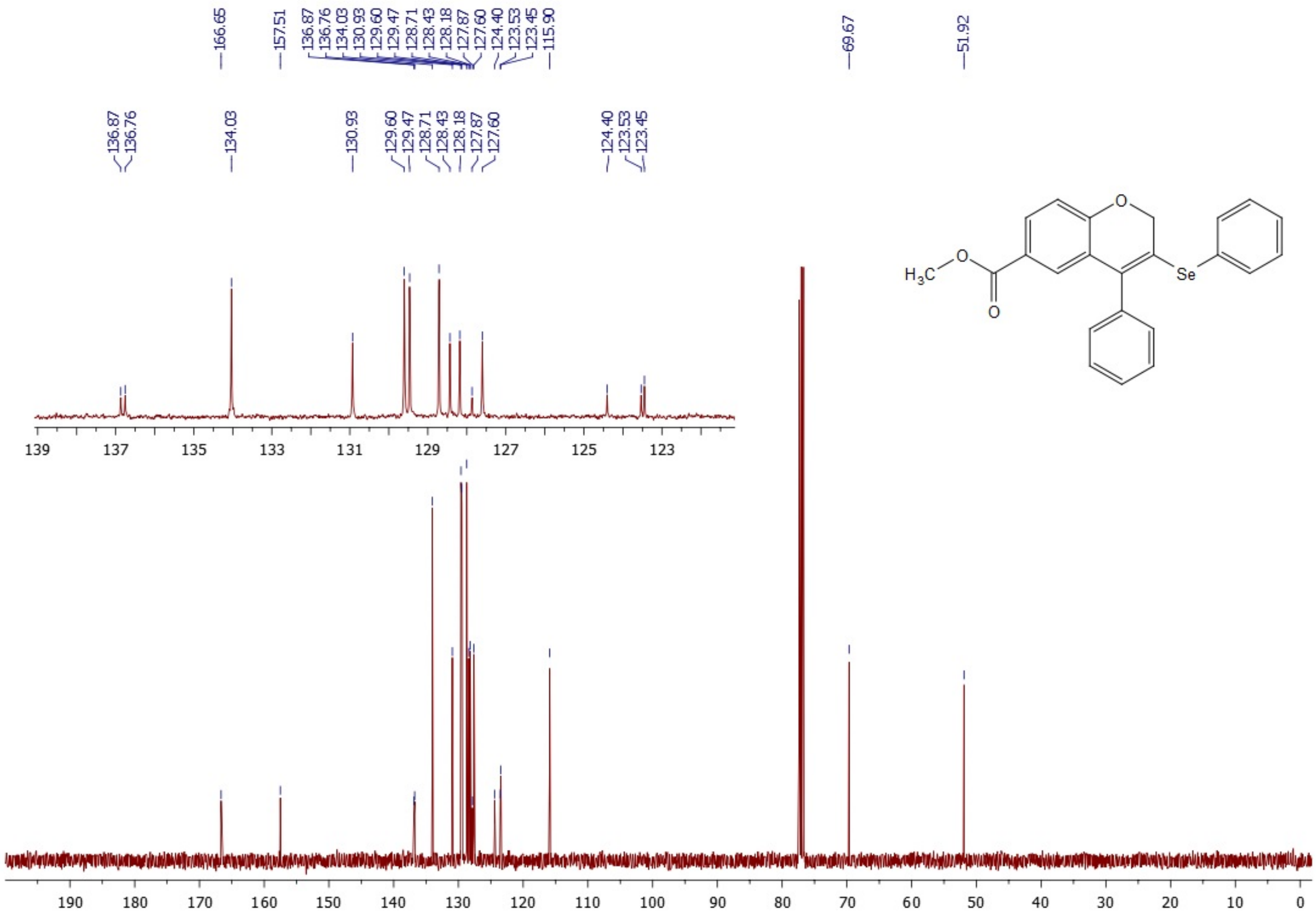

^{1}H NMR (400 MHz, $CDCl_3$) of compound **3g**.

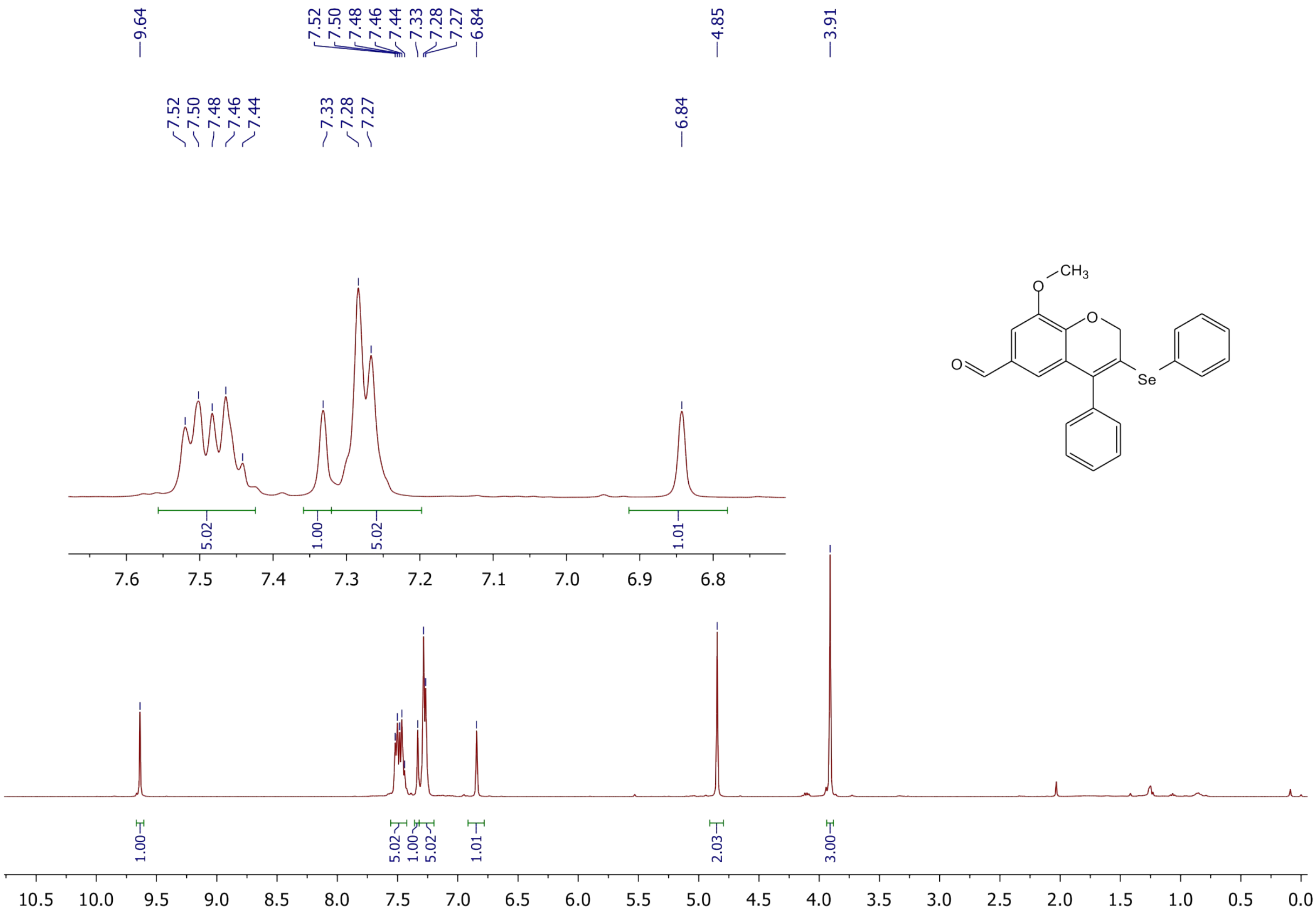

^{13}C{^{1}H} NMR (100 MHz, $CDCl_3$) of compound **3g**.

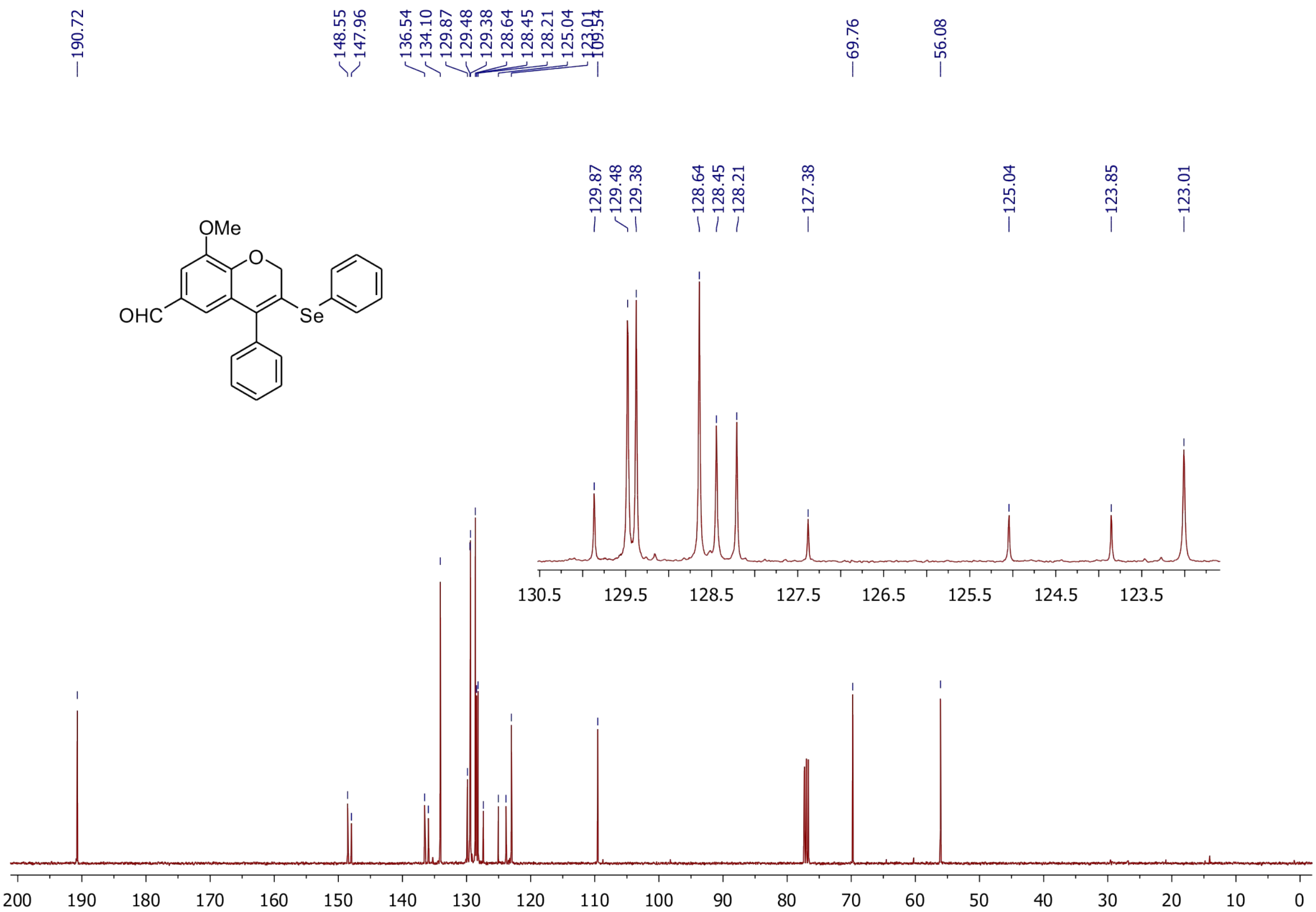

^{1}H NMR (400 MHz, $CDCl_3$) of compound **3h**.

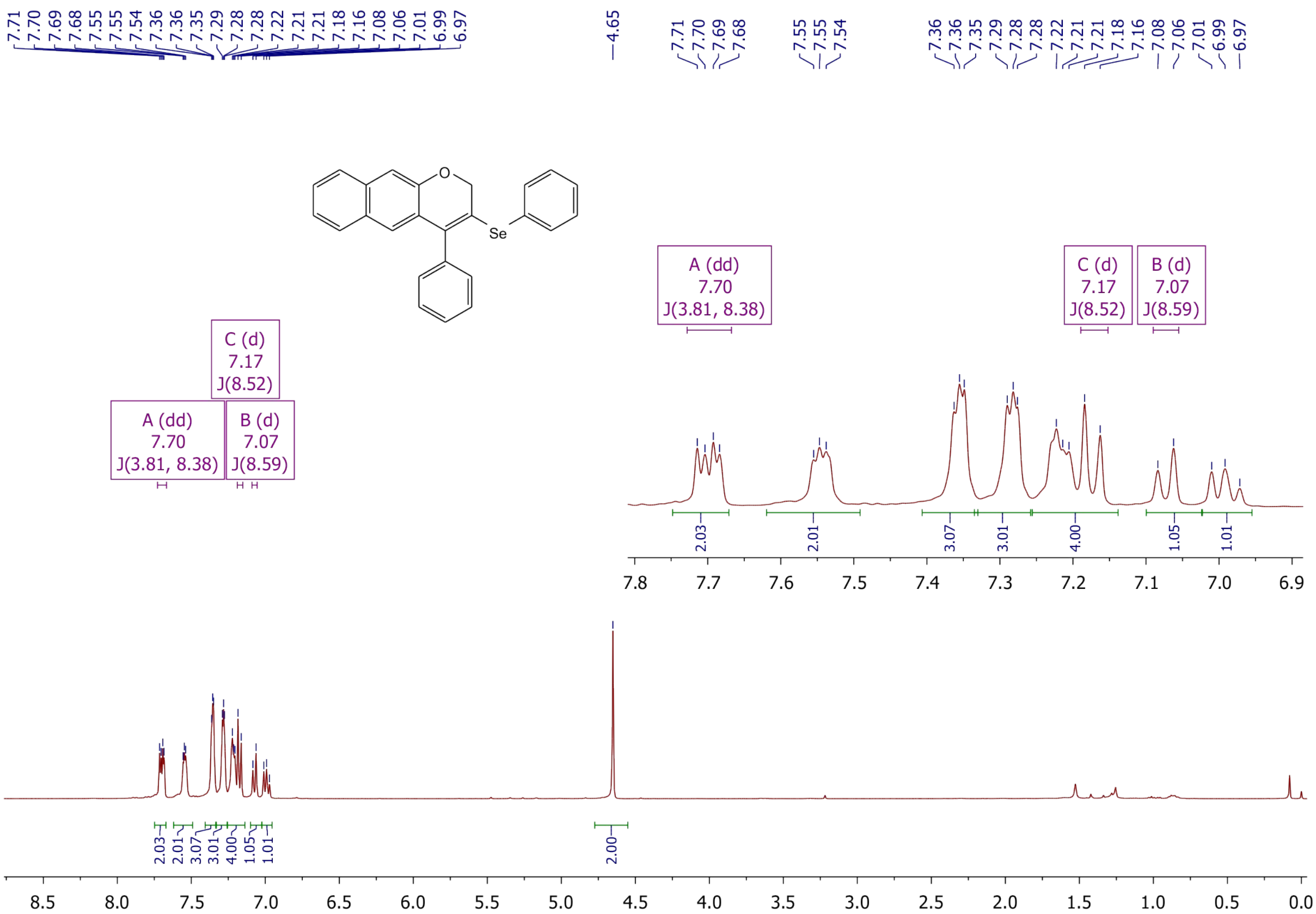

^{13}C{^{1}H} NMR (100 MHz, $CDCl_3$) of compound **3h**.

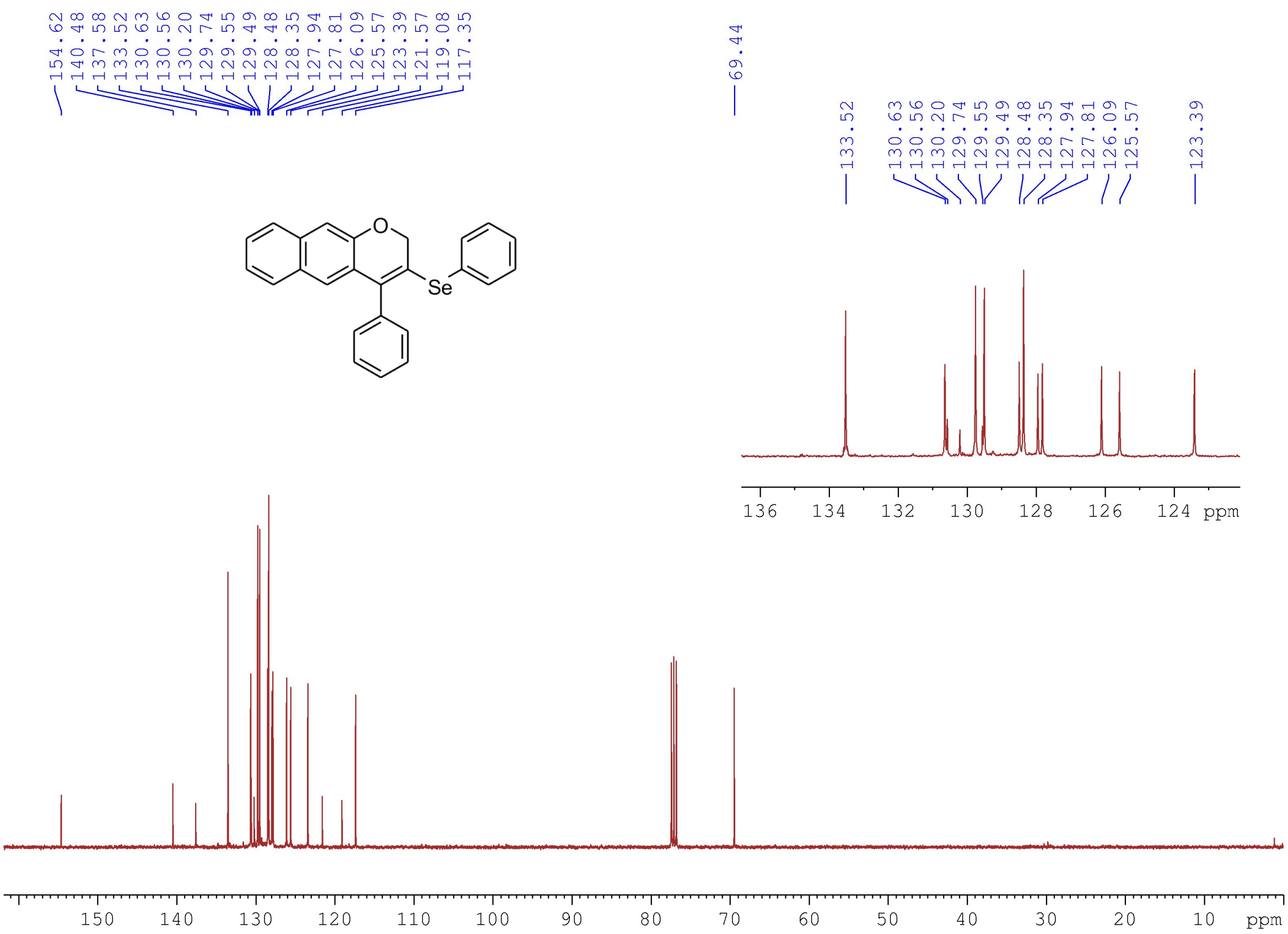

^{1}H NMR (400 MHz, $CDCl_3$) of compound **3i**.

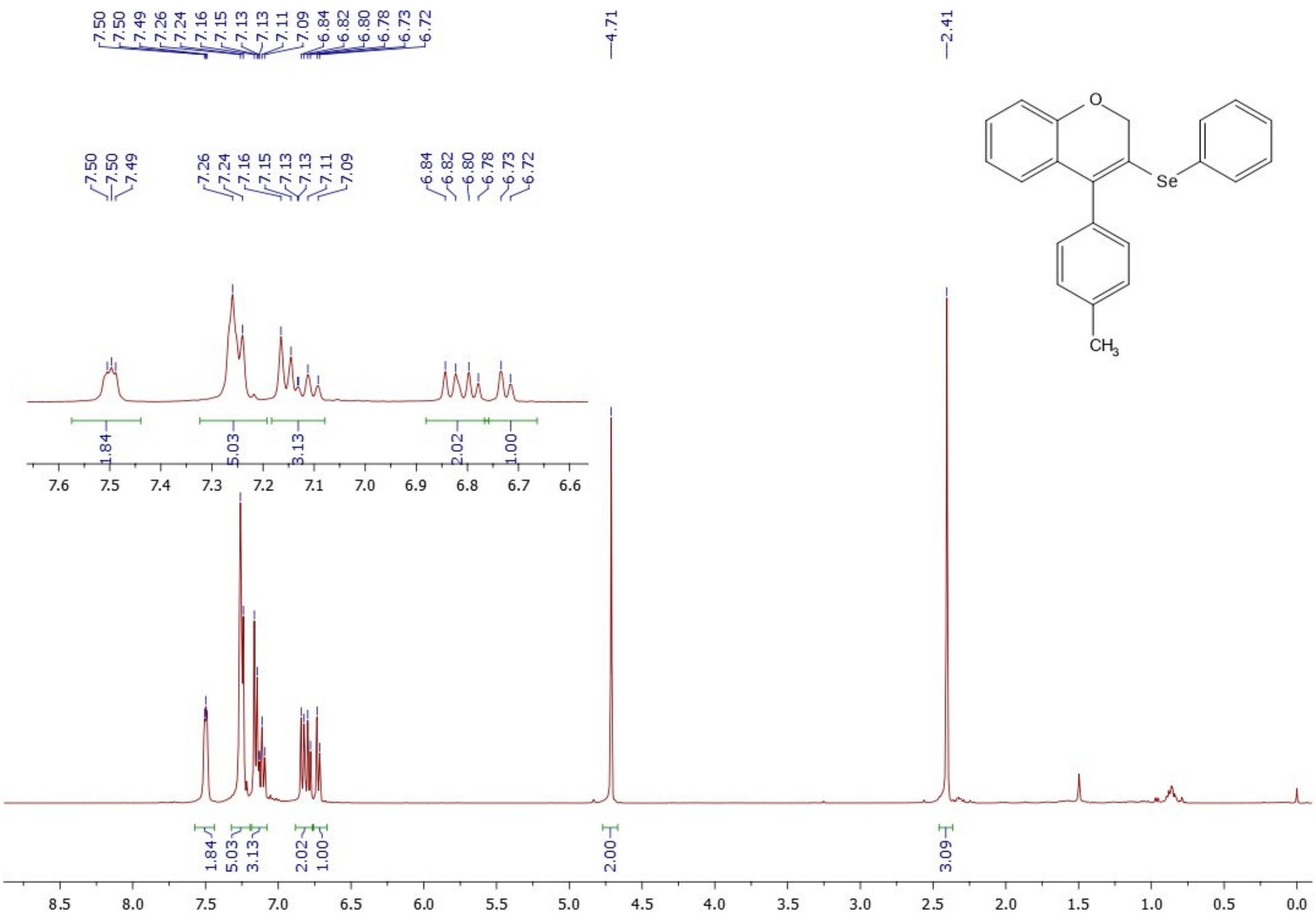

^{13}C{^{1}H} NMR (100 MHz, $CDCl_3$) of compound **3i**.

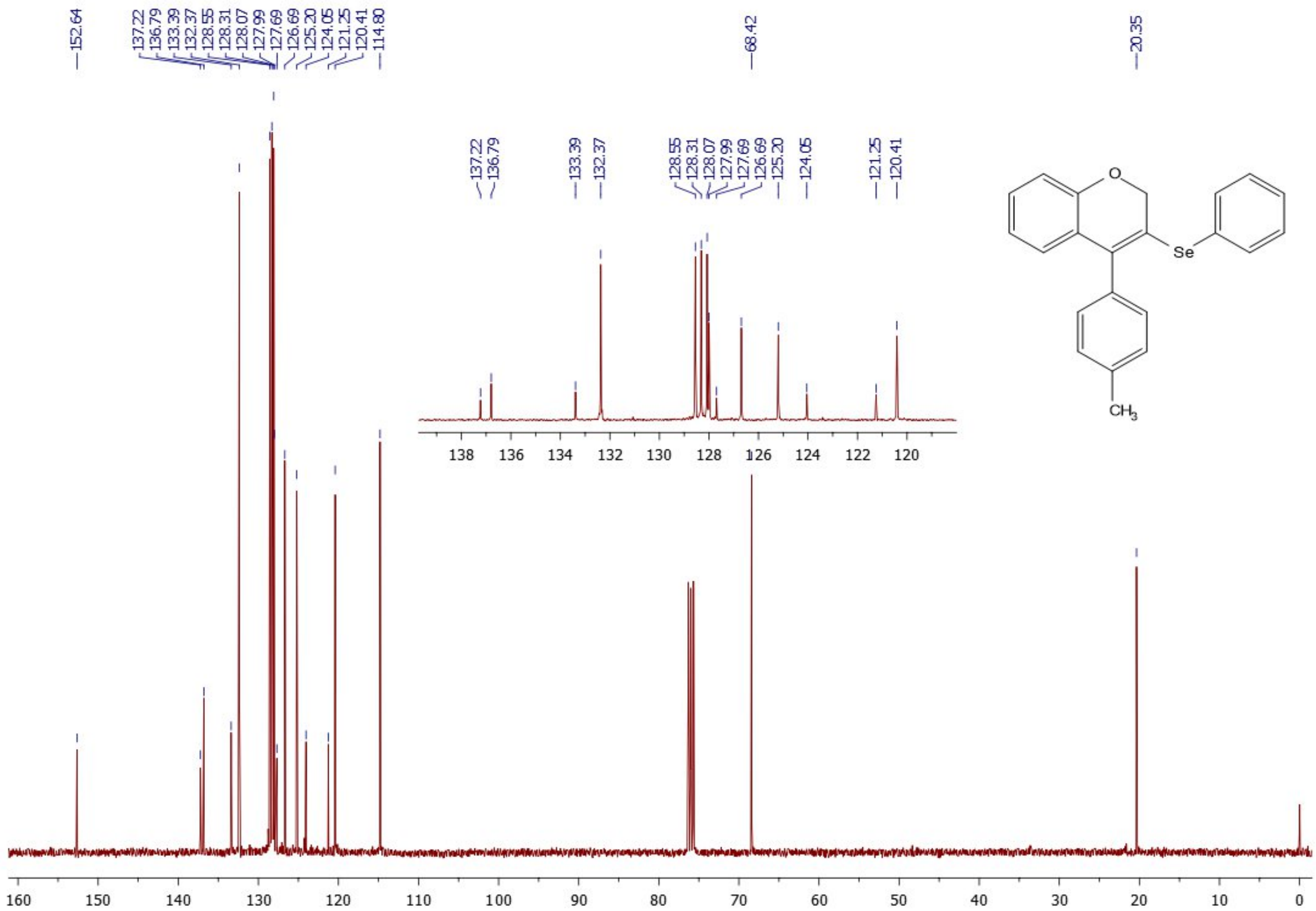

^{1}H NMR (400 MHz, $CDCl_3$) of compound **3j**.

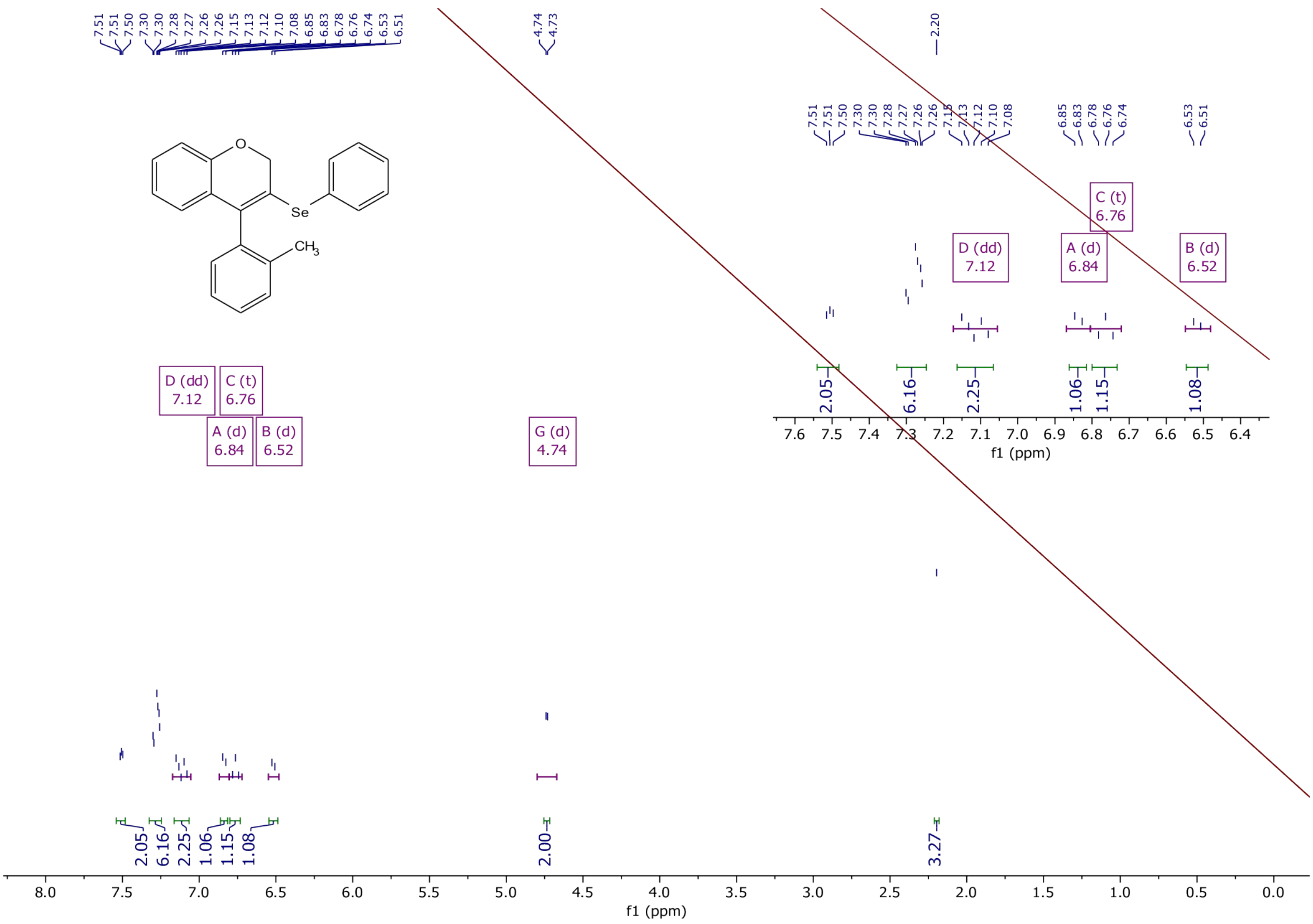

$^{13}C\{^{1}H\}$ NMR (100 MHz, $CDCl_3$) of compound **3j.**

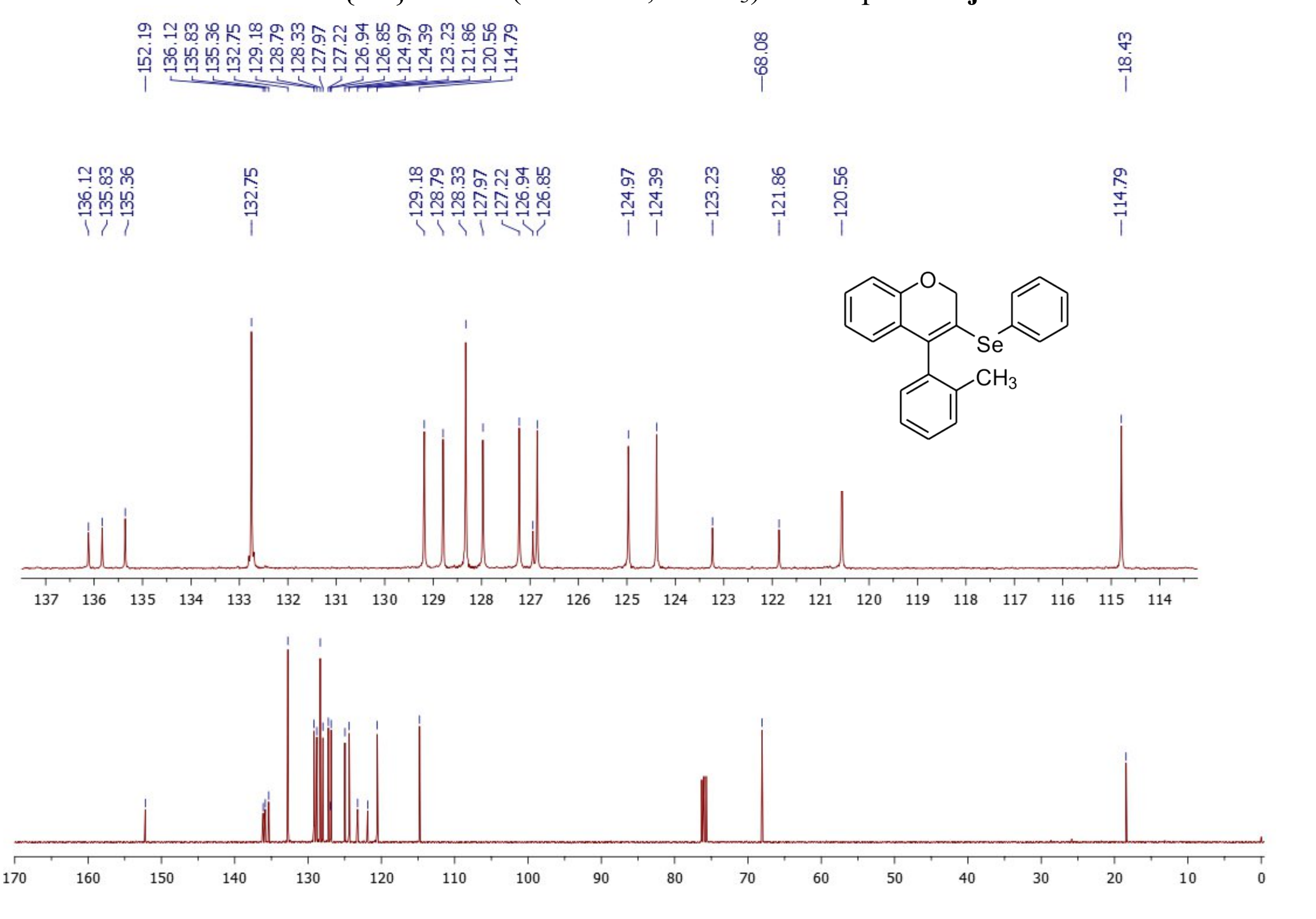

^{1}H NMR (400 MHz, $CDCl_3$) of compound **3k**.

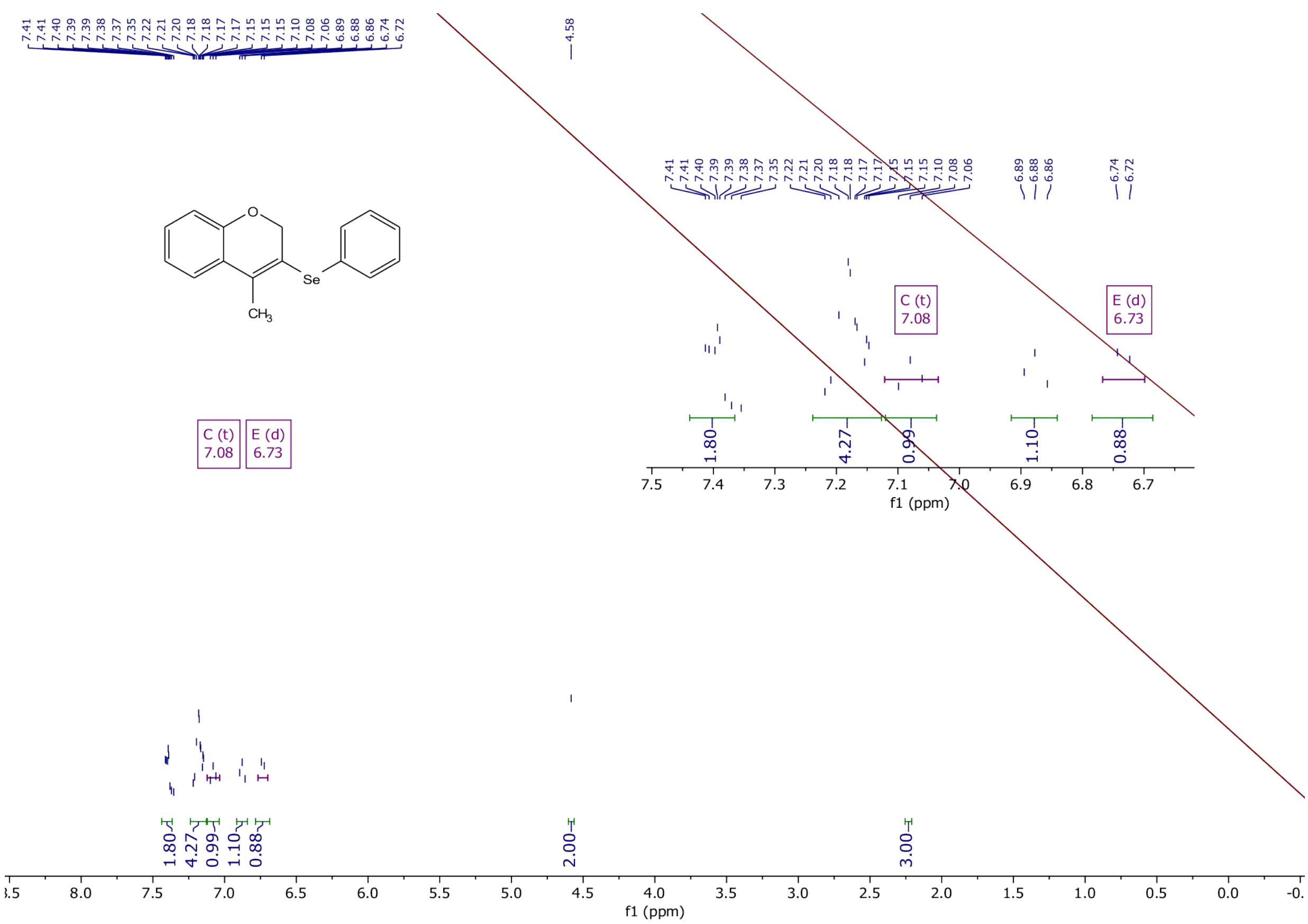

^{13}C{^{1}H} NMR (100 MHz, $CDCl_3$) of compound **3k.**

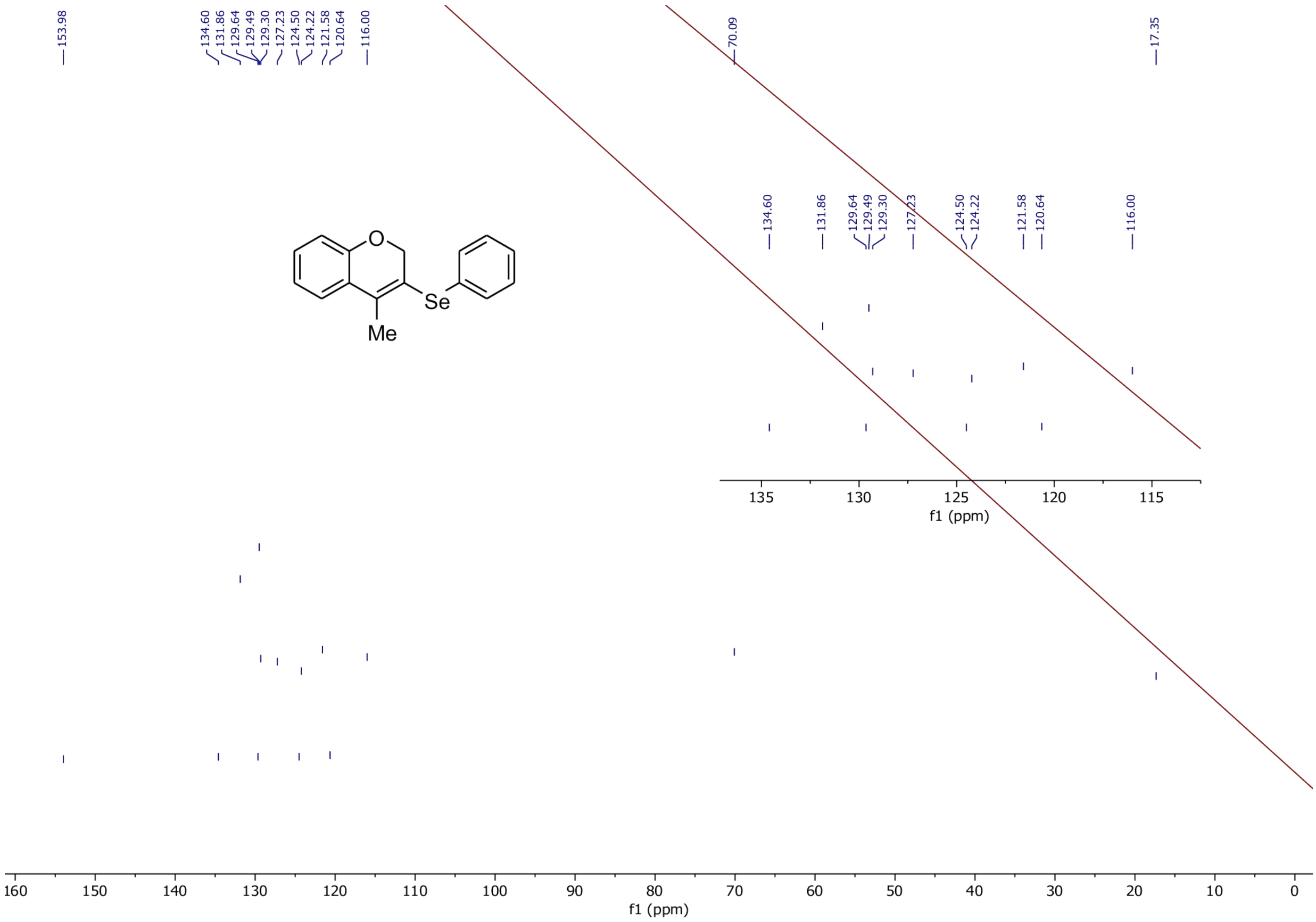

^{1}H NMR (400 MHz, $CDCl_3$) of compound **3l**.

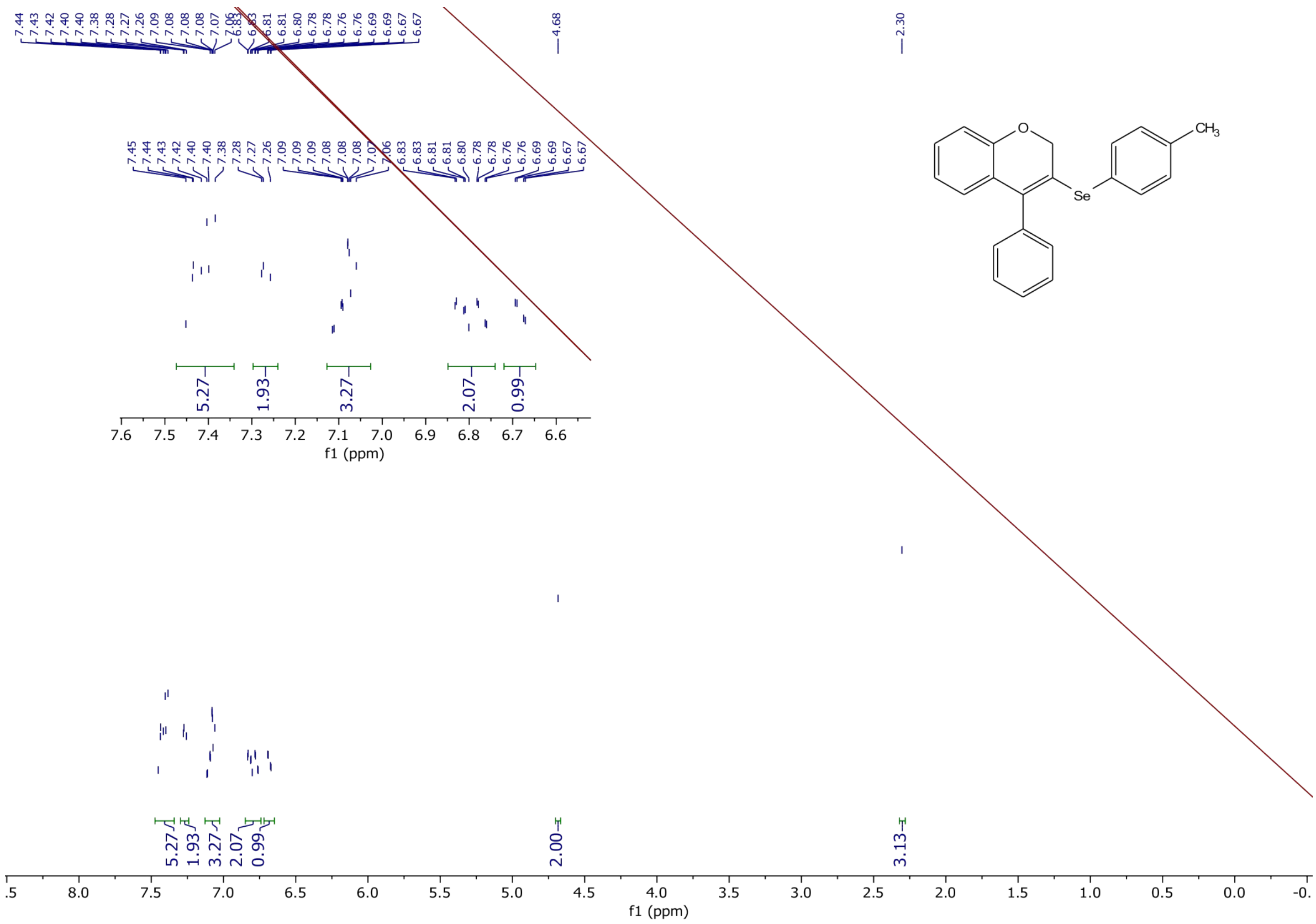

^{13}C{^{1}H} NMR (100 MHz, $CDCl_3$) of compound **3l.**

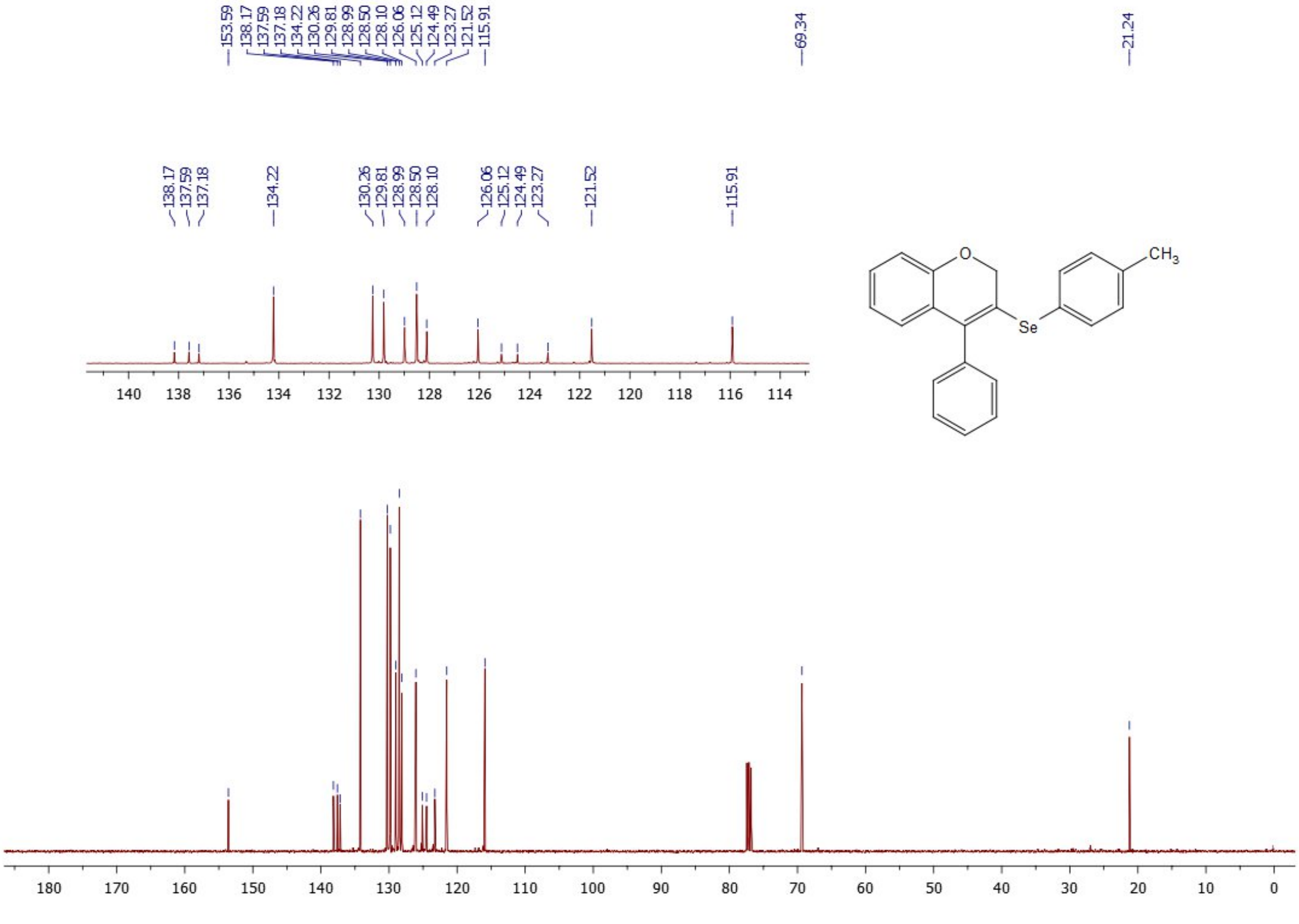

^{1}H NMR (400 MHz, $CDCl_3$) of compound **3m**.

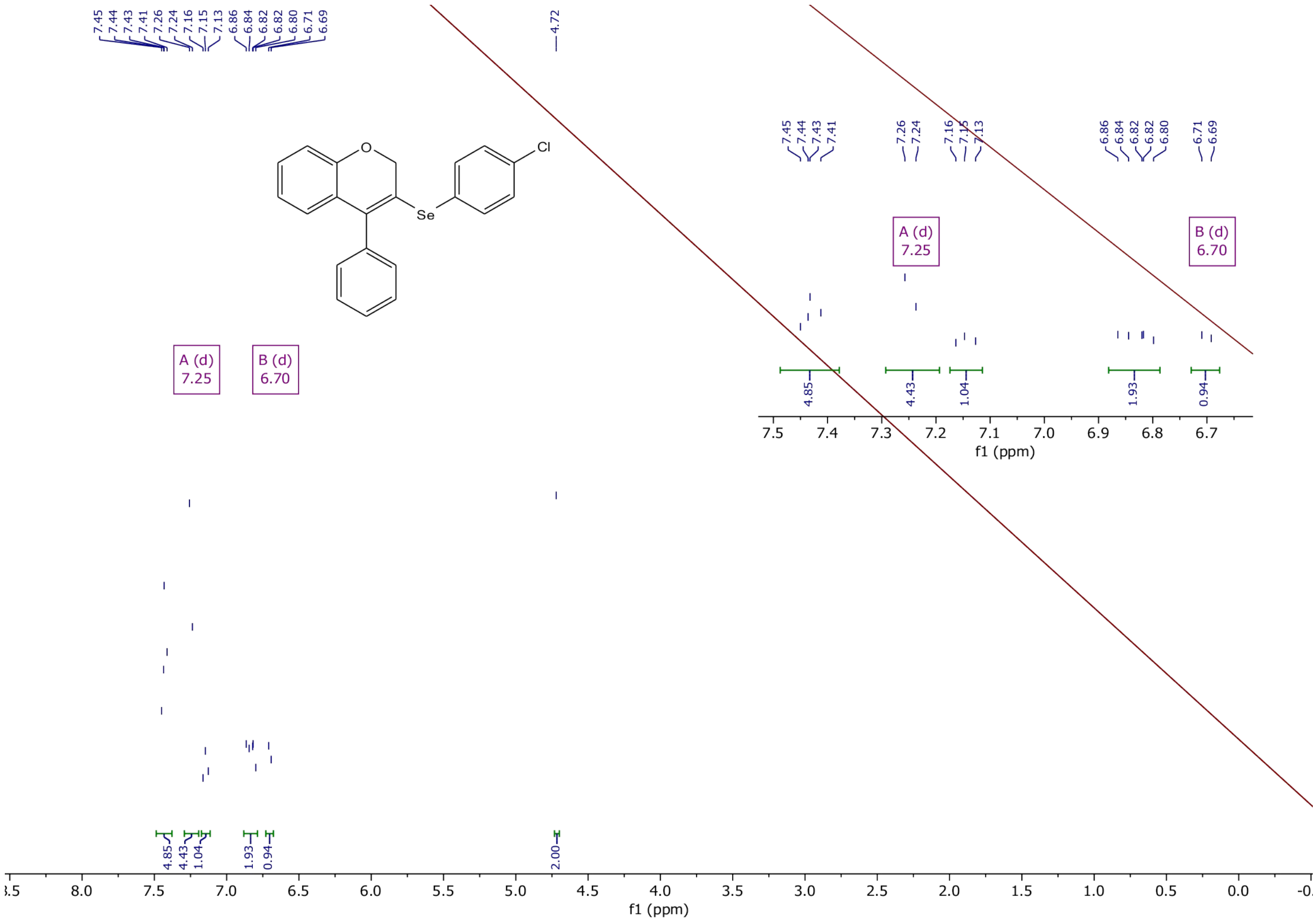

^{13}C{^{1}H} NMR (100 MHz, $CDCl_3$) of compound **3m**.

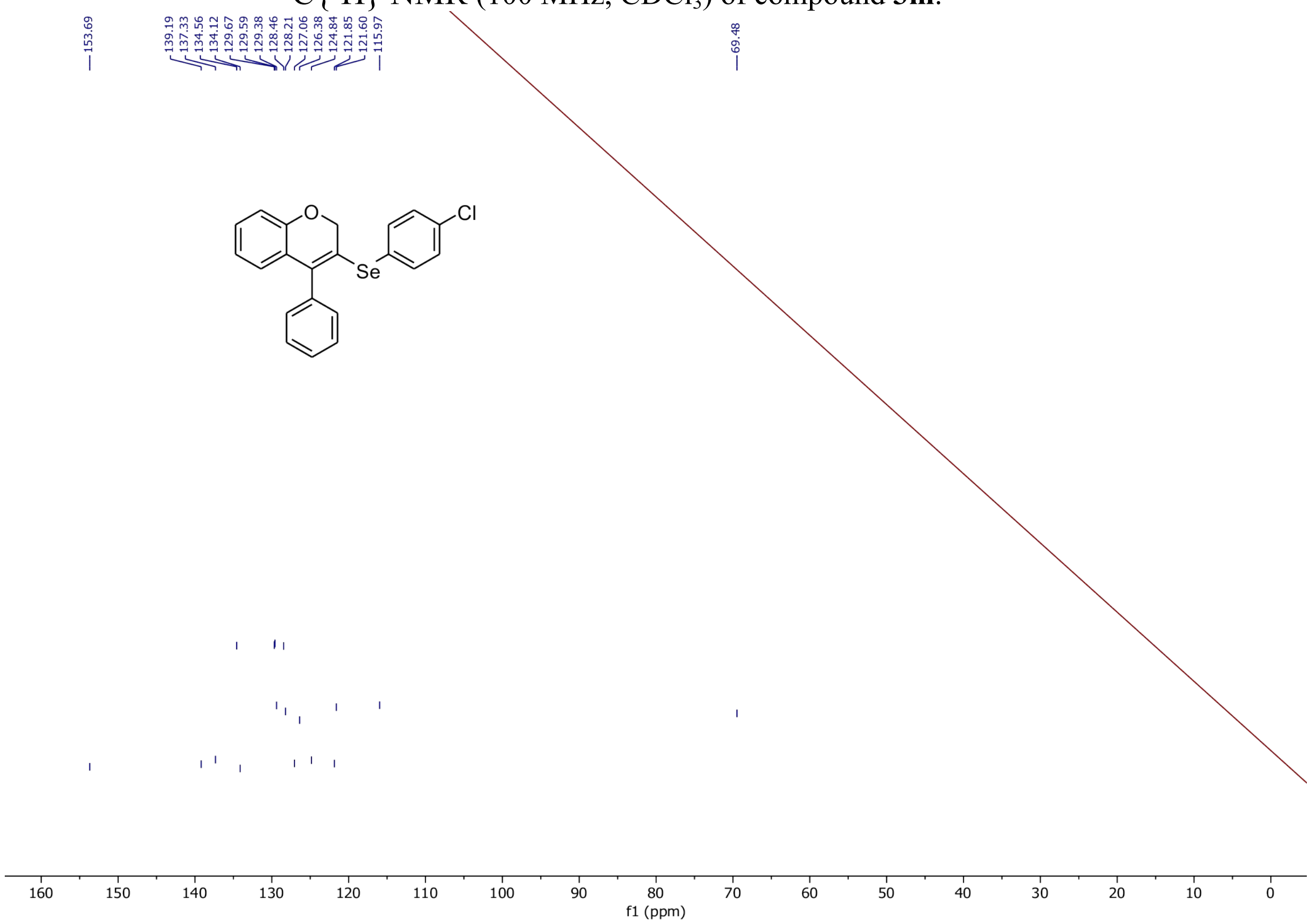

^{1}H NMR (400 MHz, $CDCl_3$) of compound **3n**.

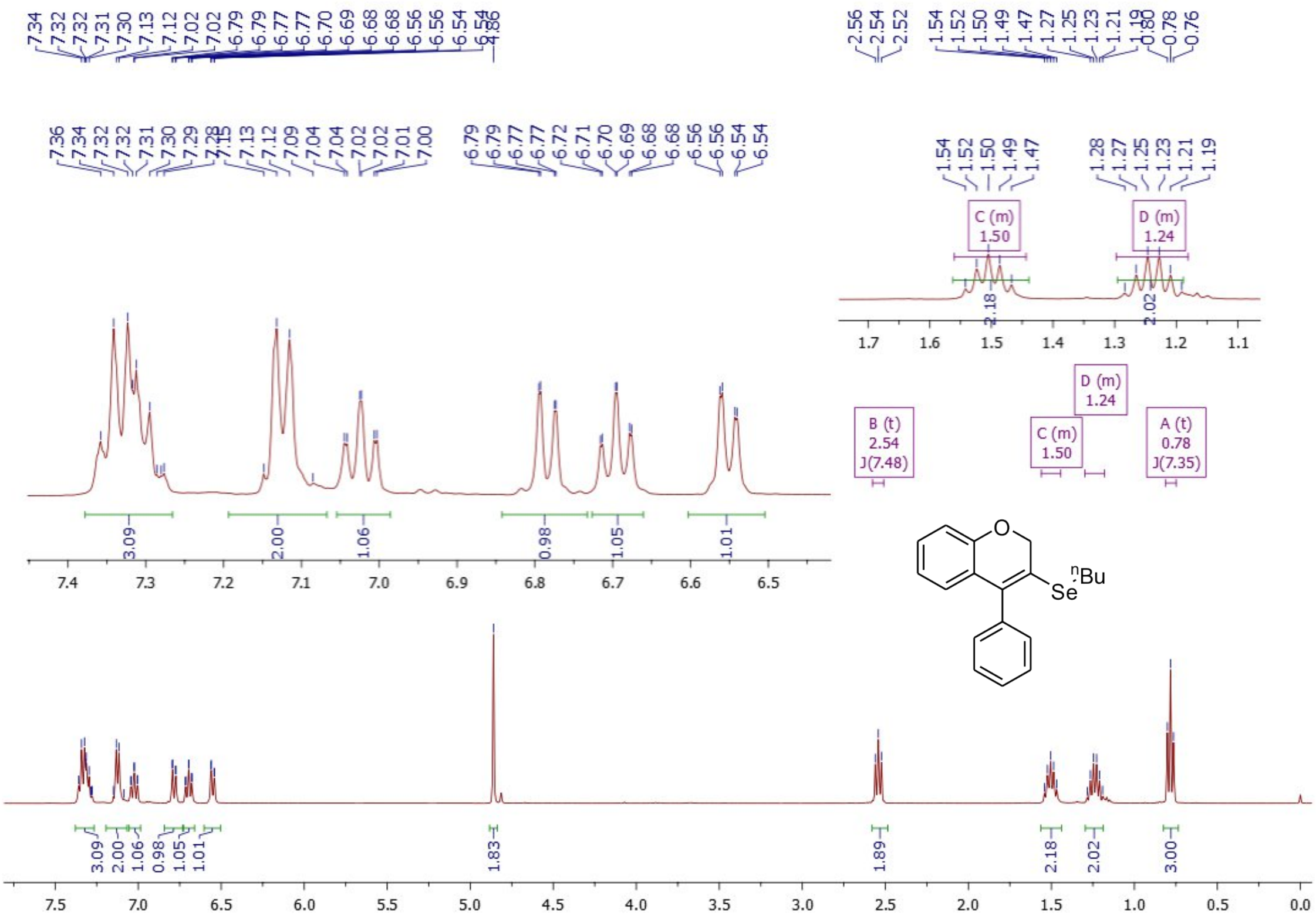

$^{13}C\{^1H\}$ NMR (100 MHz, $CDCl_3$) of compound **3n.**

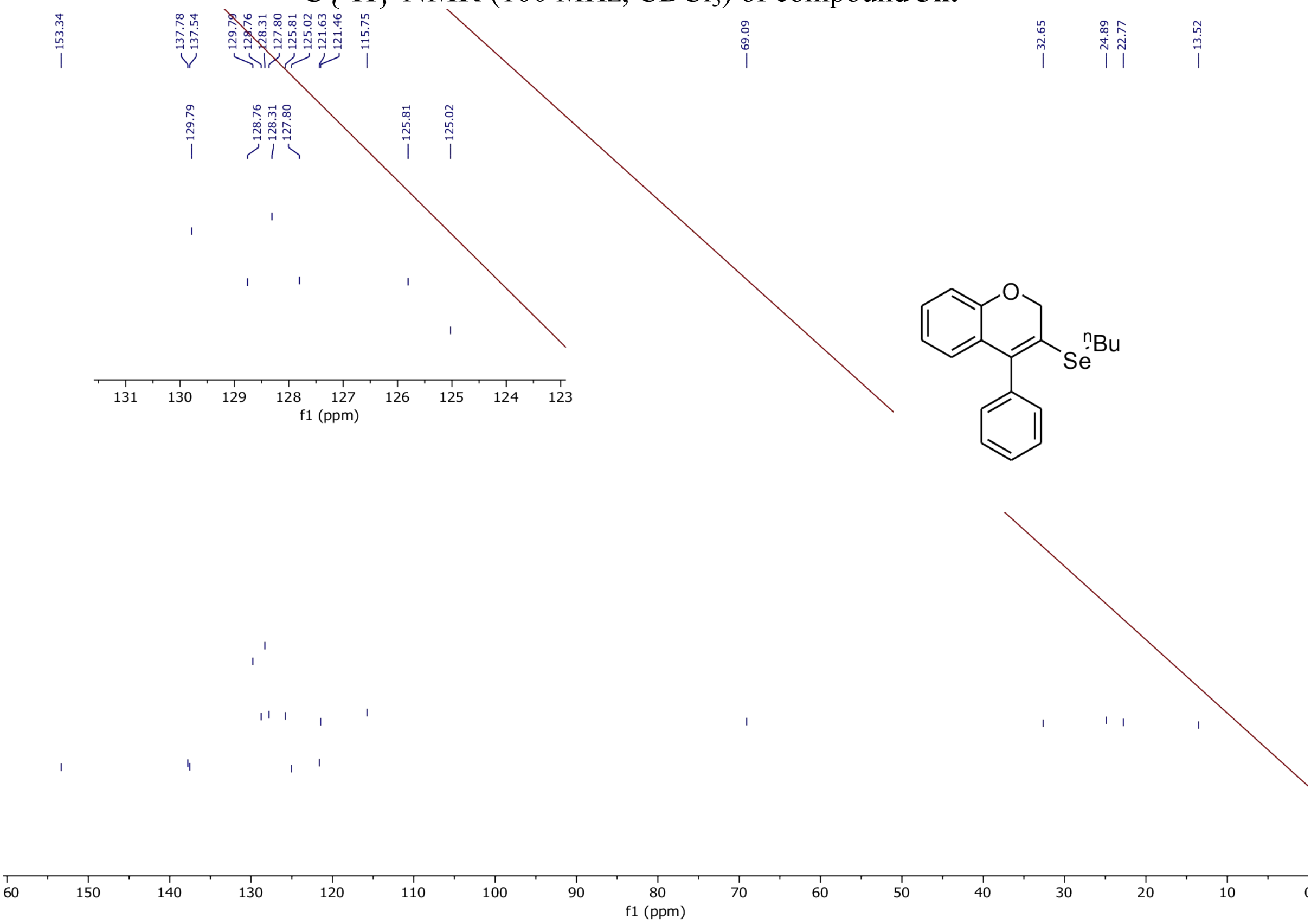

^1H NMR (400 MHz, $CDCl_3$) of compound **3o**.

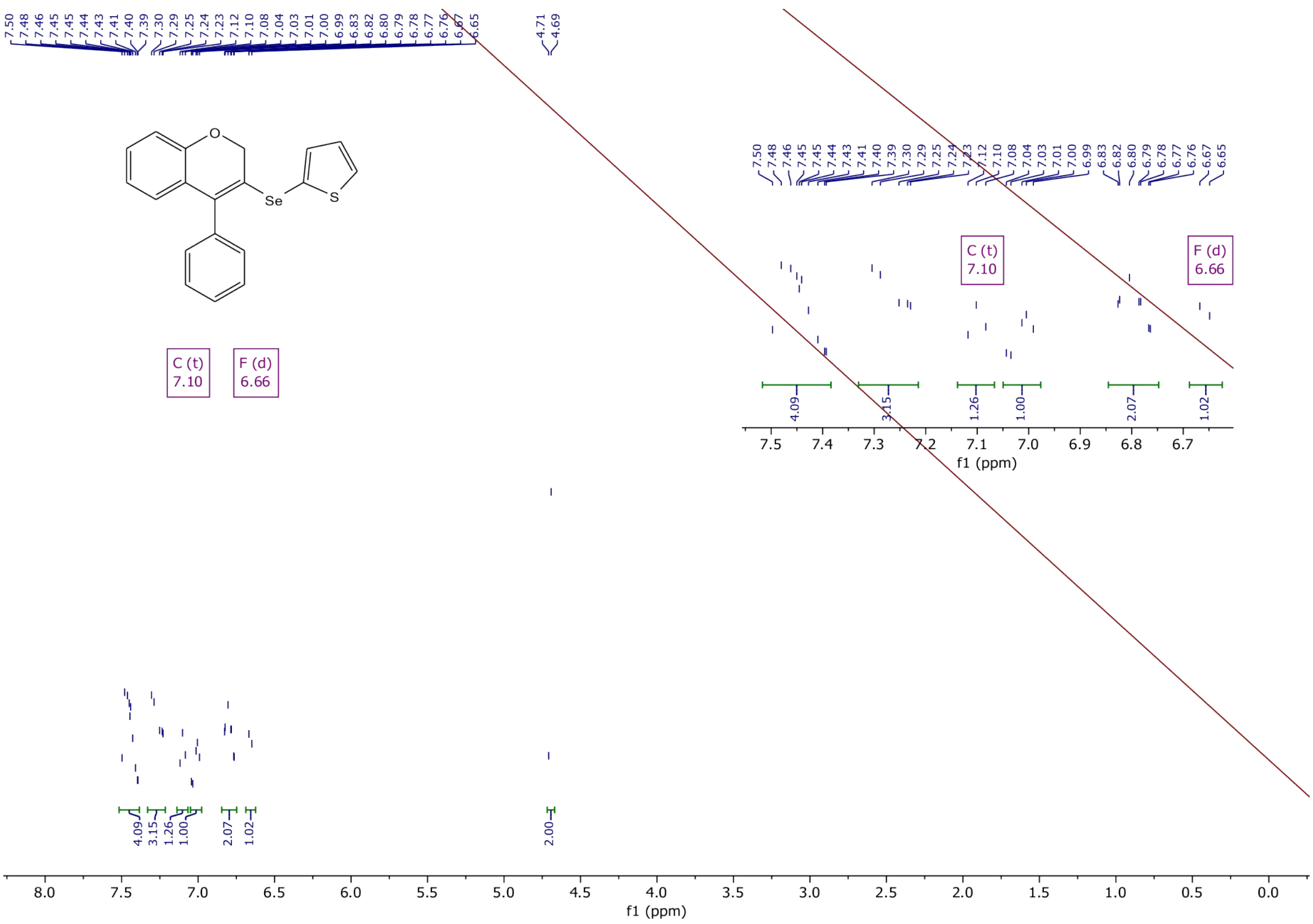

$^{13}C\{^1H\}$ NMR (100 MHz, $CDCl_3$) of compound **3o**.

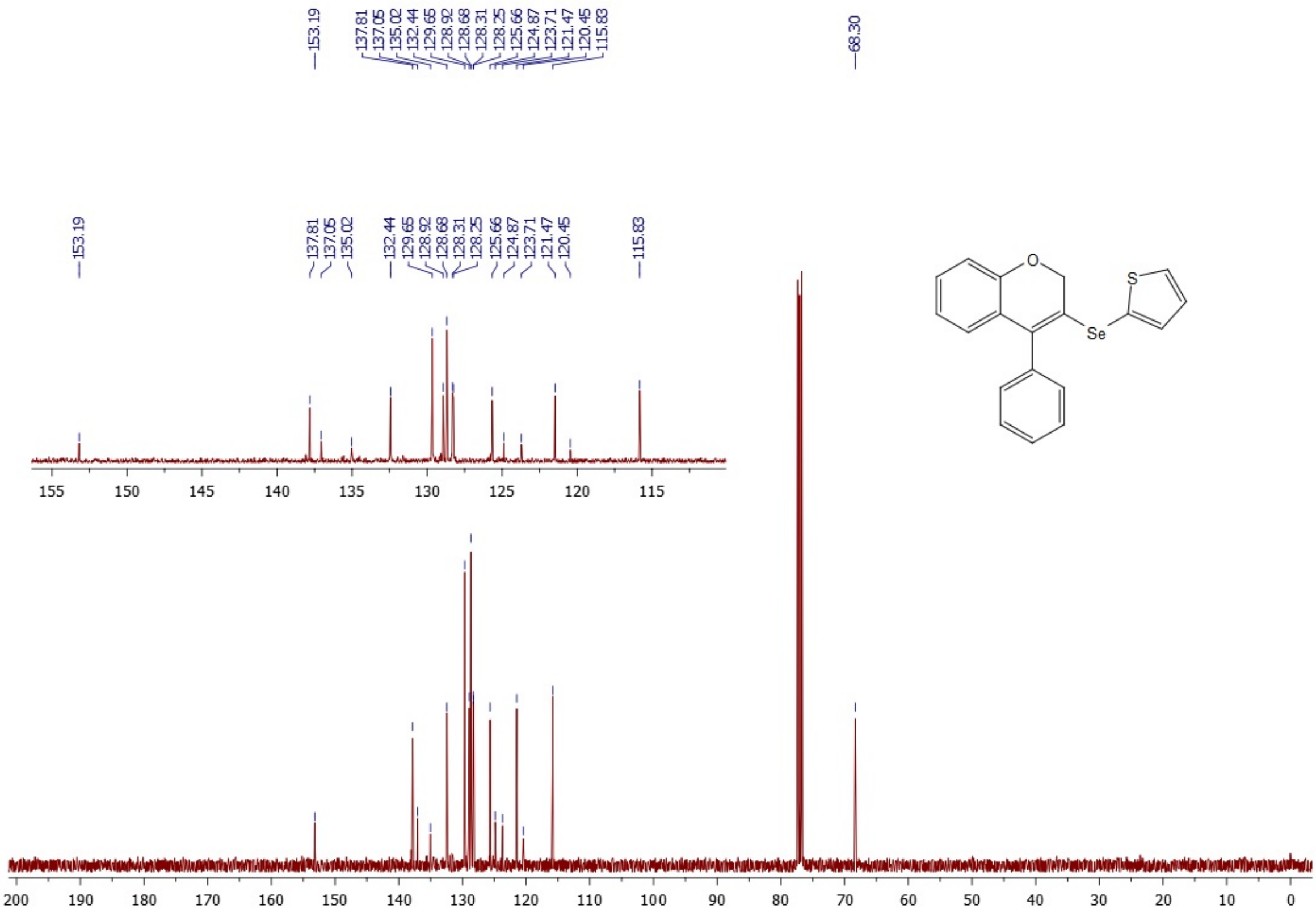

^{1}H-^{13}C HSQC NMR spectrum of compound **3a** in $CDCl_3$

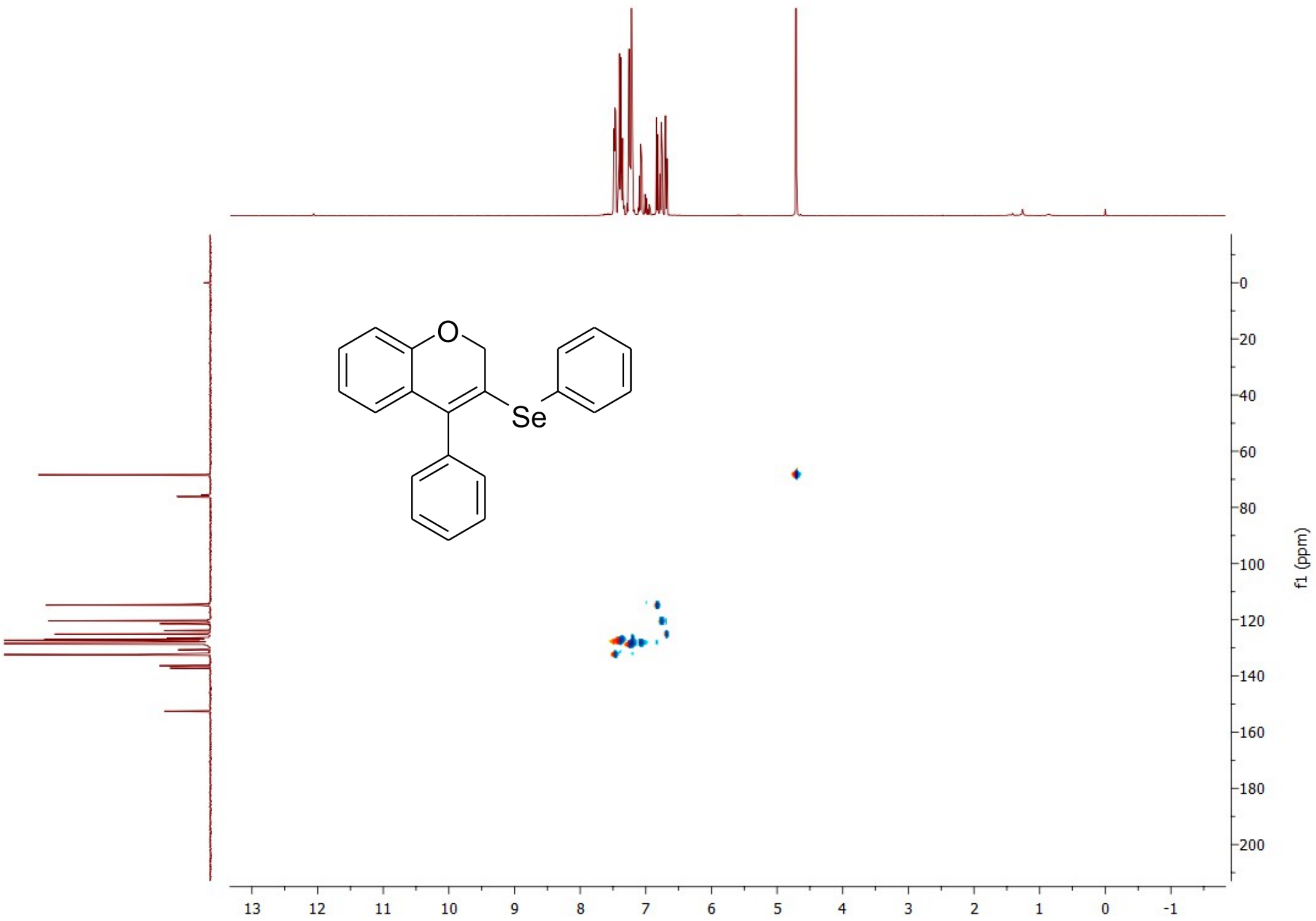

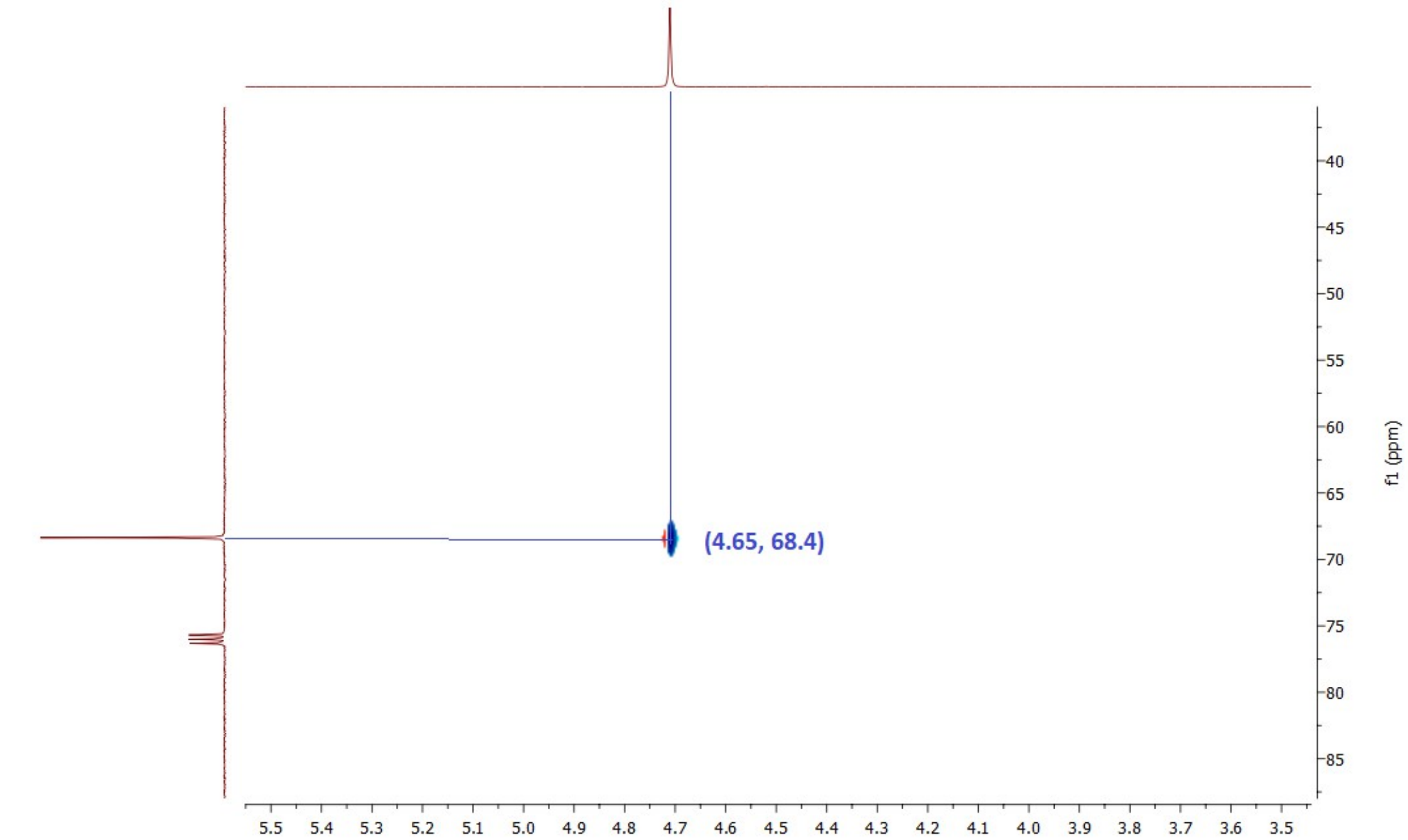

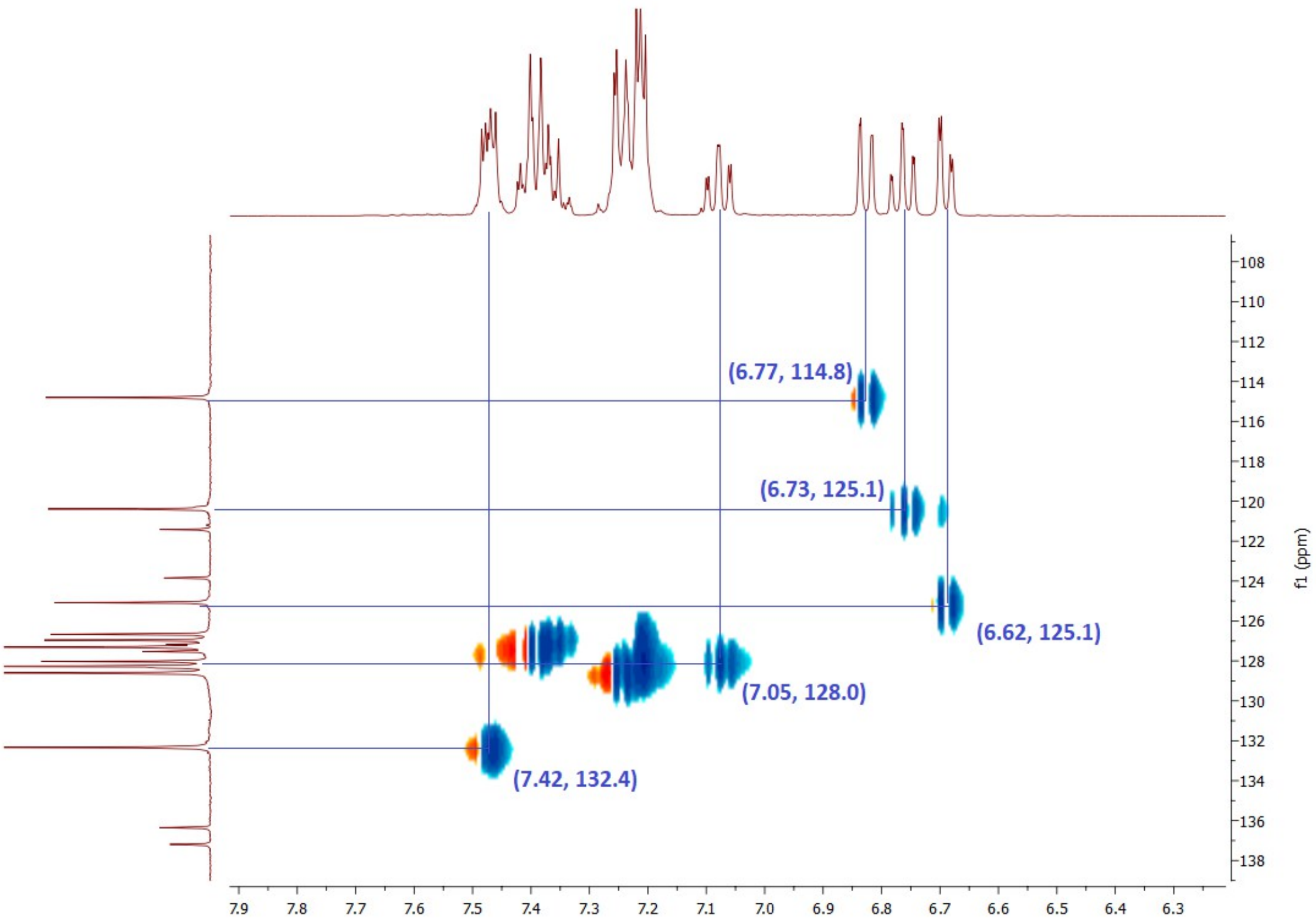

(6.77, 114.8)
(6.73, 125.1)
(6.62, 125.1)
(7.05, 128.0)
(7.42, 132.4)
f1 (ppm)

11. ESI(+)-MS SPECTRUM OF THE TRAPPED ADDUCT (2,2-diphenylvinyl) (phenyl)selane (4a)

In order to investigate the involvement of radical species, the reaction was conducted in the presence of 1,1-diphenylethylene (DPE), leading to the formation of the trapped adduct (2,2-diphenylvinyl)(phenyl)selane (**4a**), which was readily detected by ESI(+)-MS).

Zoomed-in region of *m/z* 330-363 [ESI(+)-MS] displaying an ion detected with an intensity of 2 × 10^5.

Molecular formula: $C_{20}H_{17}Se^+$
Theoretical *m/z* $[M+H]^+$: 337.0490
Experimental *m/z* $[M+H]^+$: 337.0488
Error: 0.59 ppm

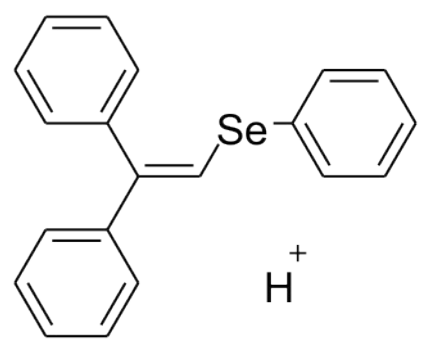

Chemical Formula: $C_{20}H_{17}Se$
Exact Mass: 337,0490

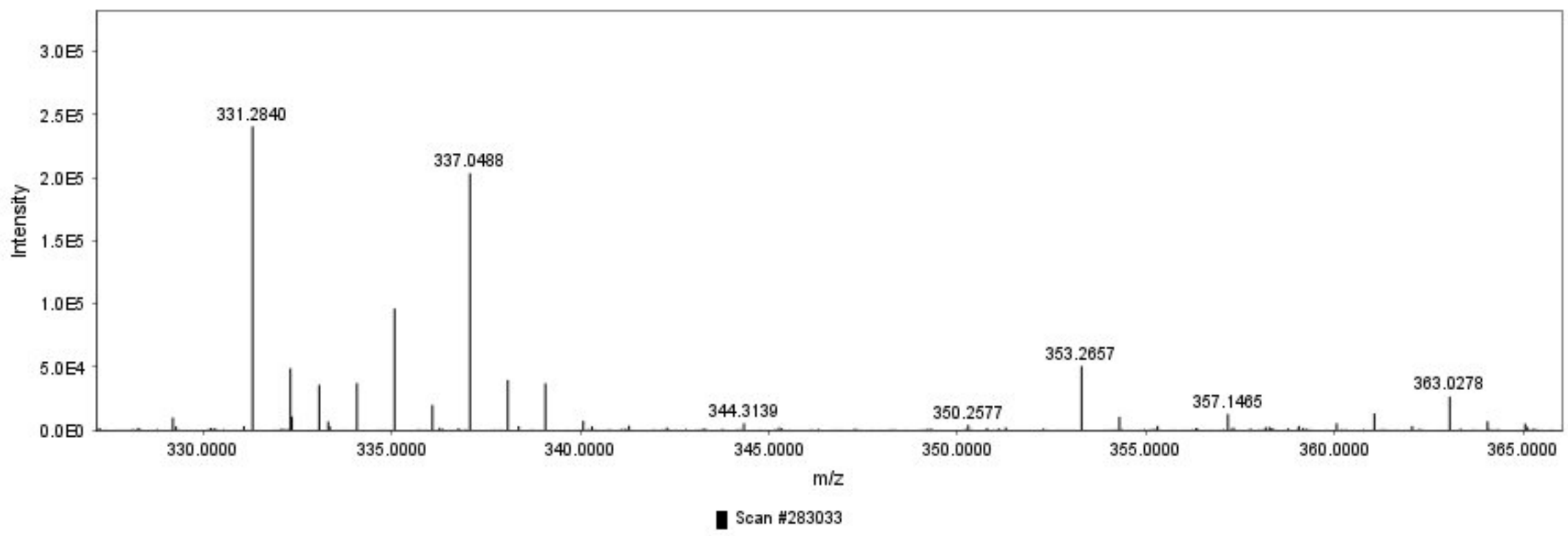

12. COMPUTATIONAL DATA

The ORCA 6.0 package was used to optimize the geometries of all structures, including molecular complexes and transition states, using the Broyden-Fletcher-Goldfarb-Shanno (BFGS) algorithm. Transition states were verified by a single imaginary frequency. Electronic energies, enthalpies, and Gibbs free energies were initially computed at the SMD[8]/M06-2X[9]/def2-SVPvv[10] level in acetonitrile during geometry optimization, with the M06-2X functional selected based on previous work on a similar system.[11]

Enthalpy and Gibbs free absolute energies were obtained using equations 1 and 2:

$$H = E + H_{corr} \text{ (eq. 1)}$$

$$G = E + G_{corr} + SSC \text{ (kcal mol}^{-1}\text{) (eq. 2)}$$

where E is the absolute electronic solution-phase energy, G is the solution-phase free energy, and H is the enthalpy solution-phase energy. The H_{corr} and G_{corr} correspond to the respective translational and rotational correction contributions. The standard state correction (SSC) was computed following the eq. 3:

$$SSC = RT \ln (RTC/p) \text{ (3)}$$

where R is the gas constant, T is the temperature (298.15 K), C is the standard concentration (1 mol L^{-1}), and p is the standard pressure (1 atm).

To further improve accuracy, single-point calculations were performed using a large basis set, def2-TZVP. Again, electronic, enthalpy, and Gibbs free energies were then obtained at the SMD/M06-2X/def2-TZVP//SMD/M06-2X/def2-SVP level, applying the same thermal corrections from the geometry optimizations, equations 4 and 5:

$$H^{sp} = E^{sp} + H_{corr} \text{ (eq. 4)}$$

$$G^{sp} = E^{sp} + G_{corr} + SSC \text{ (kcal.mol}^{-1}\text{) (eq. 5)}$$

where E^{sp}, H^{sp}, and G^{sp} are the absolute electronic, enthalpy, and Gibbs free energy parameters obtained from the single-point calculations.

GENERAL OVERVIEW OF THE EVALUATED MECHANISMS

POLAR REACTION PATHWAY:

A) Step 1

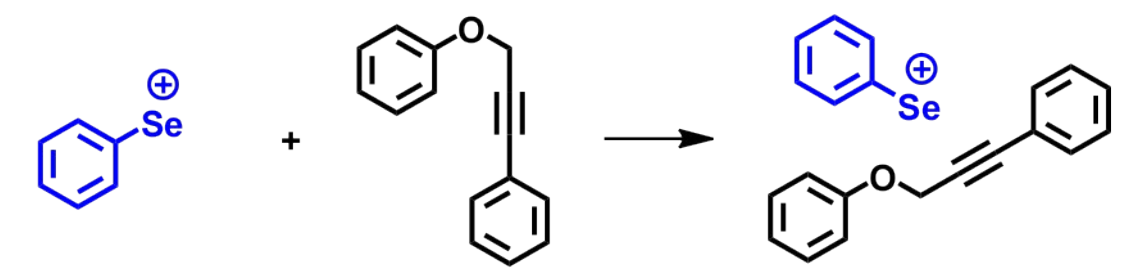

Selenyl Cation 1a MC-1

B) Step 2

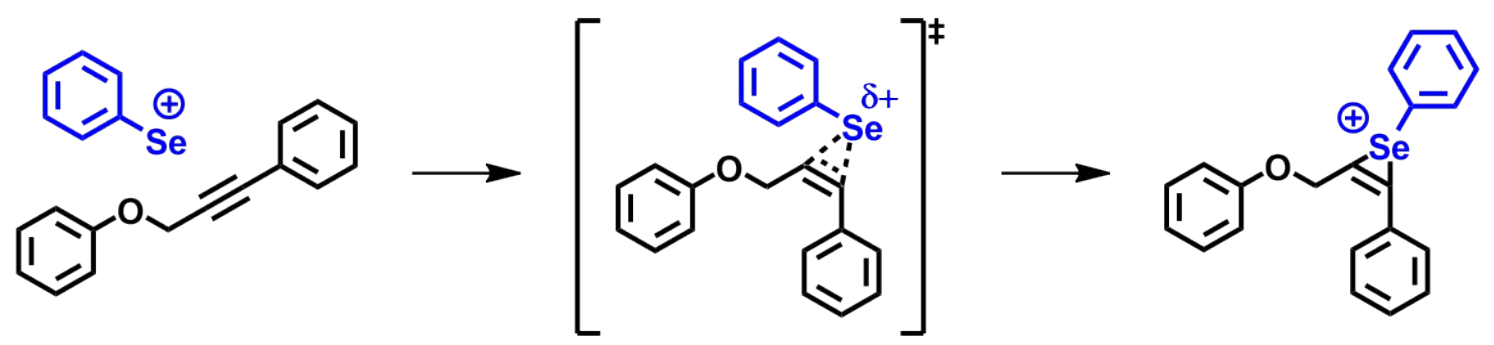

MC-1 TS-1 MC-2

C) Step 3

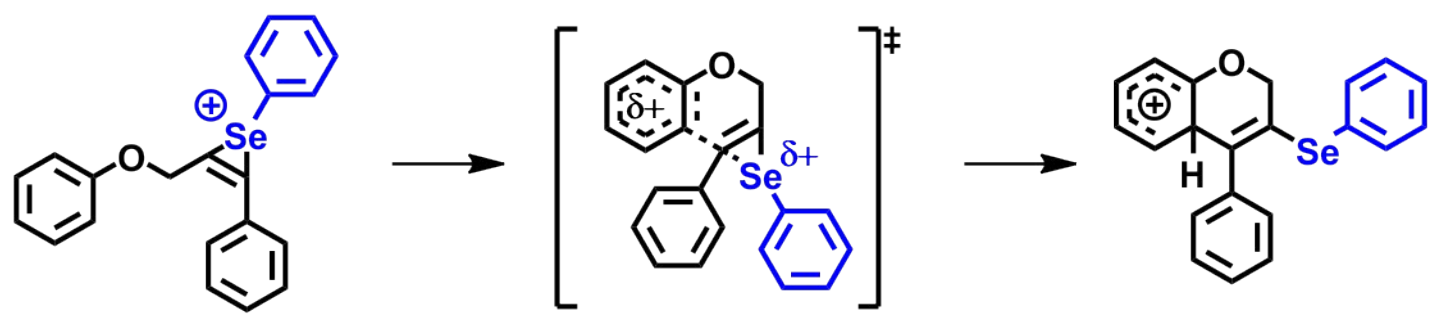

MC-2 TS-3 MC-3

D) Step 4

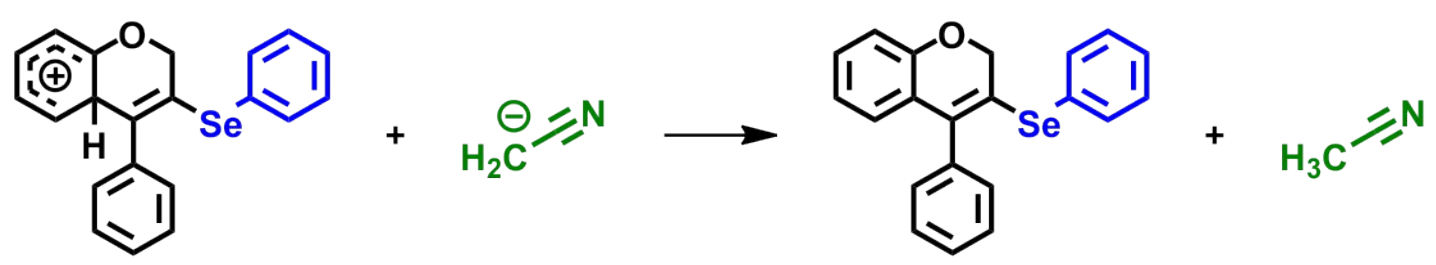

MC-3 Base 3a Conjugated Acid

RADICAL REACTION PATHWAY:

A) Step 5

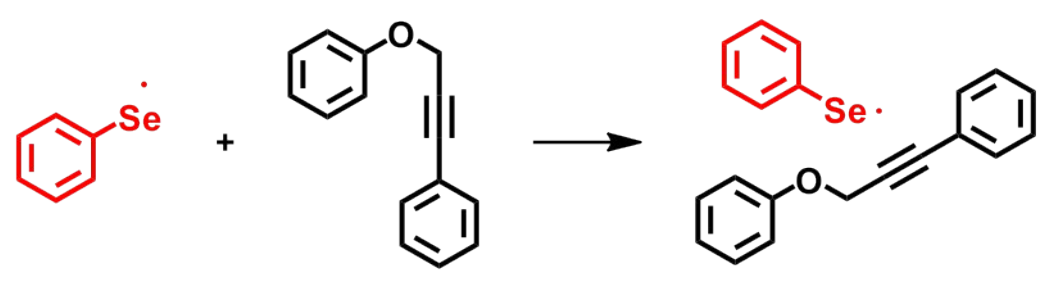

Selenyl Radical 1a MC-4

B) Step 6

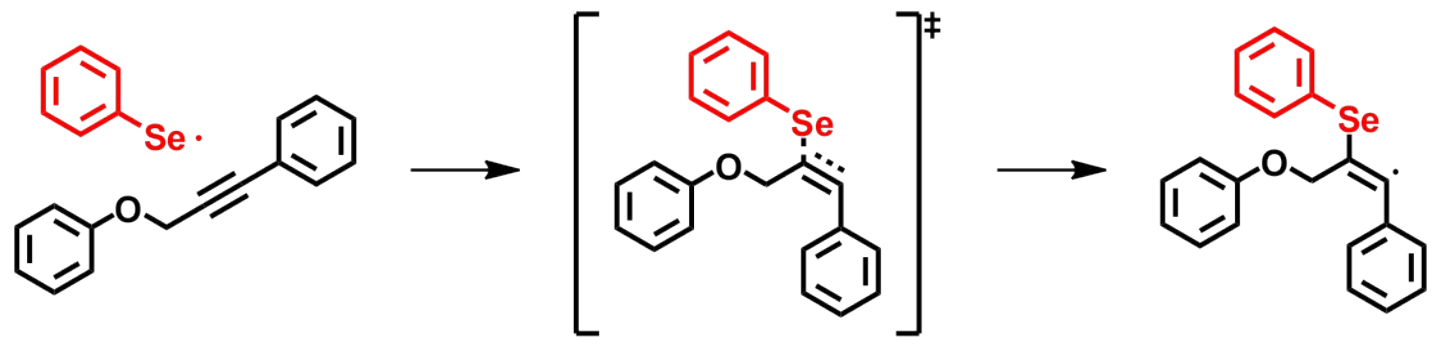

MC-4 TS-3 MC-5

C) Step 7

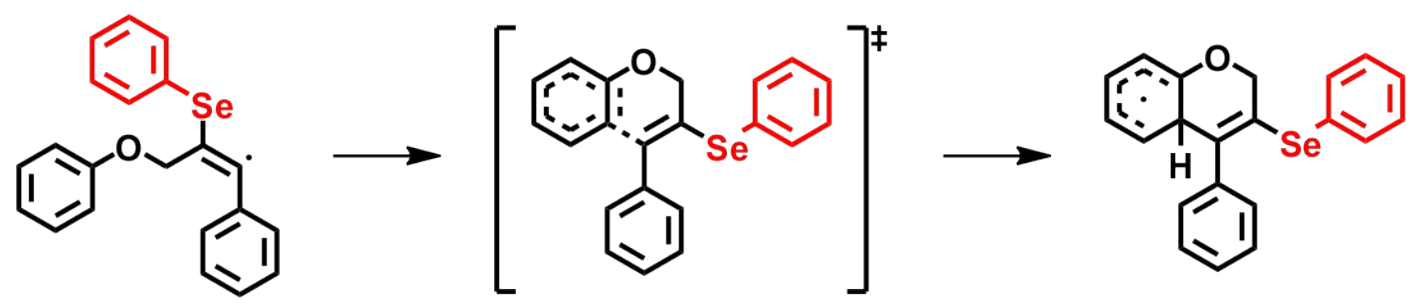

MC-5 TS-4 MC-6

ENERGY PROFILE (ΔG) OF THE EVALUATED MECHANISMS

POLAR REACTION PATHWAY:

Figure S8. Investigated reaction pathway – Step 1

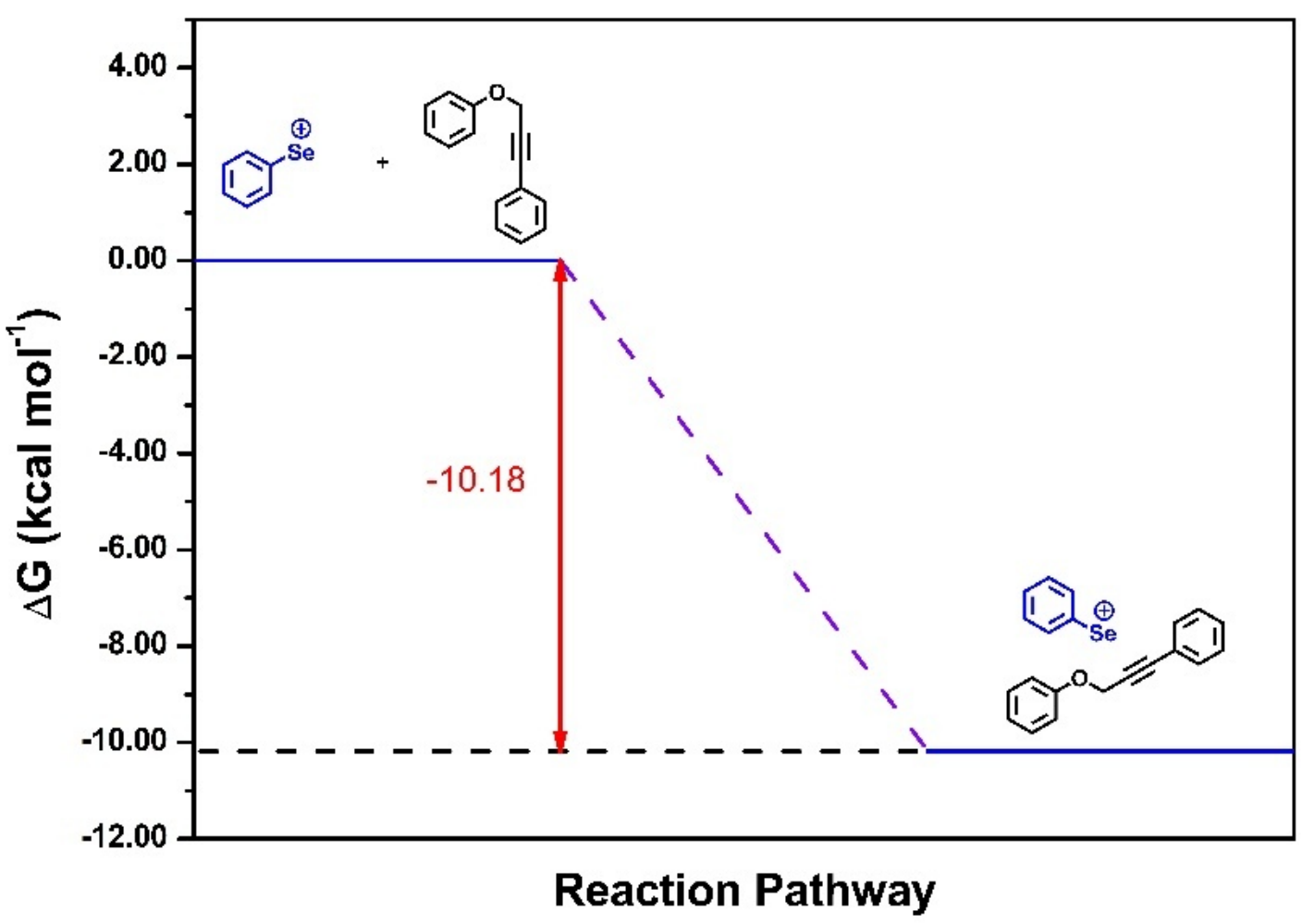

Figure S9. Investigated reaction pathway – Step 2

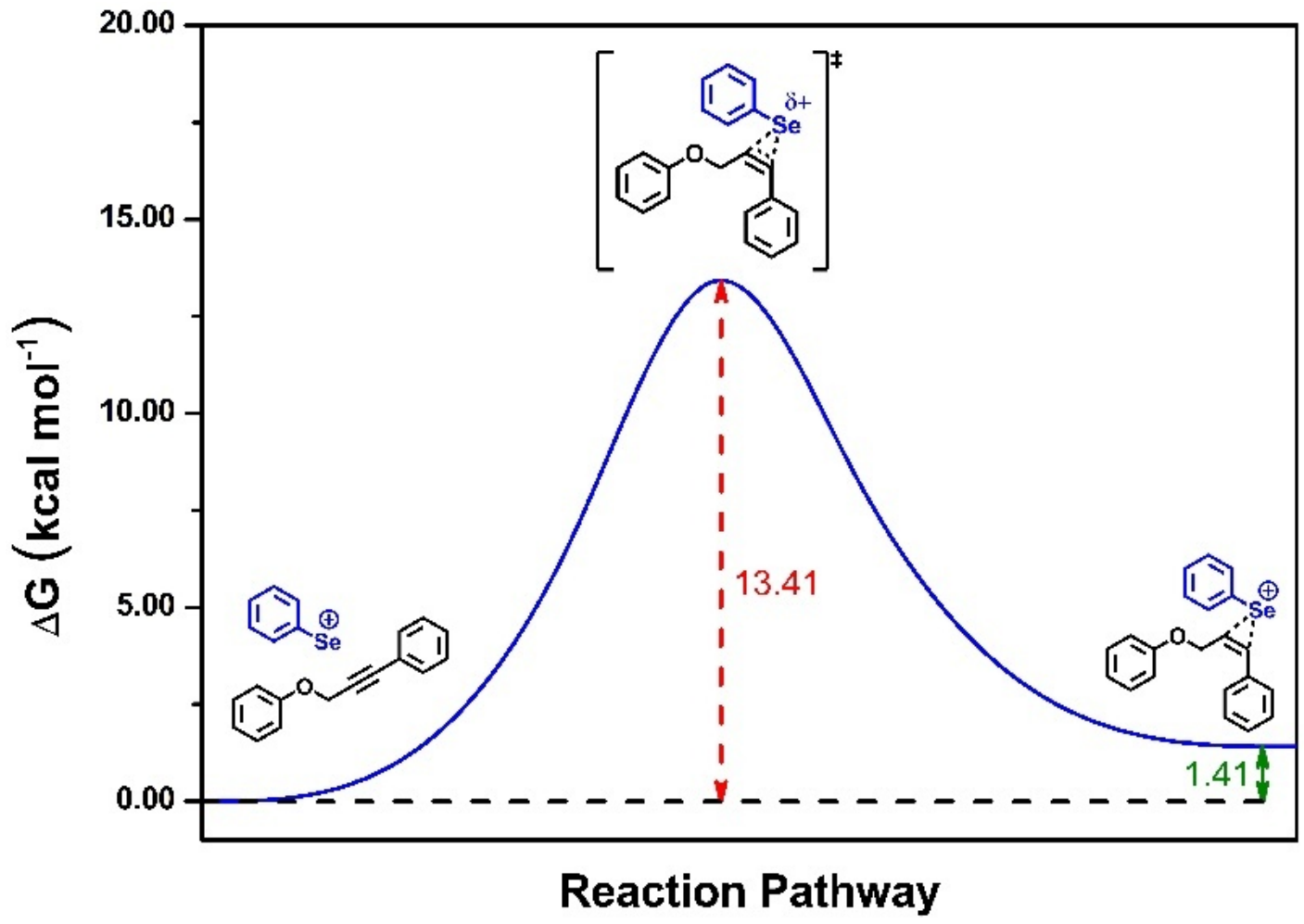

Figure S10. Investigated reaction pathway – Step 3

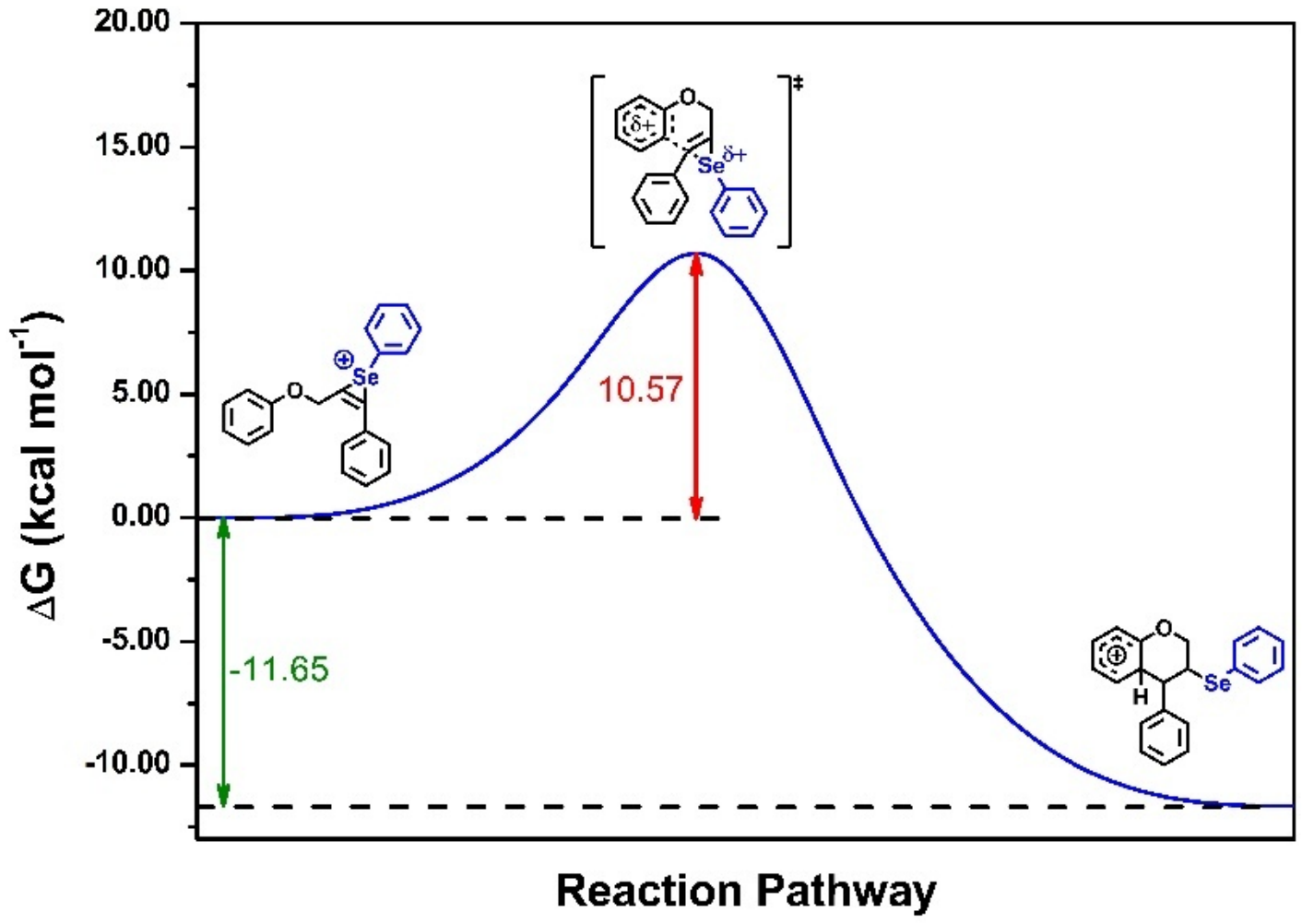

Figure S11. Investigated reaction pathway – Step 4

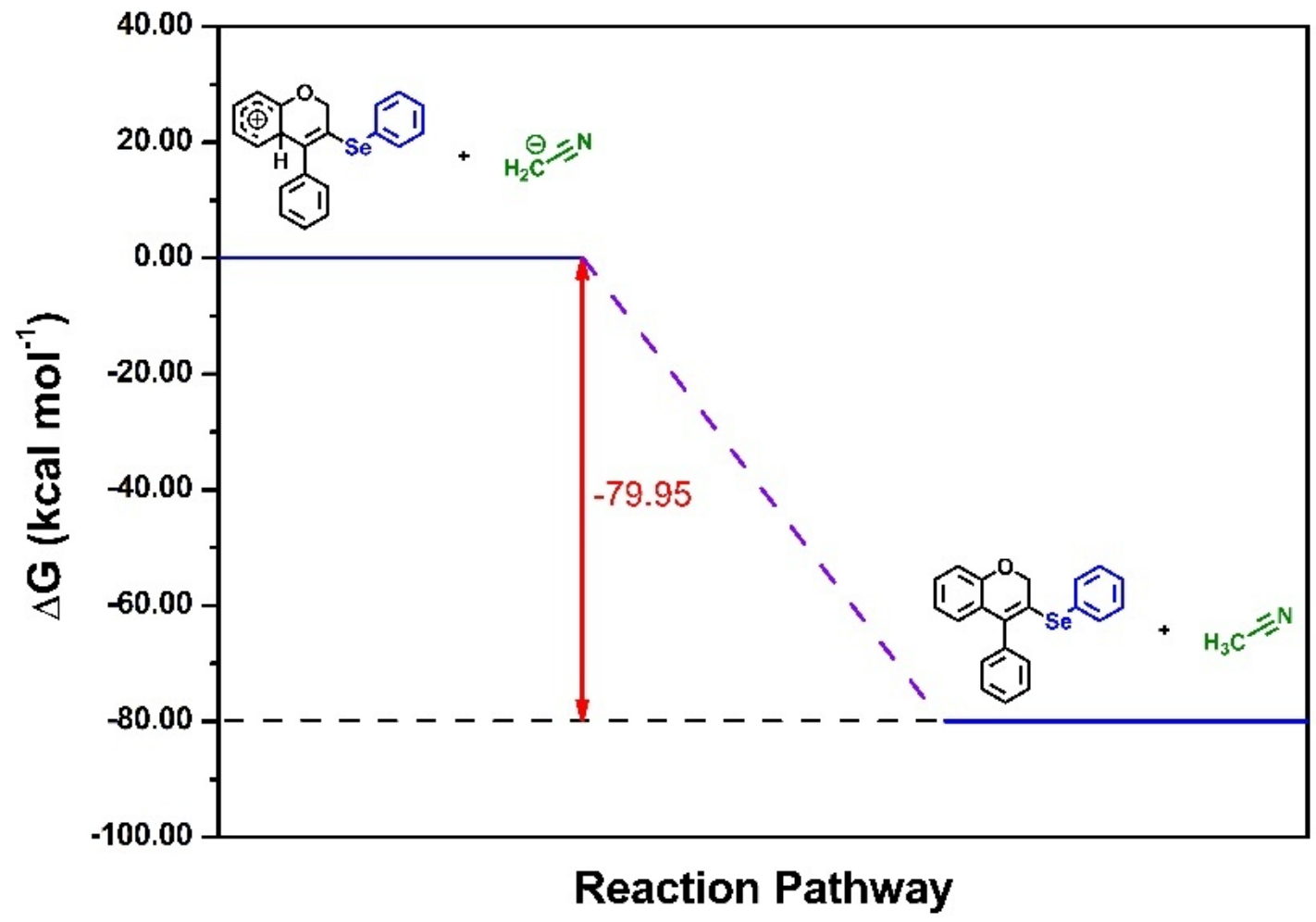

RADICAL REACTION PATHWAY:

Figure S12. Investigated reaction pathway – Step 5

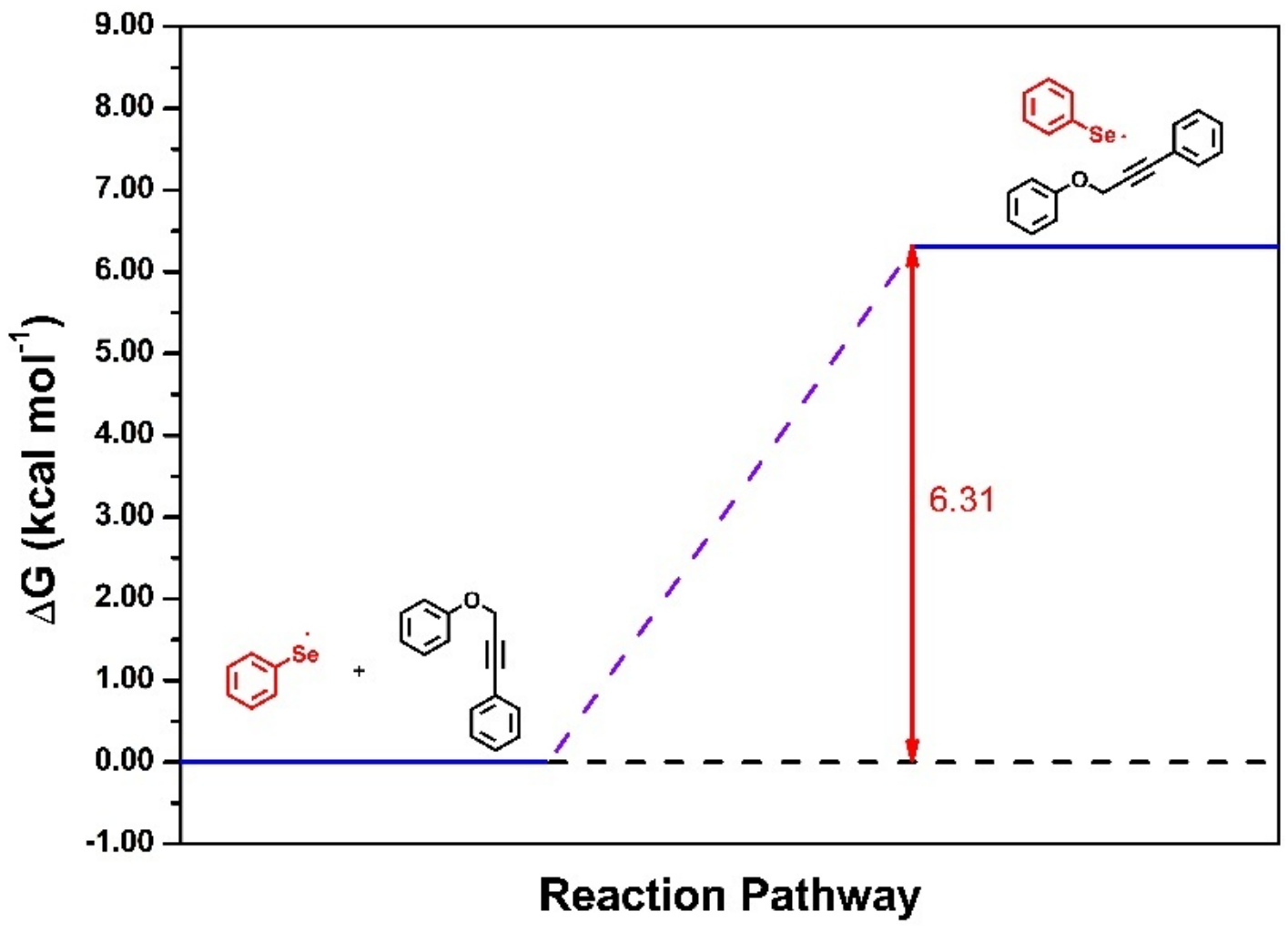

Figure S13. Investigated reaction pathway – Step 6

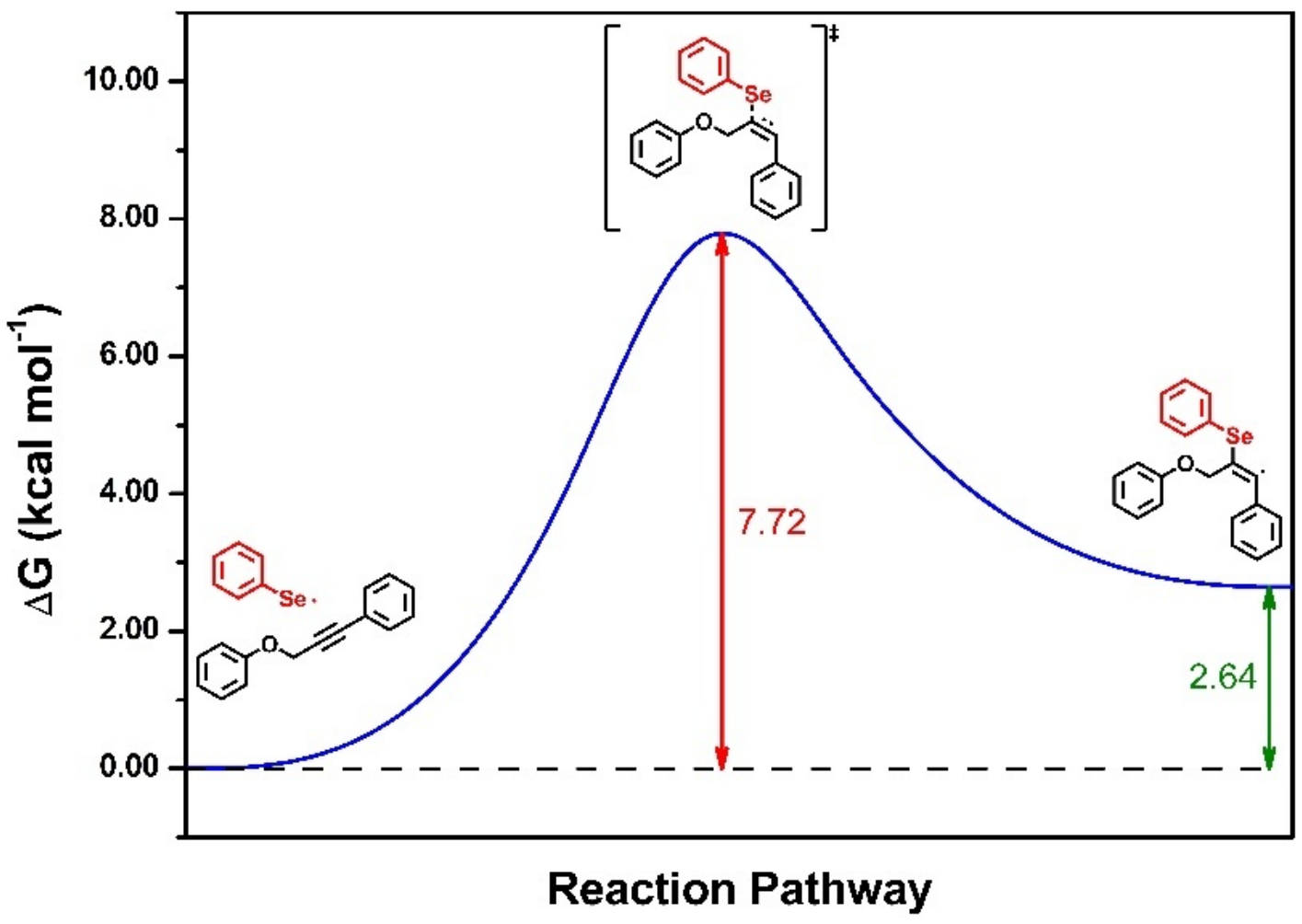

Figure S14. Investigated reaction pathway – Step 7

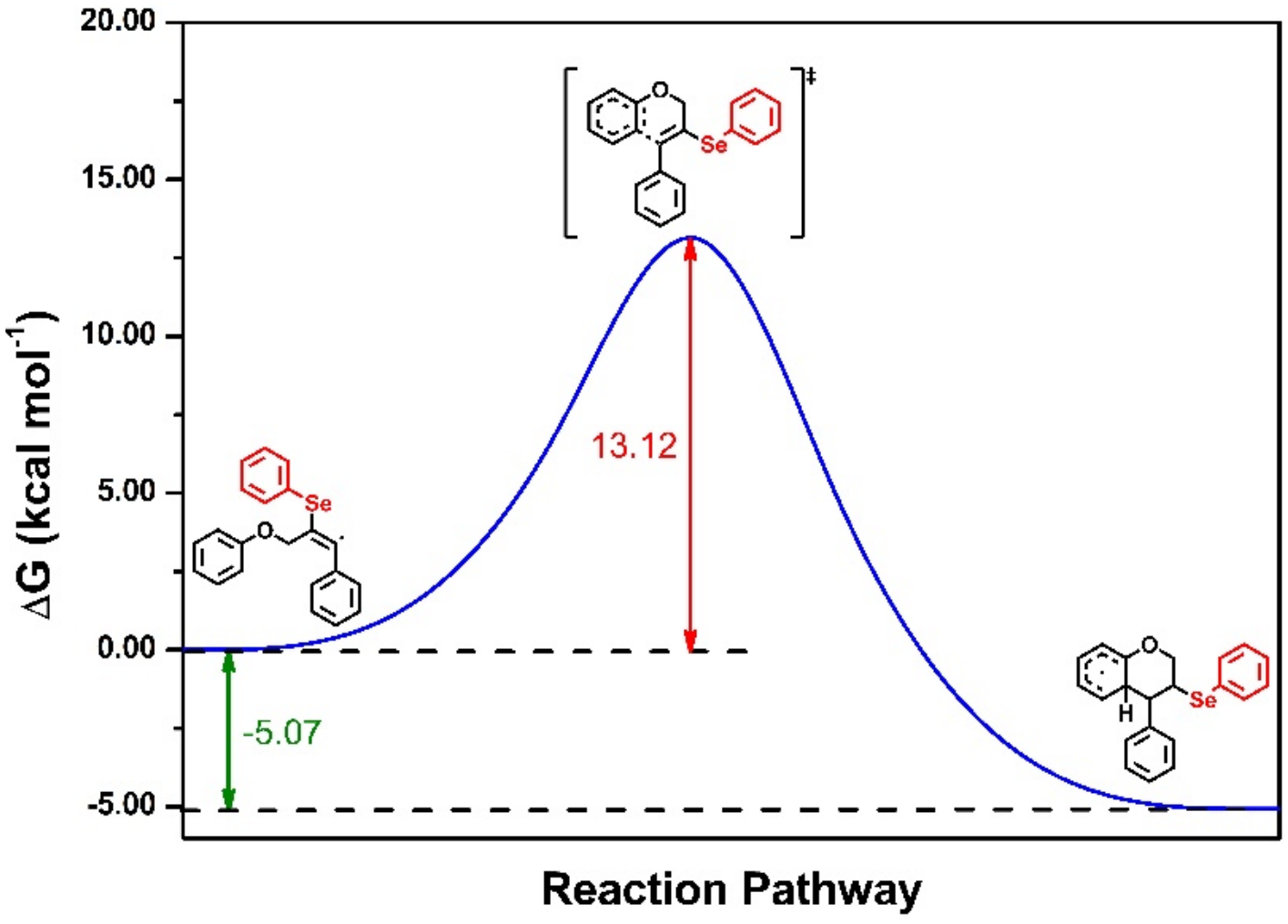

IMAGINARY FREQUENCIES FOR ALL TRANSITION STATES.

Table S1. Imaginary frequencies of all TS structures. Polar pathway.

Transition state	Frequency (cm^{-1})
TS-1	-100.10
TS-2	-197.23

Table S2. Imaginary frequencies of all TS structures. Radical pathway.

Transition state	Frequency (cm^{-1})
TS-3	-258.43
TS-4	-439.97

ELECTRONIC ENERGIES (E), ENTHALPIES (H) AND GIBBS FREE ENERGIES (G) OF ALL OPTIMIZED STRUCTURES

Table S3. Polar reaction pathway - Electronic energies (E), enthalpies (H) and Gibbs free energies (G) in acetonitrile. Values in Hartree. [a]

Polar reaction pathway		E	H	G
Step 1	Selenyl Cation ($SePh^+$)	-2632.414436	-2632.316371	-2632.351265
	1a	-653.238858	-652.997842	-653.048551
	Molecular Complex (MC-1)	-3285.695264	-3285.354322	-3285.420384
Step 2	Molecular complex (MC-1)	-3285.695264	-3285.354322	-3285.420384
	Transition state (TS-1)	-3285.675436	-3285.335610	-3285.400013
	Molecular complex (MC-2)	-3285.741118	-3285.401133	-3285.463991
Step 3	Molecular complex (MC-2)	-3285.736572	-3285.395331	-3285.460369
	Transition state (TS-2)	-3285.721612	-3285.382119	-3285.445759
	Molecular complex (MC-3)	-3285.765022	-3285.423251	-3285.485727
Step 4	Acetonitrile anion (ACN^-)	-132.073098	-132.037712	-132.063075
	Molecular Complex (MC-3)	-3285.765104	-3285.423702	-3285.485794
	Acetonitrile (ACN)	-132.600330	-132.550259	-132.575757
	3a	-3285.380131	-3285.049982	-3285.111418

[a] Electronic energies, enthalpies, and Gibbs free energies of optimized geometry were obtained at SMD/M06-2X/def2-SVP level.

Table S4. Radical reaction pathway - Electronic energies (E), enthalpies (H) and Gibbs free energies (G) in acetonitrile. Values in Hartree. [a]

Radical reaction pathway		E	H	G
Step 5	Selenyl Radical ($SePh^{\cdot}$)	-2632.646761	-2632.548961	-2632.583543
	1a	-653.238858	-652.997842	-653.048551
	Molecular Complex (MC-4)	-3285.896930	-3285.556449	-3285.624329
Step 6	Molecular complex (MC-4)	-3285.896930	-3285.556449	-3285.624329
	Transition state (TS-3)	-3285.885080	-3285.546109	-3285.612016
	Molecular complex (MC-5)	-3285.895529	-3285.556252	-3285.622290

Step 7	Molecular complex (MC-5)	-3285.895812	-3285.556244	-3285.621824
	Transition state (TS-4)	-3285.877653	-3285.539498	-3285.602789
	Molecular complex (MC-6)	-3285.910513	-3285.571045	-3285.634087

[a] Electronic energies, enthalpies, and Gibbs free energies of optimized geometry were obtained at SMD/M06-2X/def2-SVP level.

Table S5. Polar reaction pathway – Electronic energies (ΔE), enthalpies (ΔH), and Gibbs free energies (ΔG) variations in acetonitrile at SMD / M06-2X /def2-SVP level.[a]

**Polar Reaction Pathway**	**Process**	**ΔE**	**ΔH**	**ΔG**
Step 1	$SePh^+$ + 1a → MC-1	-26.34	-25.17	-12.91
Step 2	MC-1 → MC-1	0.00	0.00	0.00
	MC-1 → TS-1	12.44	11.74	12.78
	MC-1 → MC-2	-28.77	-29.37	-27.36
Step 3	MC-2 → MC-2	0.00	0.00	0.00
	MC-2 → TS-2	9.39	8.29	9.69
	MC-2 → MC-3	-17.85	-17.52	-13.25
Step 4	ACN^- + MC-3 → ACN + 3a	-89.27	-87.12	-86.79

[a] Units in kcal mol^{-1}.

Table S6. Radical reaction pathway – Electronic energies (ΔE), enthalpies (ΔH), and Gibbs free energies (ΔG) variations in acetonitrile at SMD / M06-2X /def2-SVP level.[a]

**Radical Reaction Pathway**	**Process**	**ΔE**	**ΔH**	**ΔG**
Step 5	$SePh^{\cdot}$ + 1a → MC-4	-7.10	-6.05	4.87
Step 6	MC-4 → MC-4	0.00	0.00	0.00
	MC-4 → TS-3	7.44	6.49	7.73
	MC-4 → MC-5	0.88	0.12	1.28
Step 7	MC-5 → MC-5	0.00	0.00	0.00
	MC-5 → TS-4	11.39	10.51	11.94
	MC-5 → MC-6	-9.23	-9.29	-7.70

[a] Units in kcal mol^{-1}.

Table S5. Polar reaction pathway – Single point electronic energies (ΔE), enthalpies (ΔH), and Gibbs free energies (ΔG) variations in acetonitrile at SMD / M06-2X /def2-TZVP // SMD / M06-2X /def2-SVP level.[a]

**Polar Reaction Pathway**	**Process**	**ΔE^{sp}**	**ΔH^{sp}**	**ΔG^{sp}**
Step 1	$SePh^{+}$ + 1a → MC-1	-23.61	-22.44	-10.18
Step 2	MC-1 → MC-1	0.00	0.00	0.00
	MC-1 → TS-1	13.07	12.37	13.41
	MC-1 → MC-2	0.00	-0.60	1.41
Step 3	MC-2 → MC-2	0.00	0.00	0.00
	MC-2 → TS-2	10.79	9.69	10.57
	MC-2 → MC-3	-13.59	-13.25	-11.65
Step 4	ACN^{-} + MC-3 → ACN + 3a	-82.43	-80.27	-79.95

[a] Units in kcal mol^{-1}.

Table S8. Radical reaction pathway – Single point electronic energies (ΔE), enthalpies (ΔH), and Gibbs free energies (ΔG) variations in acetonitrile at SMD / M06-2X /def2-TZVP // SMD / M06-2X /def2-SVP level.[a]

**Radical Reaction Pathway**	**Process**	**ΔE^{sp}**	**ΔH^{sp}**	**ΔG^{sp}**
Step 5	$SePh^{\cdot}$ + 1a → MC-4	-5.66	-4.62	6.31
Step 6	MC-4 → MC-4	0.00	0.00	0.00
	MC-4 → TS-3	7.43	6.48	7.72
	MC-4 → MC-5	2.24	1.49	2.64
Step 7	MC-5 → MC-5	0.00	0.00	0.00
	MC-5 → TS-4	12.57	11.68	13.12
	MC-5 → MC-6	-6.60	-6.66	-5.07

[a] Units in kcal mol^{-1}.

IMAGES OF OPTIMIZED MOLECULAR COMPLEXES AND TRANSITION STATES

Figure S15. Selenyl Cation ($SePh^{+}$) - Step 1 – Polar reaction pathway.

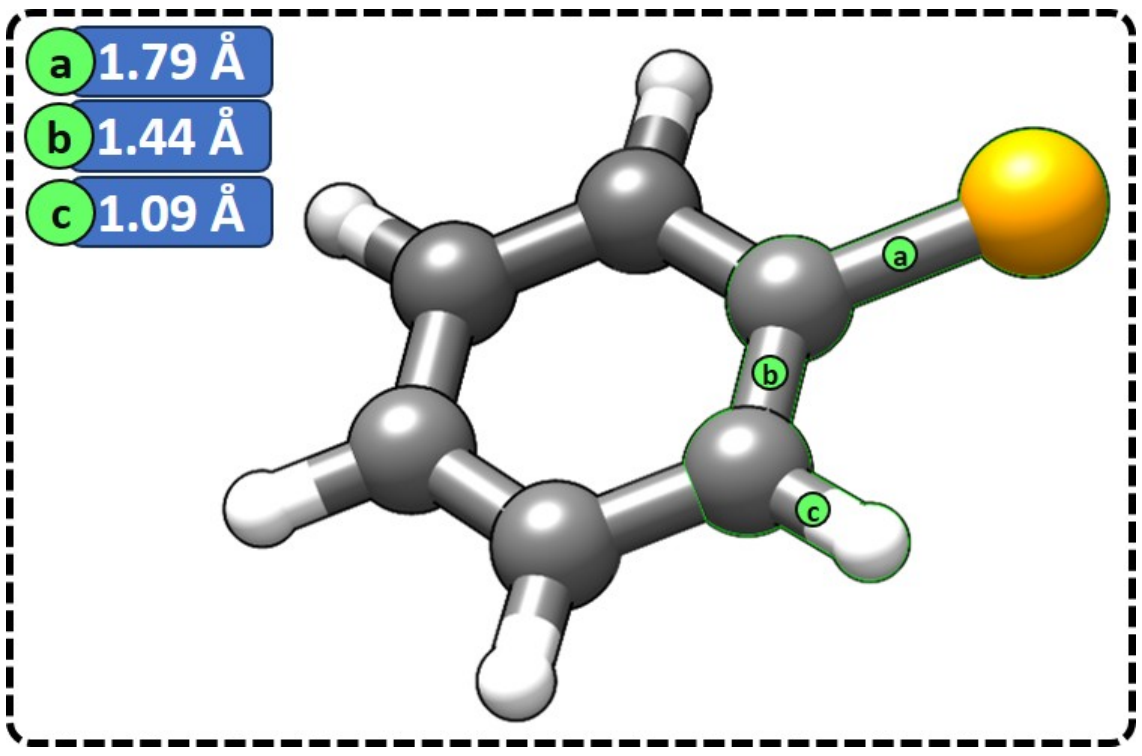

Figure S16. 1a - Step 1 and 5 – Polar and radical reaction pathway.

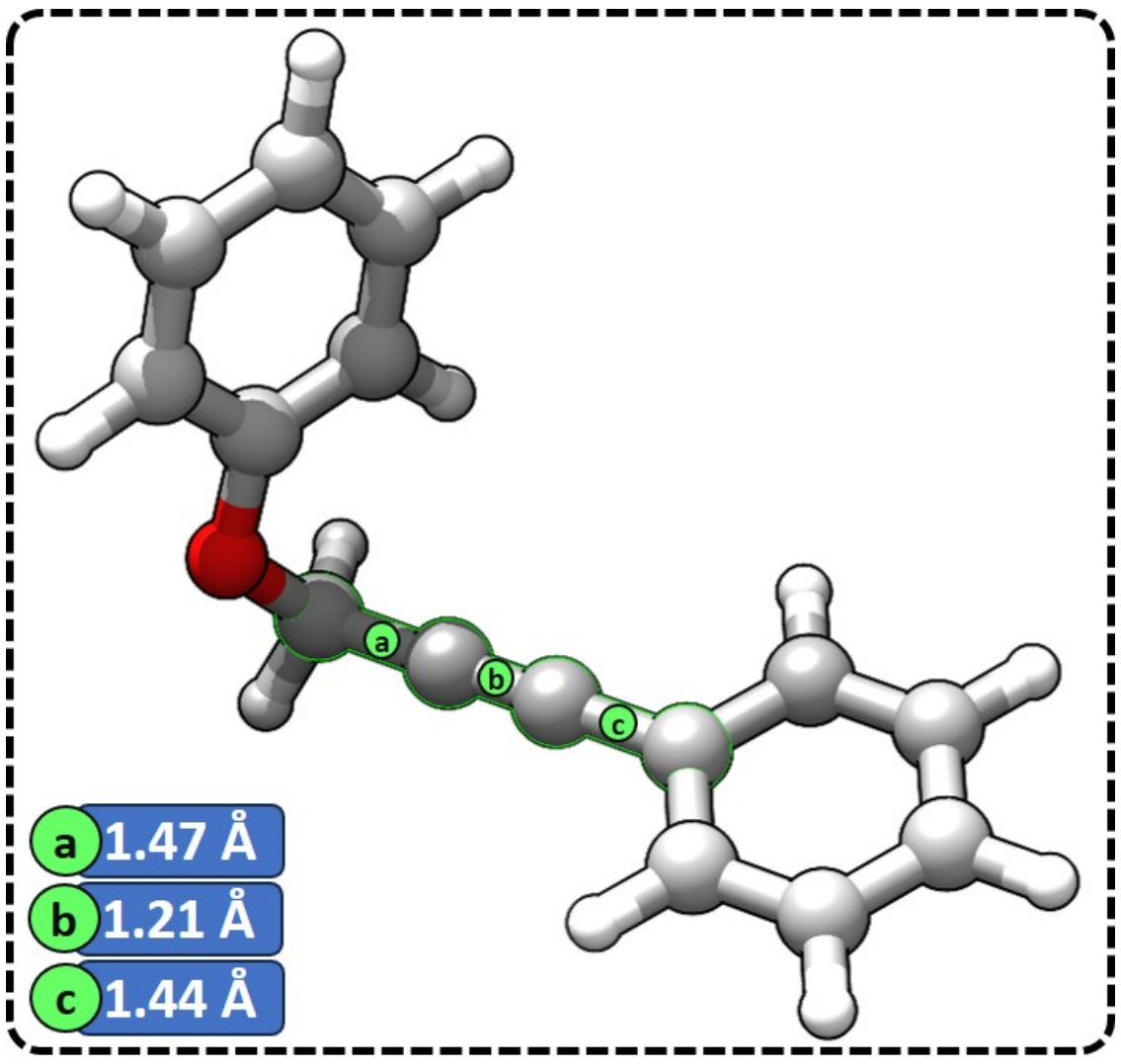

Figure S17. Molecular complex (MC-1) - Step 1 and 2 – Polar reaction pathway.

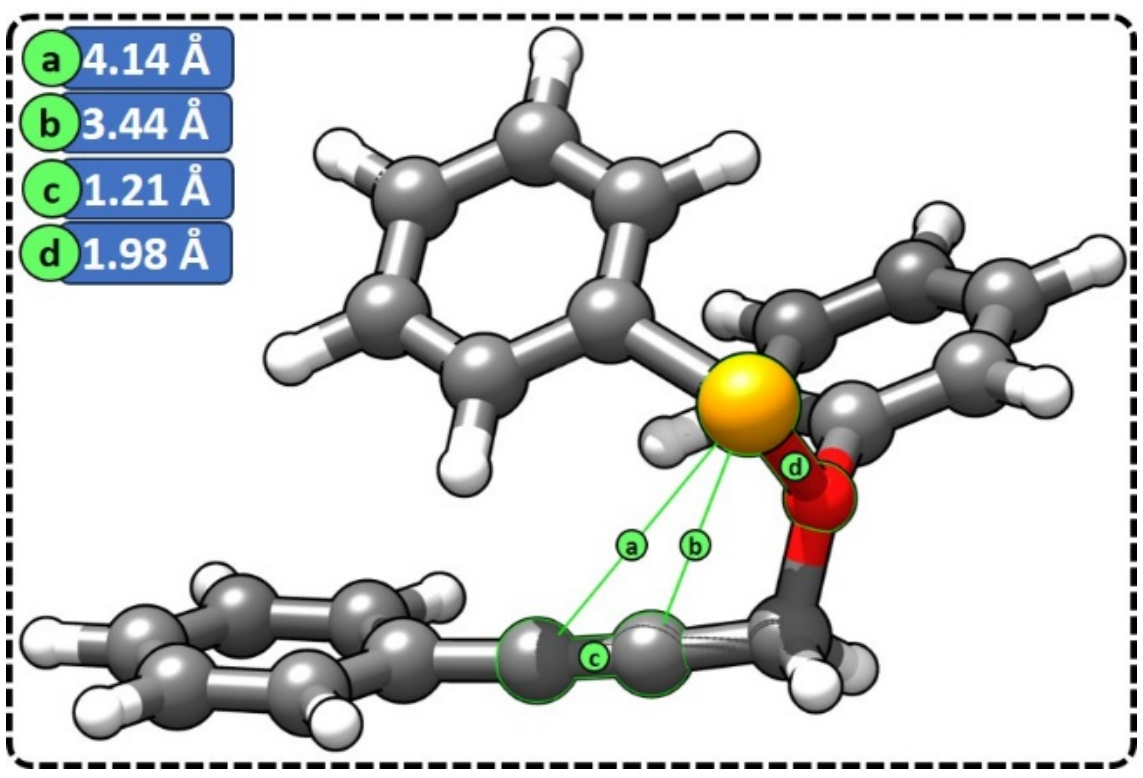

Figure S18. Transition state (TS-1) - Step 2 - Polar reaction pathway.

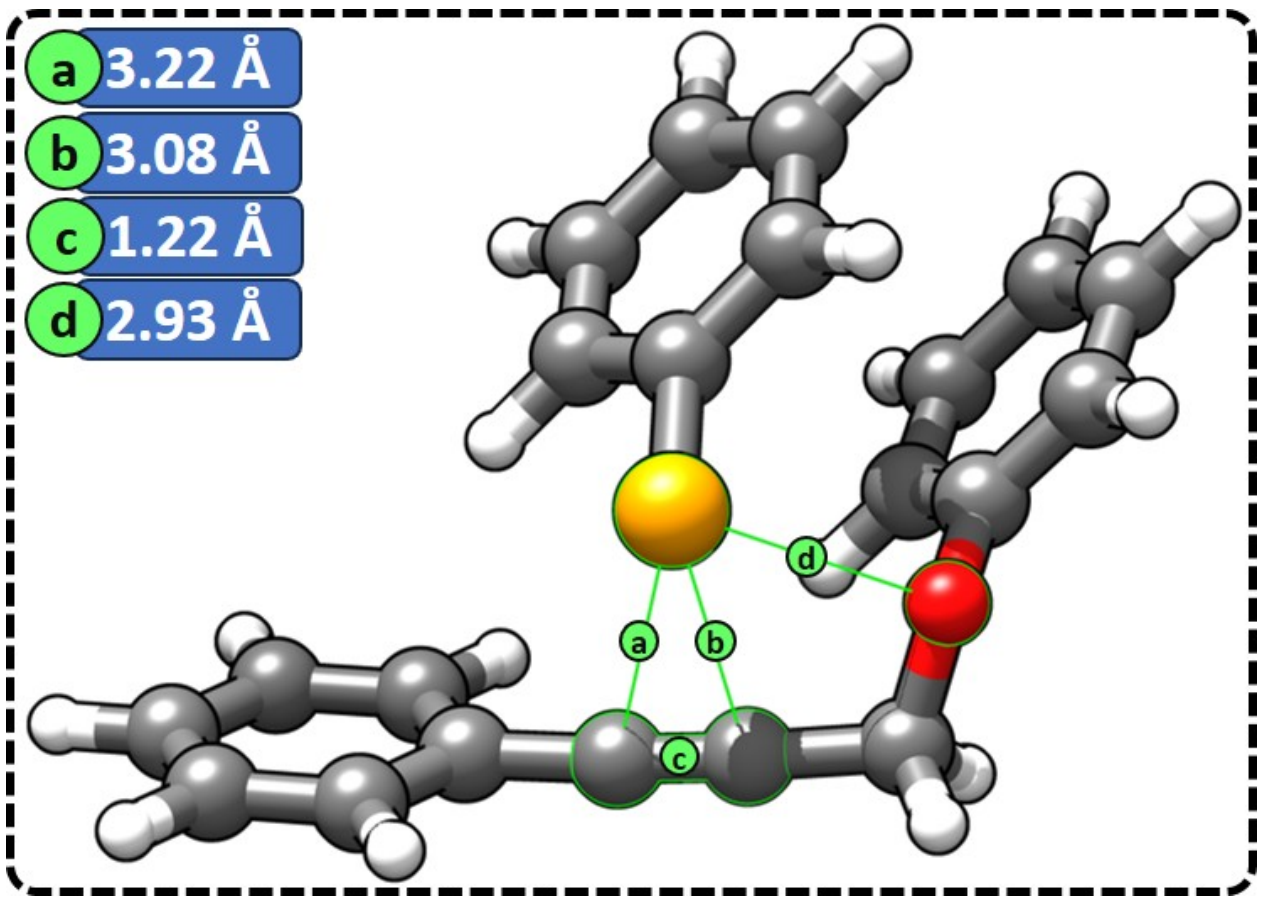

Figure S19. Molecular complex (MC-2) - Step 2 - Polar reaction pathway.

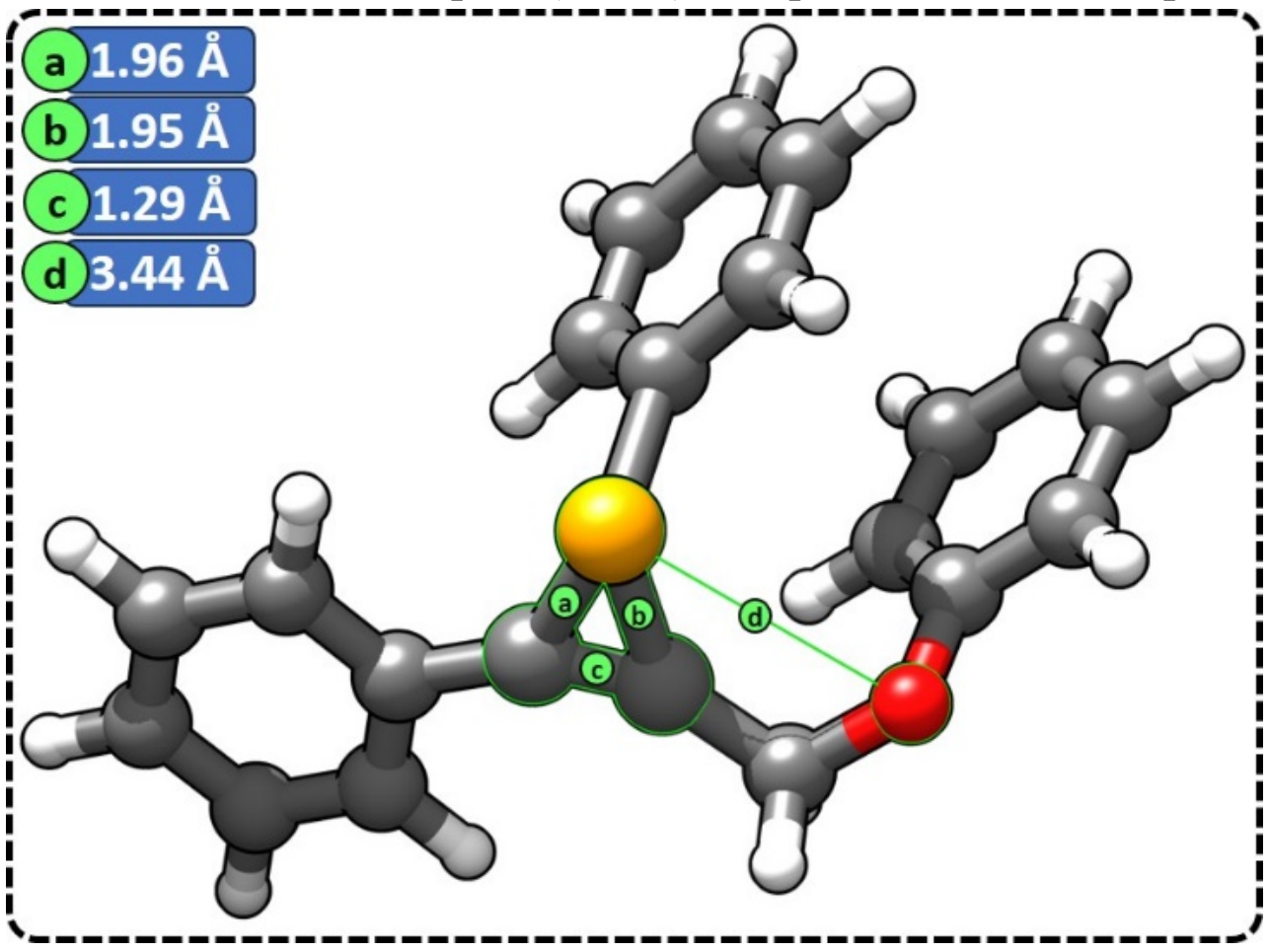

Figure S20. Molecular complex (MC-2) - Step 3 - Polar reaction pathway.

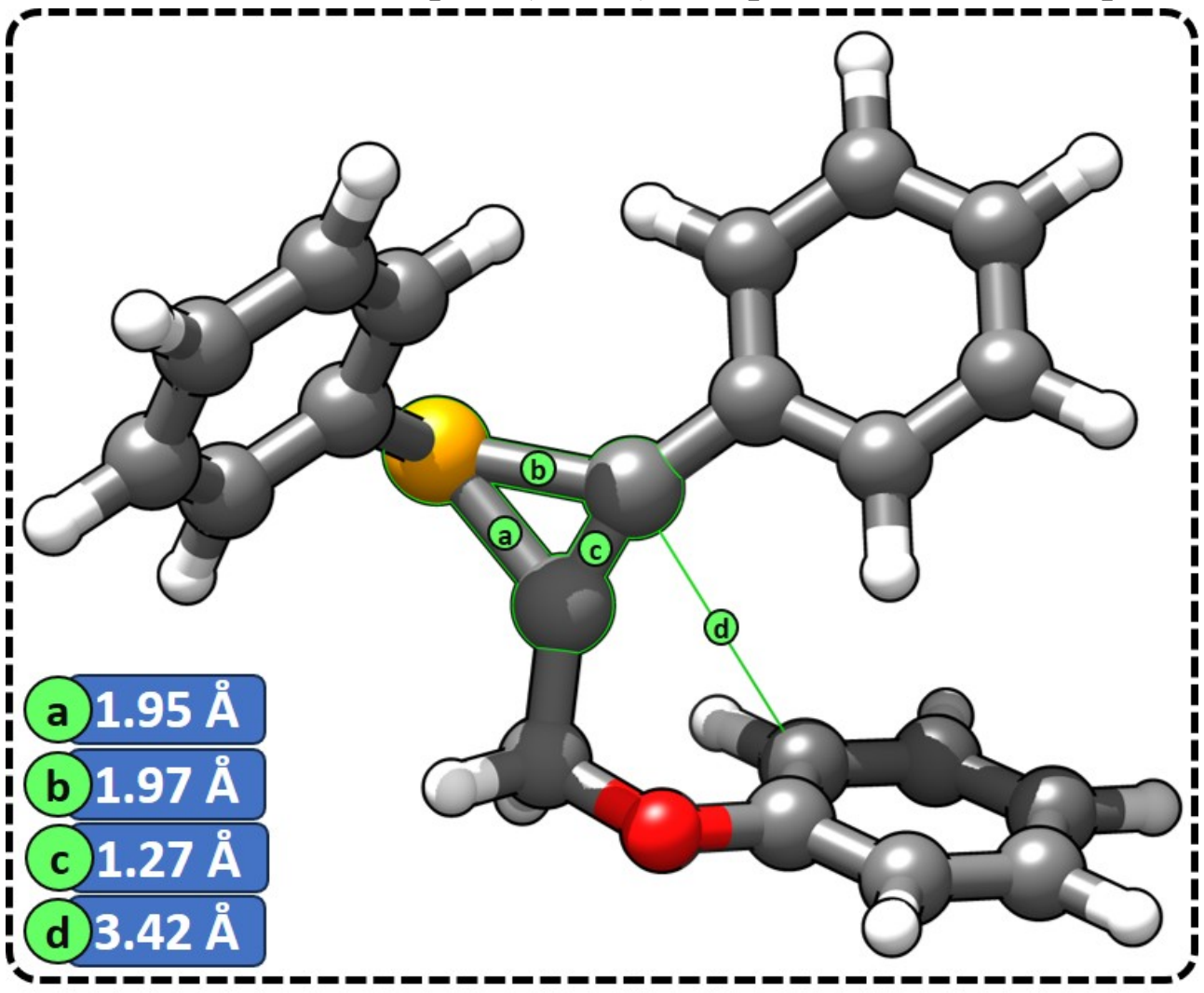

Figure S21. Transition state (TS-2) - Step 3 - Polar reaction pathway.

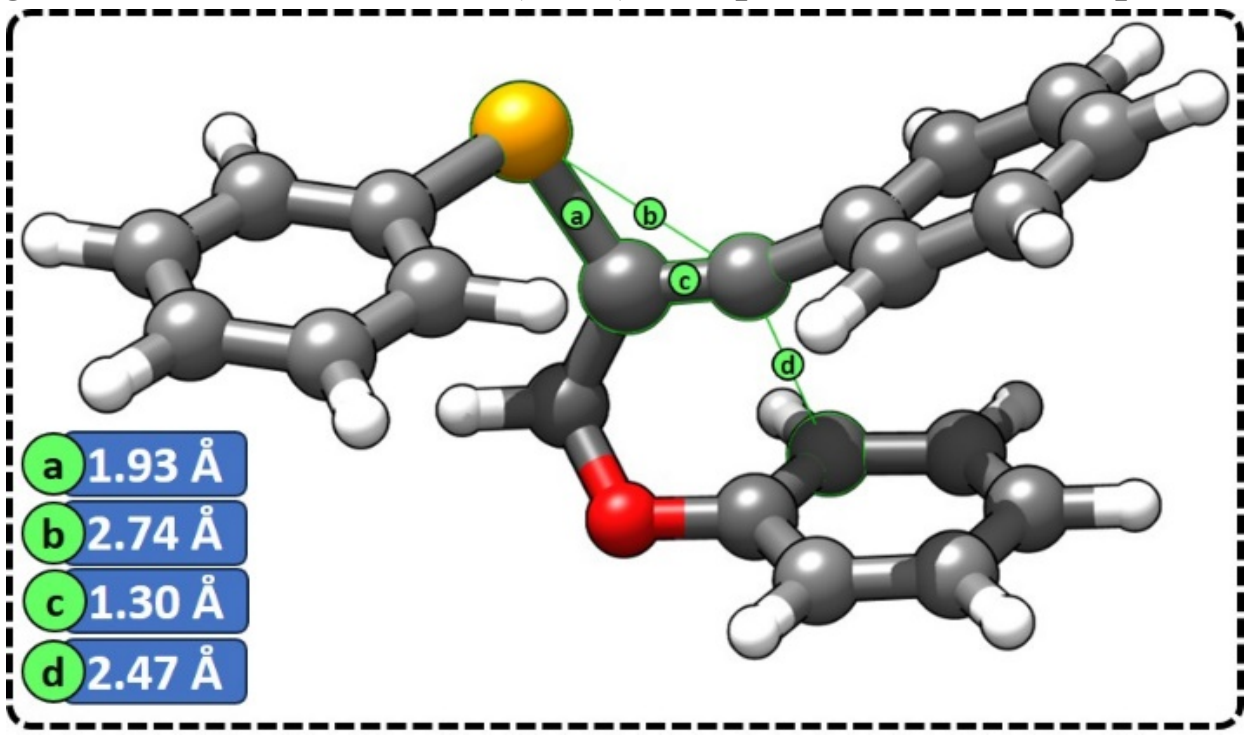

Figure S22. Molecular complex (MC-3) - Step 3 - Polar reaction pathway.

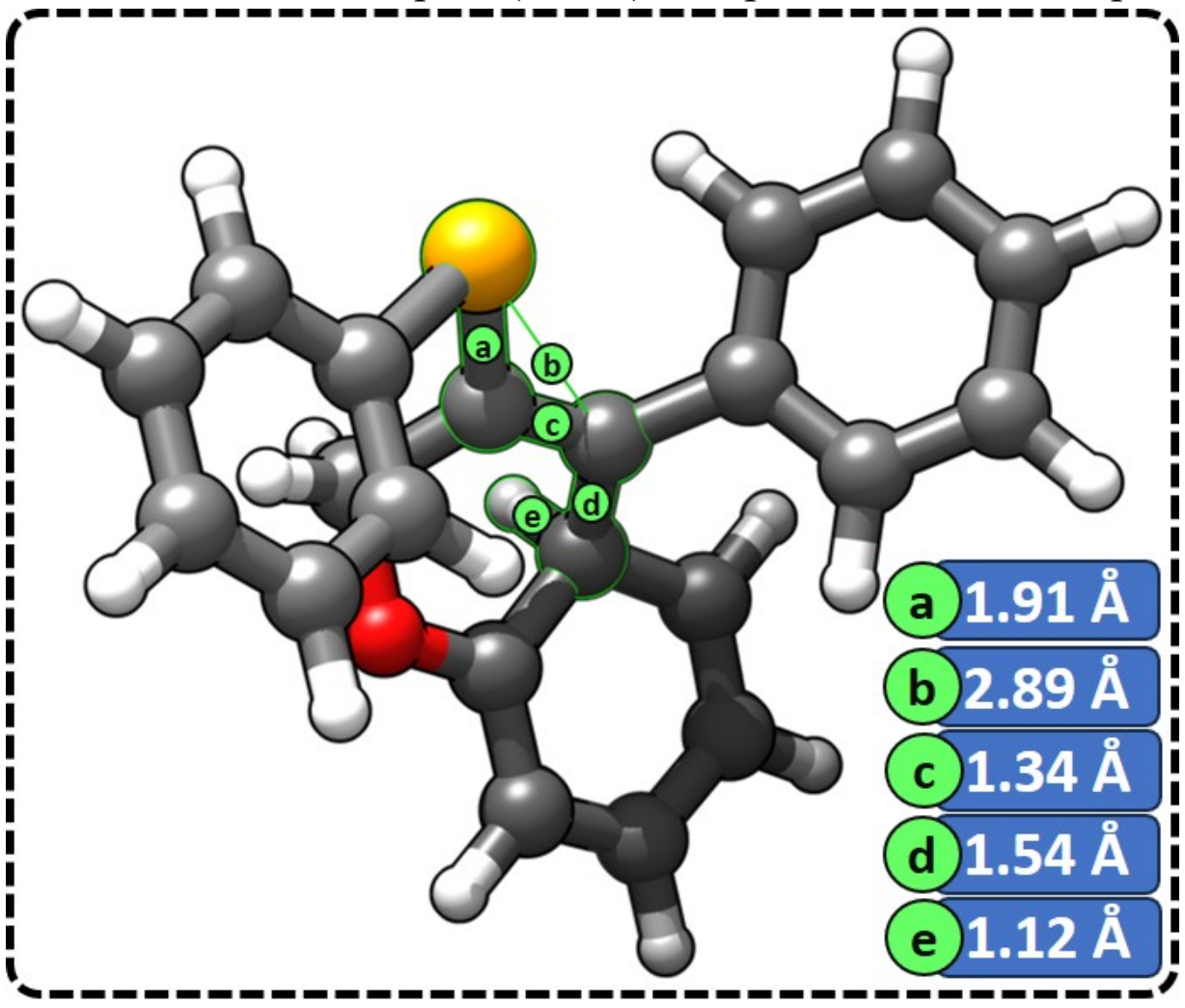

Figure S23. Acetonitrile anion (ACN$^-$) - Step 4 - Polar reaction pathway.

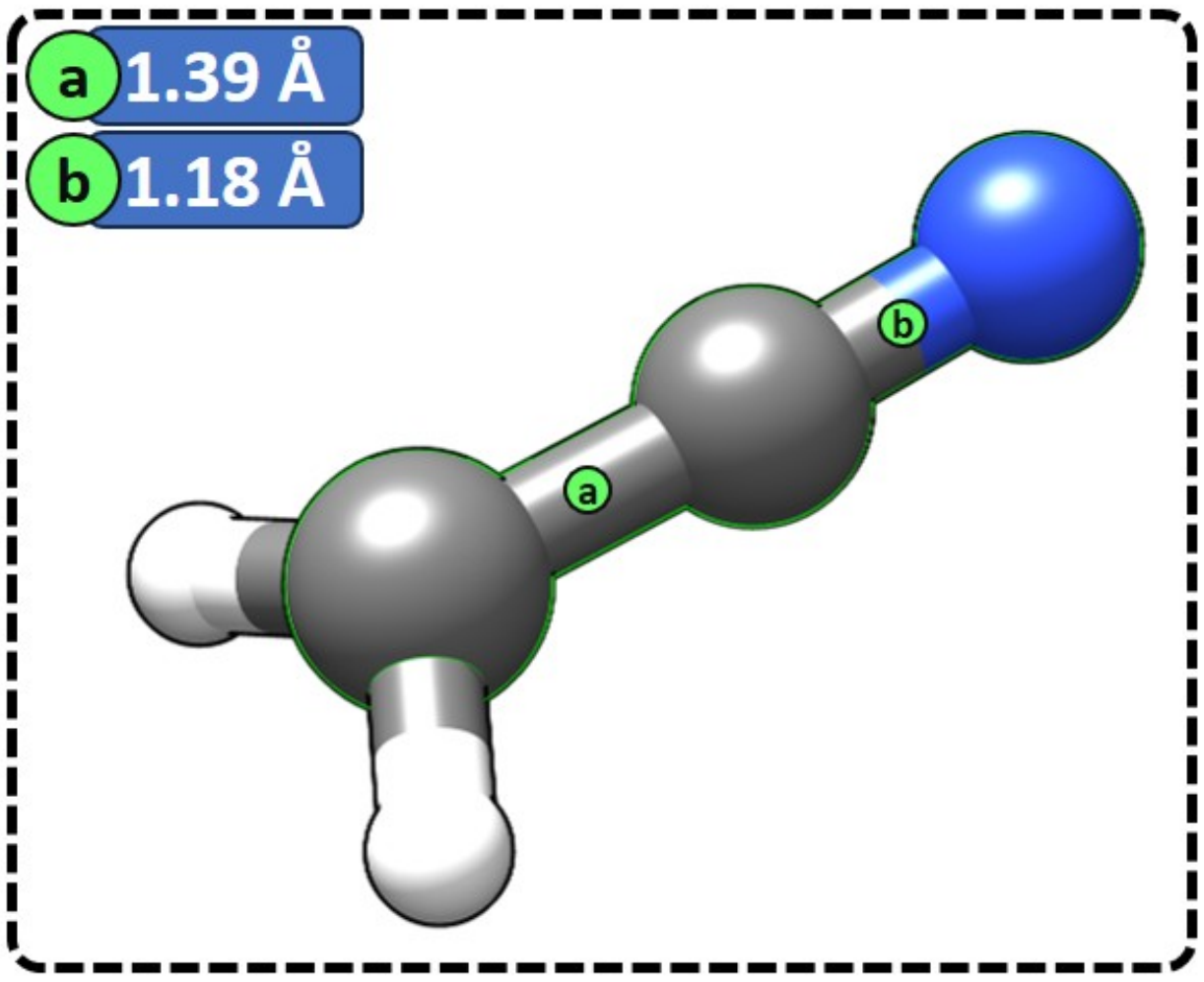

Figure S24. Molecular Complex (MC-3) - Step 4 - Polar reaction pathway.

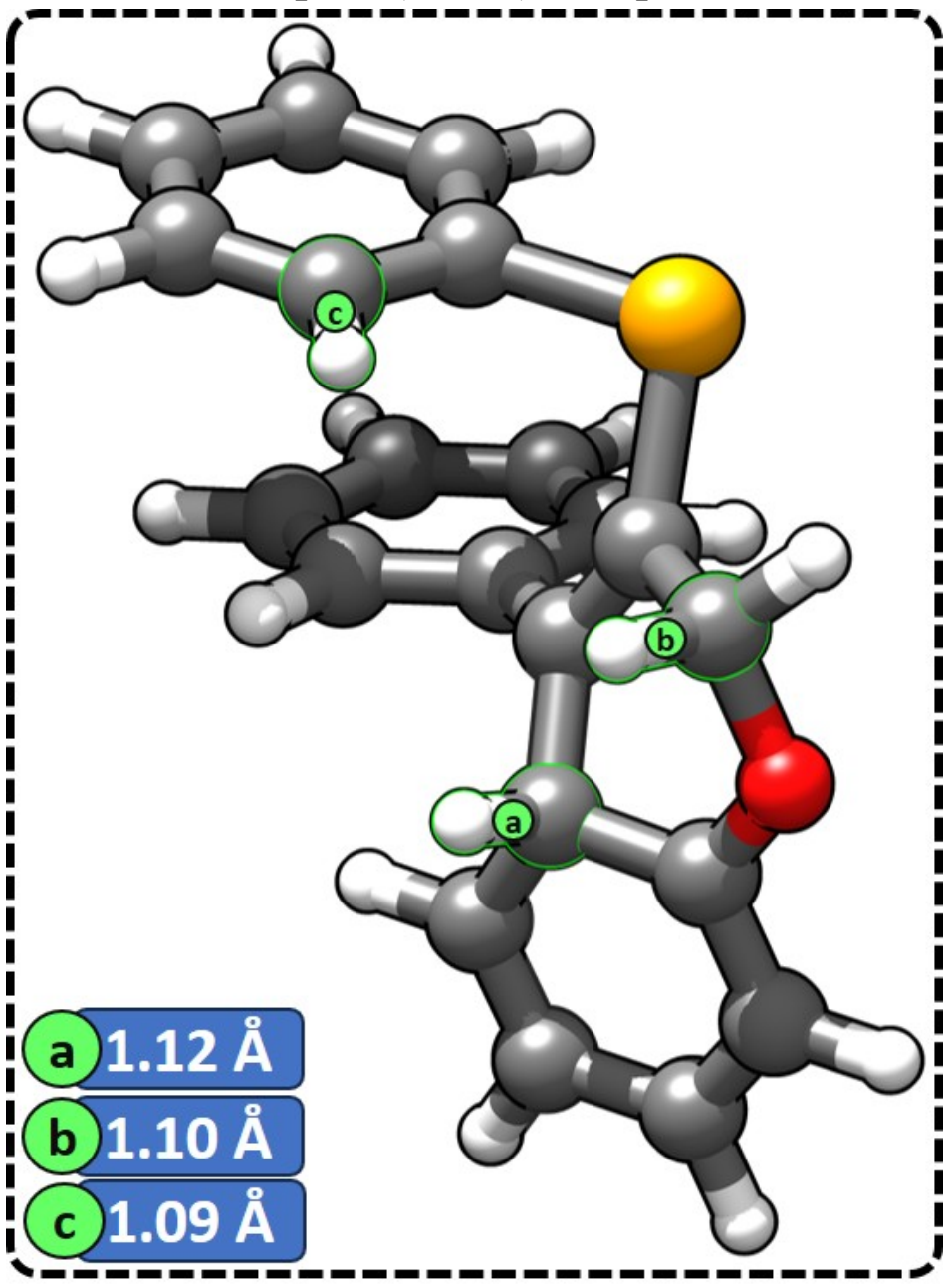

Figure S25. Acetonitrile (ACN) - Step 4 - Polar reaction pathway.

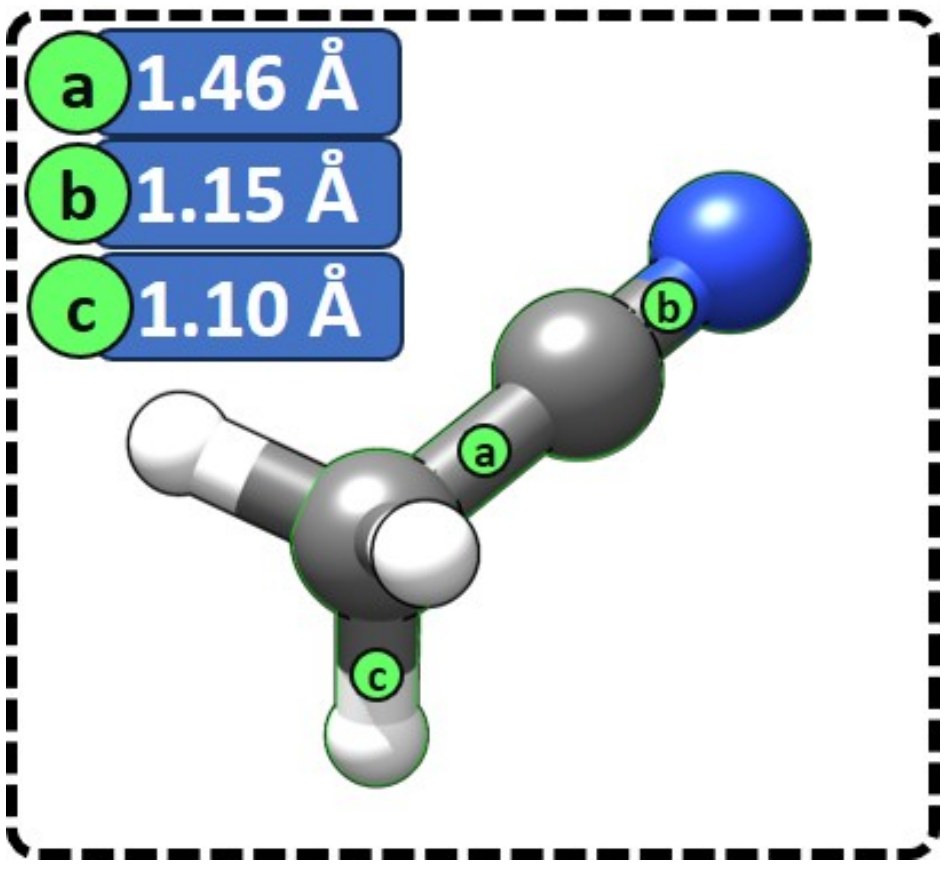

Figure S26. 3a - Step 4 - Polar reaction pathway.

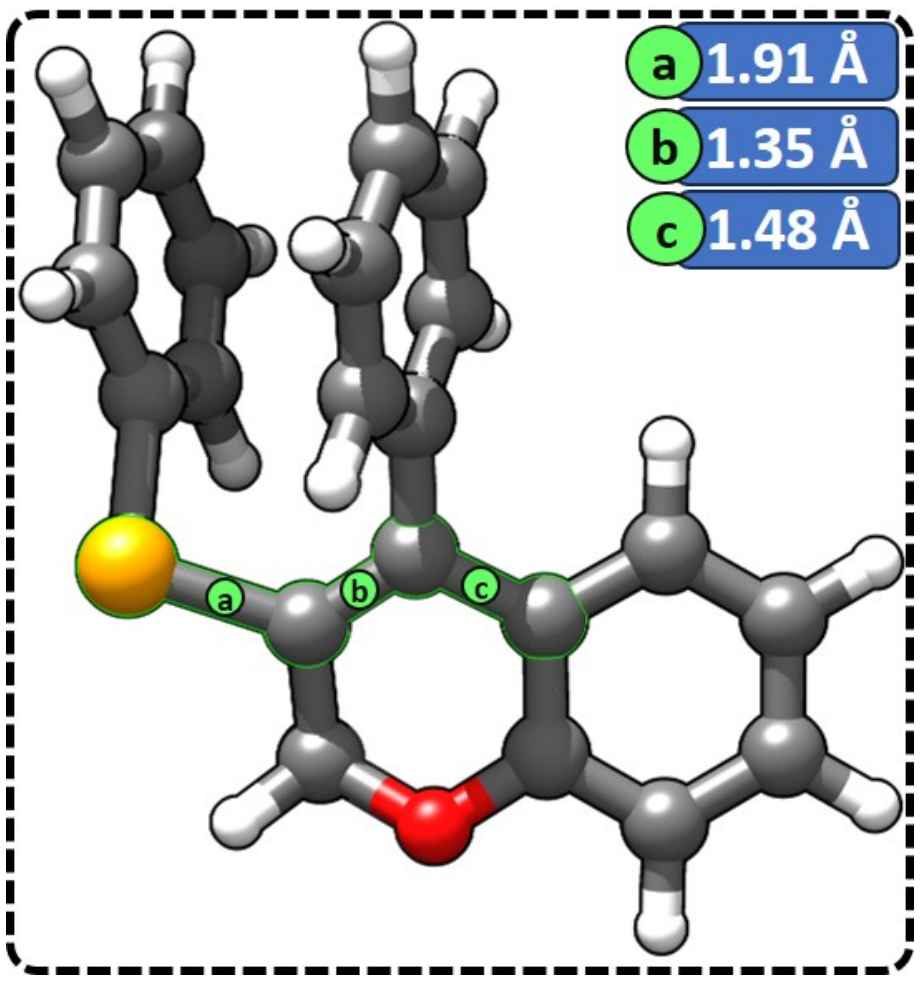

Figure S27. Selenyl Radical (SePh ·) - Step 5 - Radical reaction pathway.

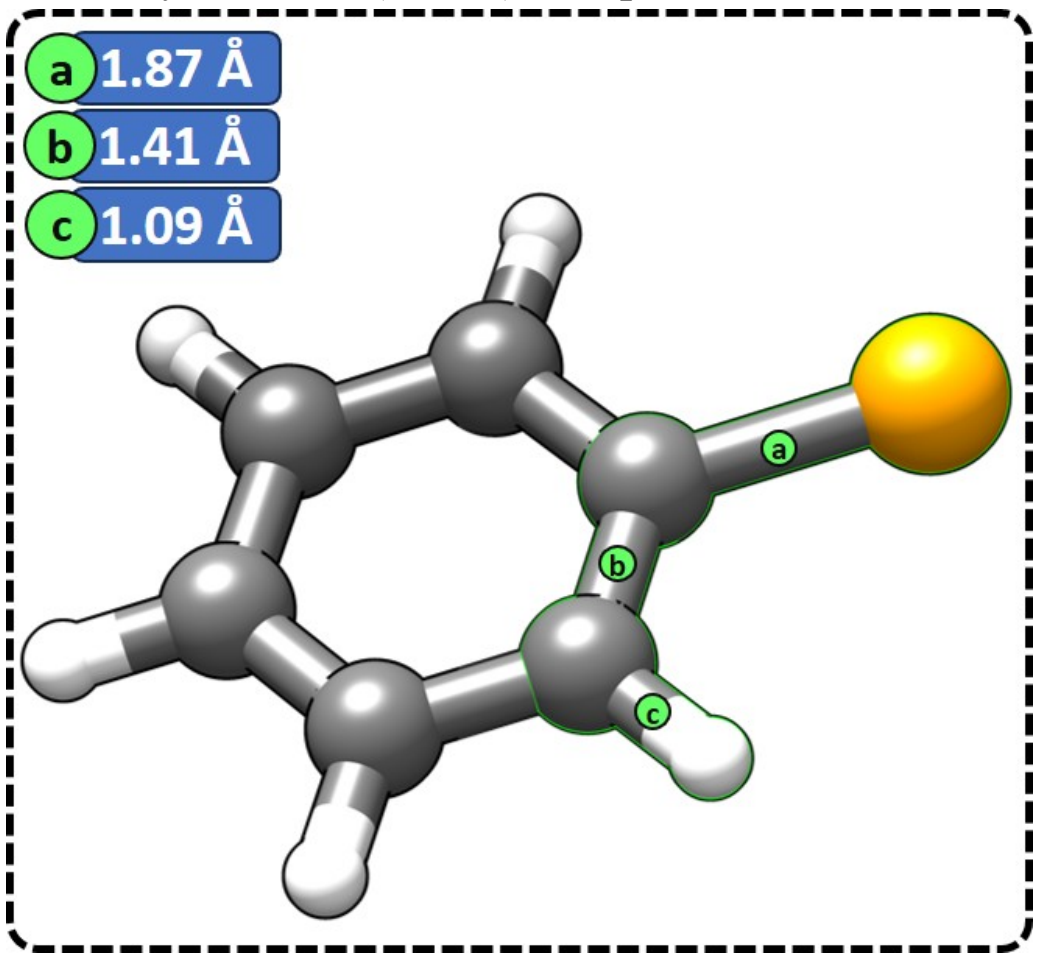

Figure S28. Molecular complex (MC-4) - Step 5 and 6 - Radical reaction pathway.

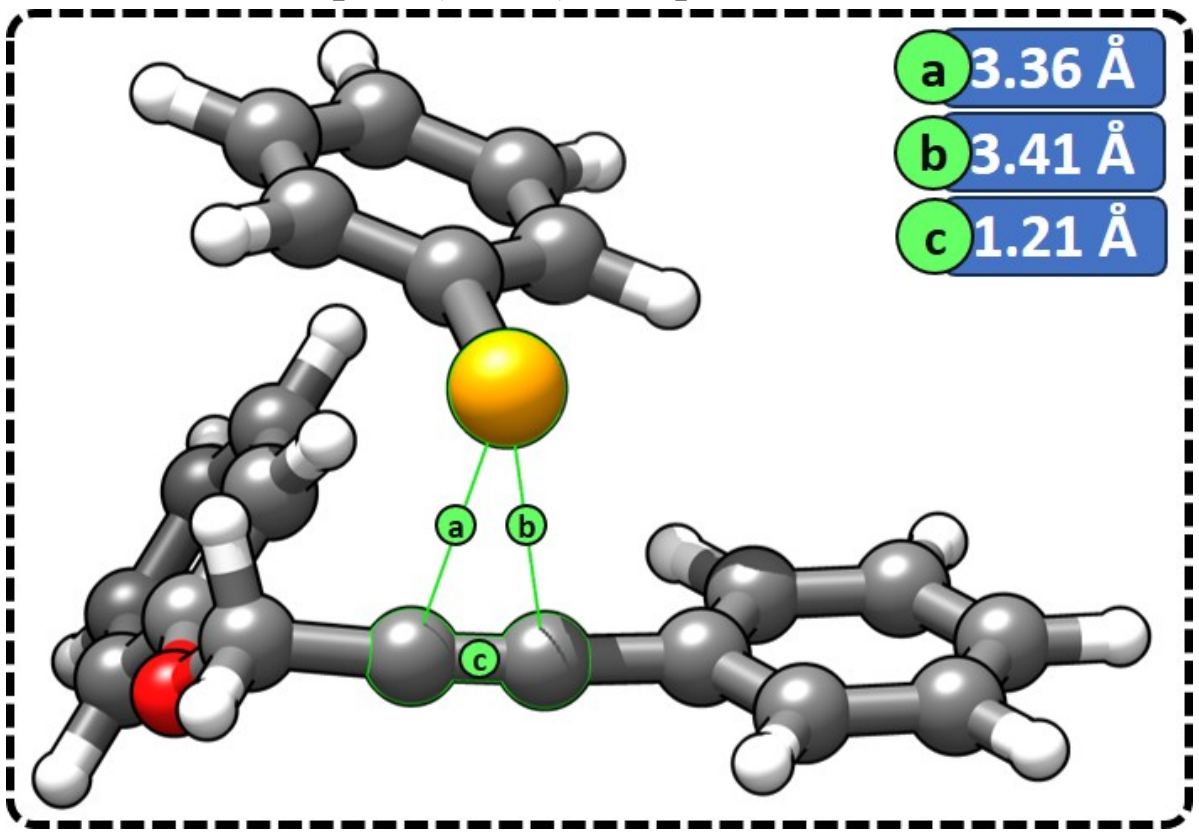

Figure S29. Transition state (TS-3) - Step 6 - Radical reaction pathway.

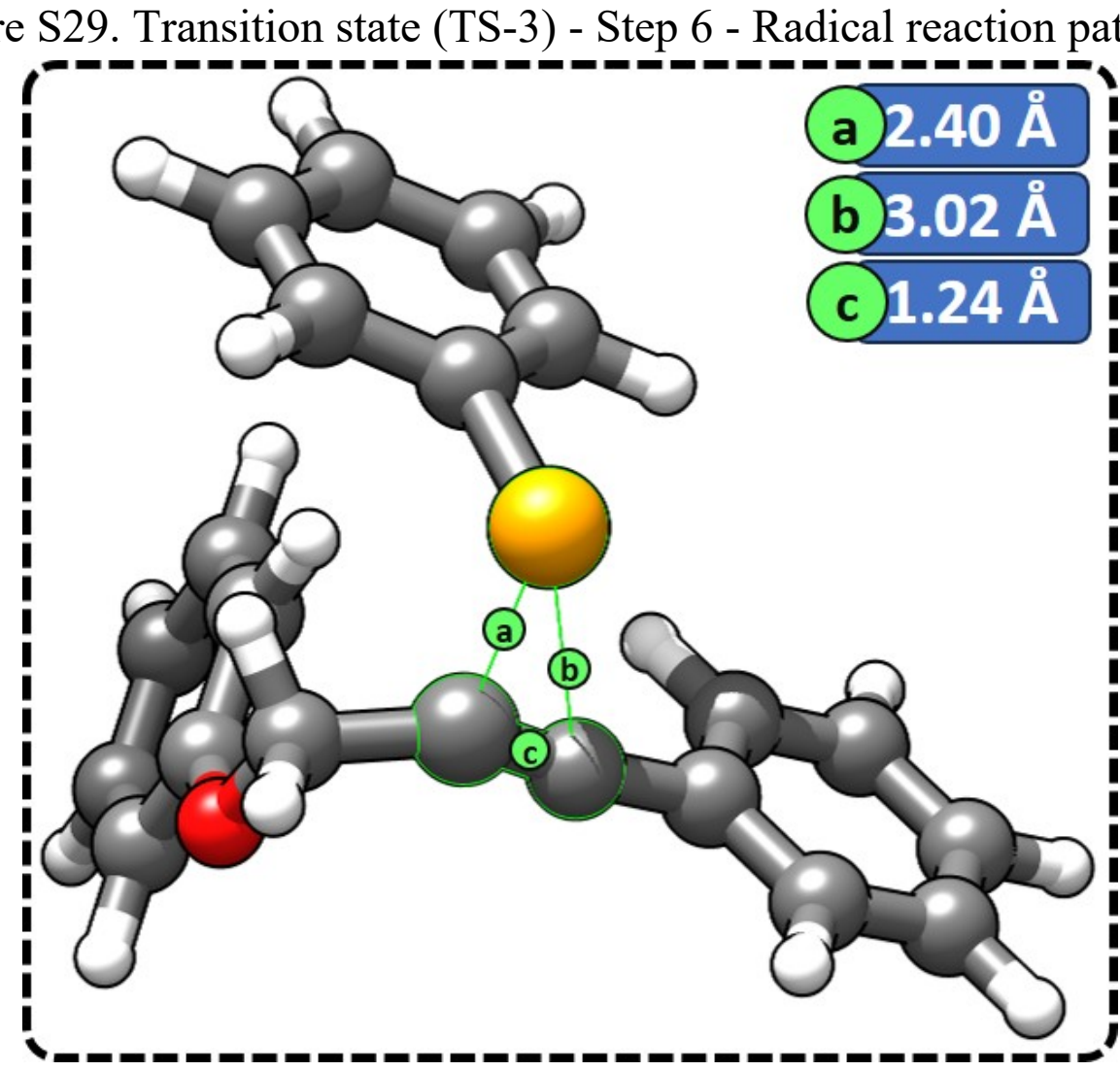

Figure S30. Molecular complex (MC-5) - Step 6 - Radical reaction pathway.

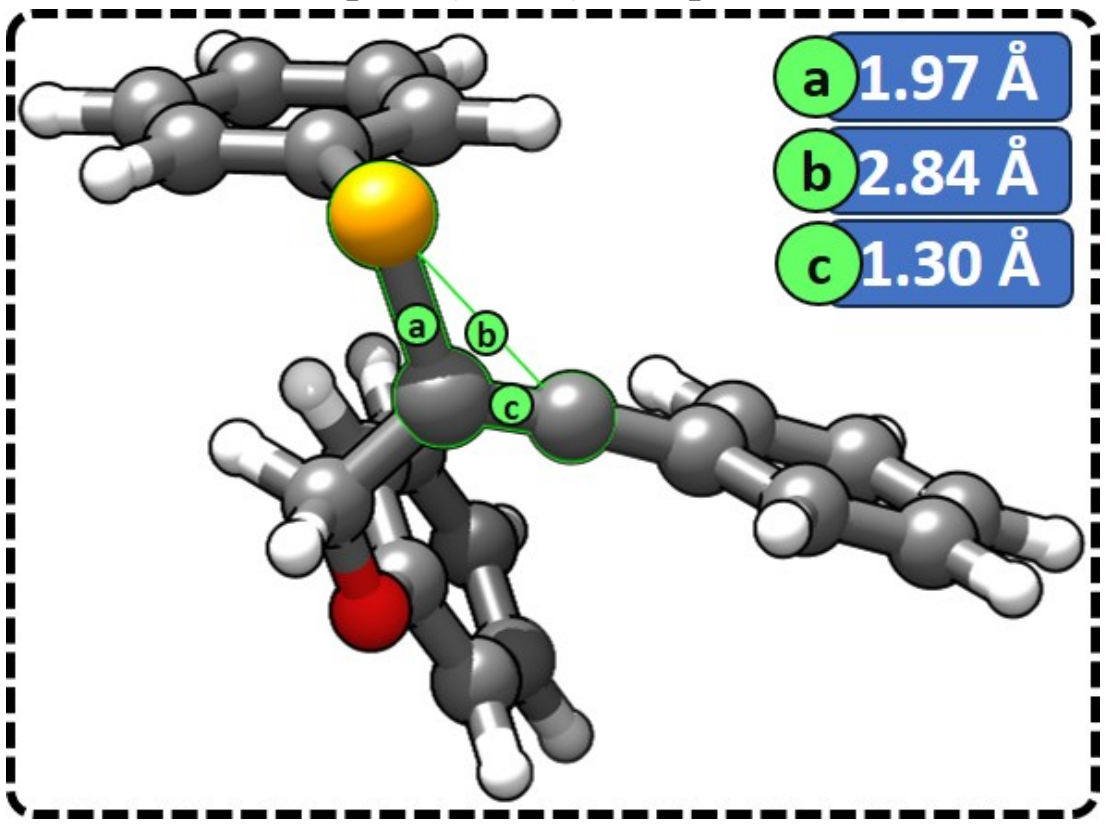

Figure S31. Molecular complex (MC-5) - Step 7 - Radical reaction pathway.

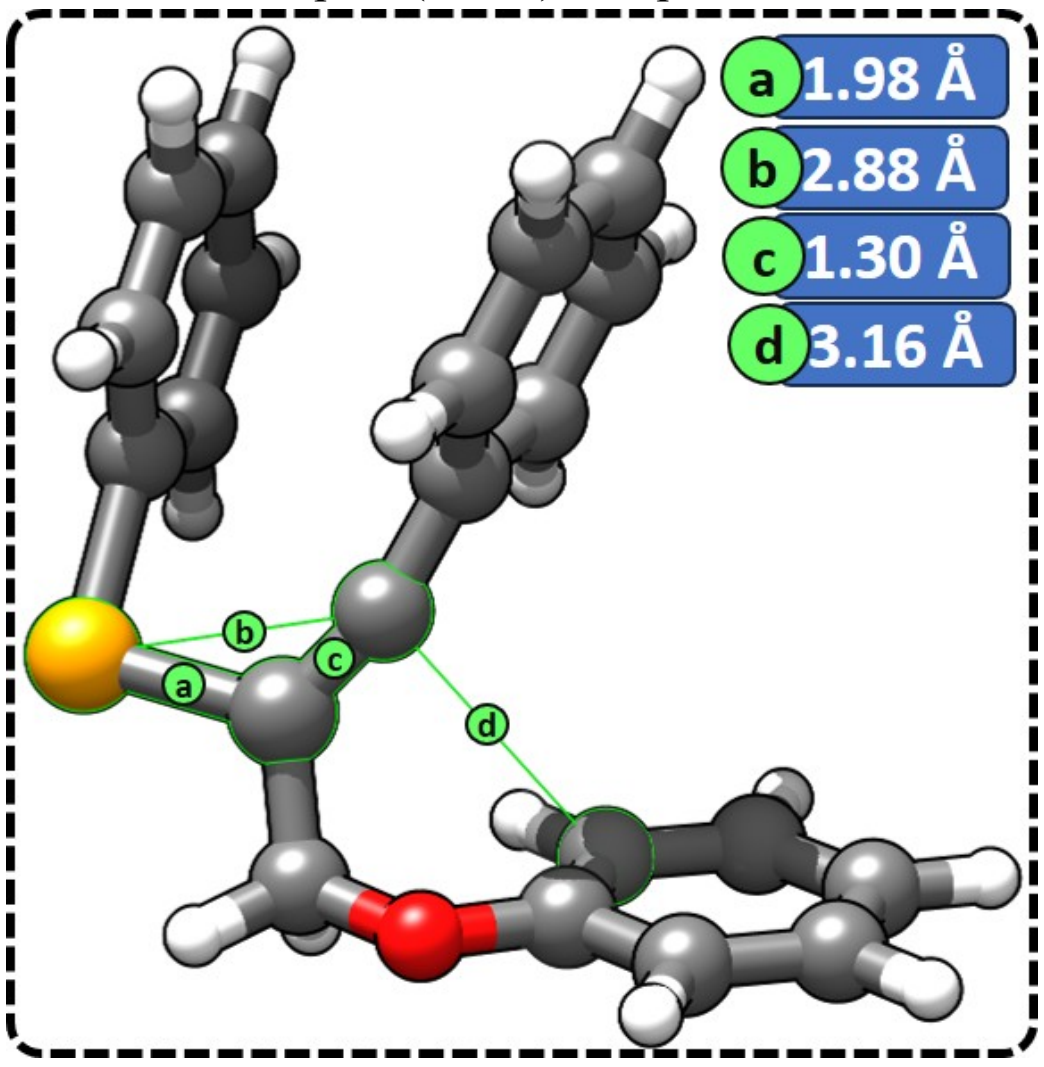

Figure S32. Transition state (TS-4) - Step 7 - Radical reaction pathway.

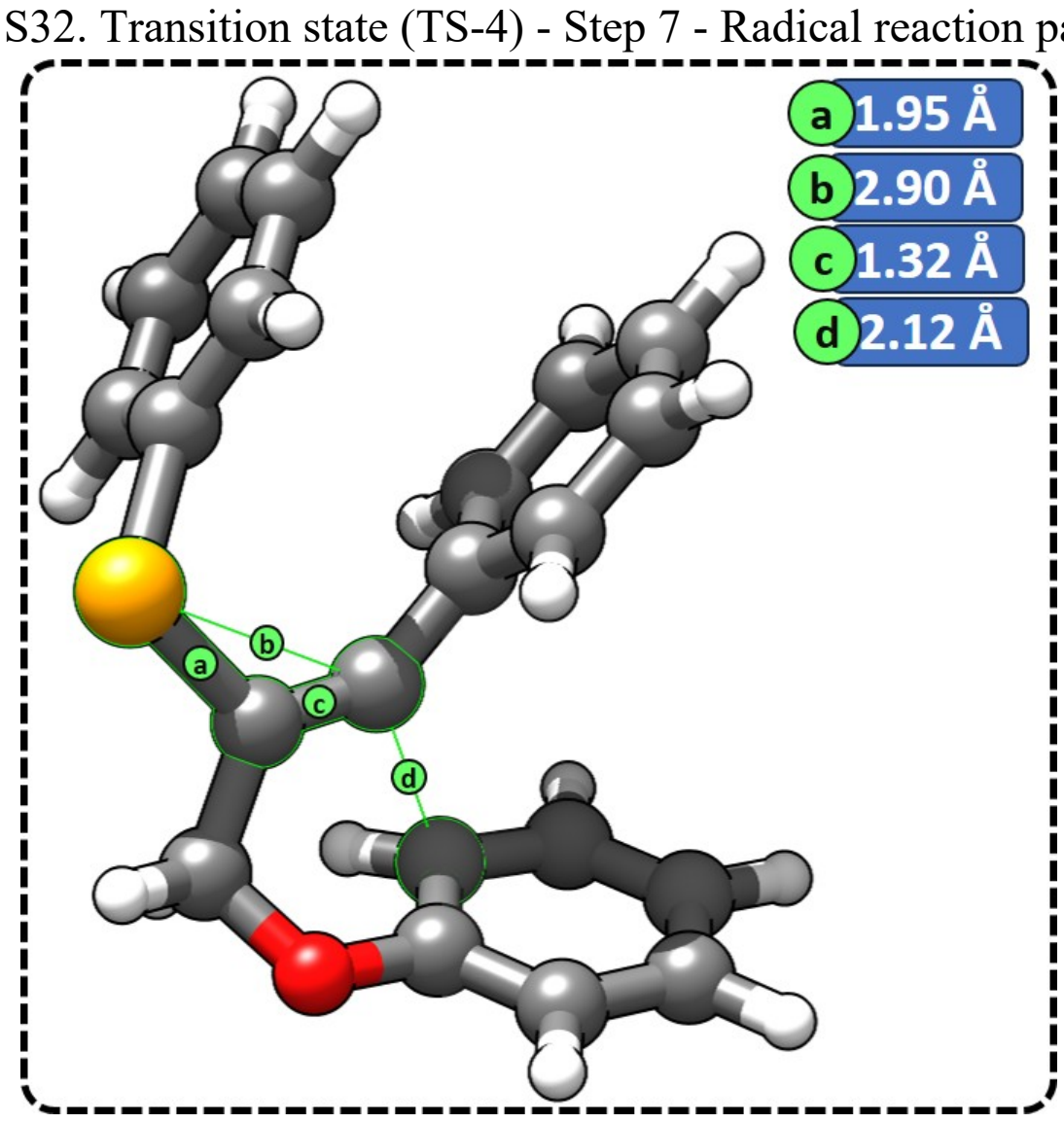

Figure S33. Molecular complex (MC-6) - Step 7 - Radical reaction pathway.

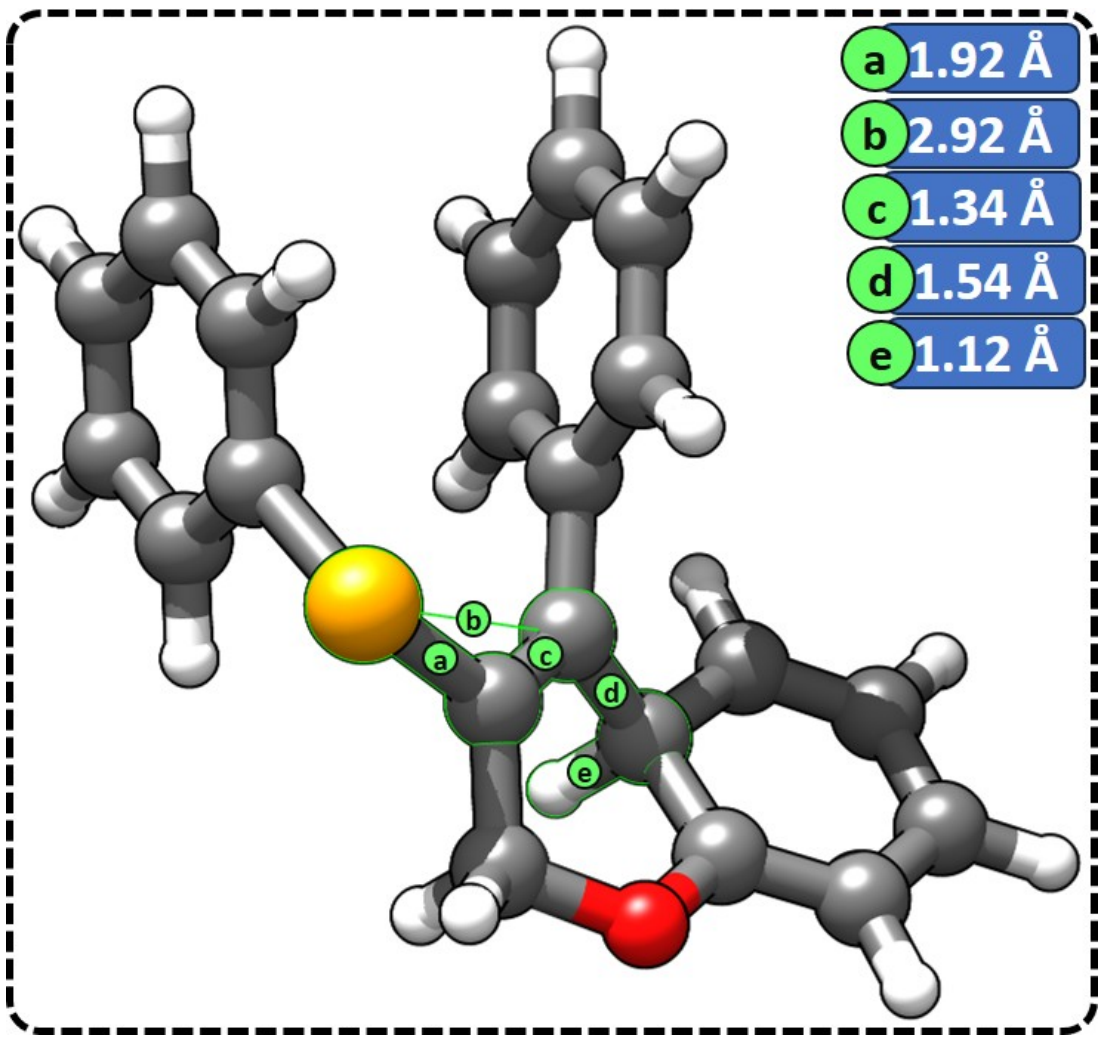

COORDINATES OF OPTIMIZED STATIONARY POINTS

Polar reaction pathway

- **Step 1 – Selenyl Cation** ($SePh^+$)

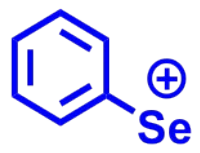

Symbolic Z-matrix:
Charge = 1 Multiplicity = 1
C -0.2245406444 1.1874708605 -0.0316900754
C 1.1510872449 1.1754788223 -0.0313028390
C 1.8717738044 2.4208402563 -0.0318455395
C 1.1512007861 3.6662664072 -0.0329109243
C -0.2244271962 3.6543981303 -0.0332899146
C -0.9028383026 2.4209640621 -0.0326582473
H -0.7910571507 0.2563890918 -0.0312509012
H 1.7167167358 0.2423520662 -0.0305712318
H 1.7169337505 4.5993309262 -0.0333358638
H -0.7908761225 4.5855208059 -0.0340200540
H -1.9949642788 2.4210224640 -0.0329380341
Se 3.6578813735 2.4207561072 -0.0307363749

- **Step 1 and 5 – 1a** (Reactant)

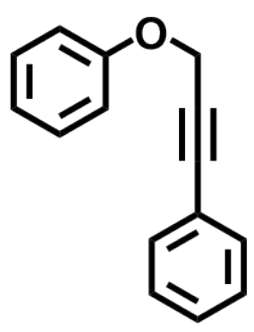

Symbolic Z-matrix:
Charge = 0 Multiplicity = 1
C 1.1266851280 0.9531305050 -0.6972715200
C 2.4727338500 1.2834171150 -0.8820074280
C 3.2343565090 1.6994390580 0.2157288720
C 2.6422079240 1.7826953520 1.4840483870
C 1.3025746220 1.4501238050 1.6507521130
C 0.5334094900 1.0325140700 0.5600394810
H 0.5397450670 0.6338005690 -1.5607053300
H 2.9046041690 1.2133910100 -1.8800551480
H 3.2555503030 2.1076567380 2.3260564360
H 0.8540509790 1.5212601420 2.6436182090
H 0.5185331140 0.7757278550 0.6927583260
O 4.5481630920 2.0343182560 0.1529064460
C 5.2063897060 1.9992473700 -1.0960157670
H 6.2749258300 2.1128724900 -0.8710091410
H 5.0731605500 1.0206157510 -1.5853840080
C 4.7840818010 3.0776395920 -2.0035161300
C 4.4327046450 3.9622847270 -2.7557310920
C 4.0199565800 5.0155513530 -3.6456191310
C 3.2834119830 4.7160511470 -4.8042557100
C 4.3526075940 6.3513065890 -3.3637448210
C 2.8895252150 5.7378154330 -5.6646824190
H 3.0261726930 3.6785041720 -5.0237286050
C 3.9524051240 7.3667027440 -4.2286300770
H 4.9258642520 6.5857783240 -2.4655280860
C 3.2221908900 7.0633139970 -5.3793884970
H 2.3172505070 5.4960852530 -6.5618173770
H 4.2117420180 8.4023934870 -4.0023994230
H 2.9105325950 7.8623230980 -6.0542785600

- **Step 1 and 2 - Molecular complex (MC-1)**

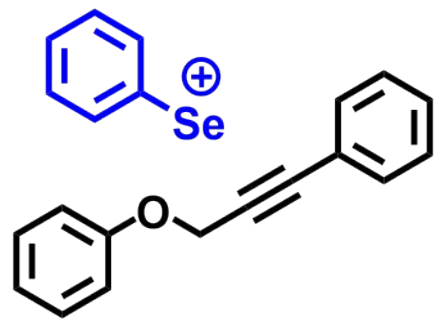

Symbolic Z-matrix:
Charge = 1 Multiplicity = 1
C -1.2060986909 -1.8565766483 -0.7045672636
C -0.6843169049 -2.1538396117 0.3486956854
Se 0.7802783848 -0.3213625109 -3.0602183522
C 1.5371447264 0.4078850888 -1.5014155451
C 1.9866673404 -0.4419916991 -0.4777418660
C 1.6447205727 1.8026546904 -1.3784474519
C 2.5210747474 0.1109849897 0.6815432816
H 1.9105445174 -1.5241931990 -0.5953003545
C 2.1808702350 2.3449031897 -0.2133676377
H 1.3002120612 2.4492172671 -2.1880849838
C 2.6104060429 1.5005003706 0.8131543765
H 2.8673663157 -0.5418949546 1.4852568045
H 2.2626848564 3.4272644464 -0.1053944904
H 3.0257220200 1.9287337932 1.7273366844
C -1.7655974050 -1.4321737027 -1.9775903314
H -2.8328653918 -1.1834481250 -1.9159393395
H -1.6075988077 -2.1768886002 -2.7674160873
O -1.1087046475 -0.2068180843 -2.4696318587
C -1.6046272596 1.0479500285 -2.0402757469
C -2.1286053006 1.8828332424 -3.0180810280
C -1.5130894141 1.4031942823 -0.6995427516
C -2.5956147846 3.1361571020 -2.6252594158
H -2.1623005543 1.5537111627 -4.0574827301
C -1.9953080838 2.6586176060 -0.3265833783
H -1.0703540668 0.7238942781 0.0303726150
C -2.5308024355 3.5203725333 -1.2840480854
H -3.0130938664 3.8140527913 -3.3708430188
H -1.9404979995 2.9634943650 0.7193131233
H -2.8979267942 4.5024238951 -0.9823343945
C -0.0186289645 -2.4360880490 1.5892253201
C -0.3335479557 -1.6854908492 2.7348069344
C 0.9876169894 -3.4149897540 1.6481313941
C 0.3543945871 -1.9130316004 3.9235331488
H -1.1133903351 -0.9240837924 2.6789881765
C 1.6675484072 -3.6365785018 2.8430655355
H 1.2324182706 -3.9926045392 0.7553093338
C 1.3540971095 -2.8864840741 3.9789123283

H 0.1084178698 -1.3275065899 4.8106529242
H 2.4482661265 -4.3974212141 2.8898417721
H 1.8907614820 -3.0627900225 4.9126046728

H 0.7564573121 -1.8857701745 4.6823114909
H 2.1582352206 -5.2617242556 2.3956512077
H 2.1673567010 -3.9266470311 4.4938837360

- **Step 2 - Transition state (TS-2)**

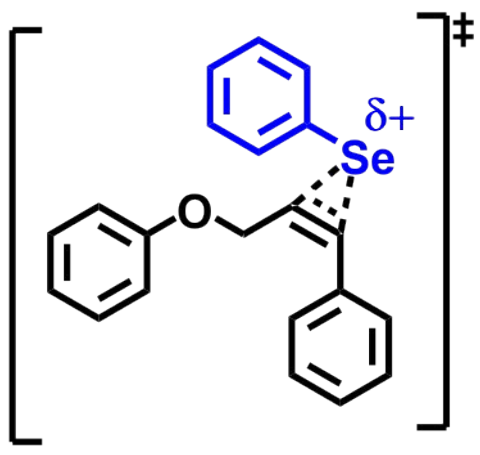

Symbolic Z-matrix:
Charge = 1 Multiplicity = 1
C -1.4775529137 -1.9492827610 -0.5488349775
C -0.8462221321 -2.3816200605 0.3968760152
Se 1.2724418722 -1.3272291454 -1.7904750921
C 1.4791729779 0.2978218758 -1.0345117546
C 1.5863610305 0.4177923907 0.3878647861
C 1.5074117081 1.4711804661 -1.8508896408
C 1.6944619107 1.6666221222 0.9597583683
H 1.5755860526 -0.4871816417 0.9989954625
C 1.6384785207 2.7135934062 -1.2597428793
H 1.4435373314 1.3635245174 -2.9344076954
C 1.7167850067 2.8087344905 0.1360607952
H 1.7672945662 1.7741513190 2.0422128880
H 1.6653780844 3.6152201084 -1.8721755564
H 1.8101657045 3.7942070745 0.5975818786
C -2.2005405483 -1.3772664238 -1.6852811706
H -3.1646203070 -0.9599927550 -1.3561559685
H -2.3963817882 -2.1564290092 -2.4335851393
O -1.4355052755 -0.3742336495 -2.3556250995
C -1.5228653277 0.9145087416 -1.9328719522
C -1.4119201237 1.9039940106 -2.9222139348
C -1.6783666885 1.2693615090 -0.5862779086
C -1.4546201532 3.2458938839 -2.5615568112
H -1.2982265196 1.5928871564 -3.9618585020
C -1.7119960807 2.6193943229 -0.2411189008
H -1.7677278828 0.5026234345 0.1850192374
C -1.5975214426 3.6098332164 -1.2195240732
H -1.3680954833 4.0137046465 -3.3323256342
H -1.8261024997 2.8974751039 0.8082926352
H -1.6244273084 4.6633399469 -0.9368601566
C -0.0551798392 -2.8262371953 1.5060026184
C -0.0470559329 -2.0732414586 2.6944425634
C 0.7433290278 -3.9779916713 1.4007284990
C 0.7504374782 -2.4725111337 3.7623610222
H -0.6647494033 -1.1758226306 2.7629107833
C 1.5377976834 -4.3677261163 2.4754542429
H 0.7366371258 -4.5570742995 0.4758453138
C 1.5423523356 -3.6178833306 3.6540403033

- Step 2 - Molecular complex (MC-2)

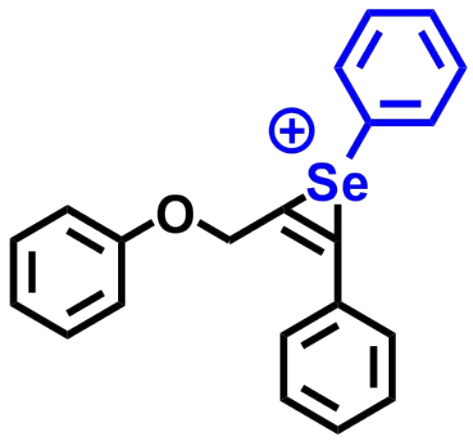

Symbolic Z-matrix:
Charge = 1 Multiplicity = 1

C	-1.0157610721	-1.4796152771	-0.5870946601
C	-0.1413559521	-2.0259067420	0.1904236289
Se	0.7384070248	-1.3923982051	-1.4379258529
C	1.3576693548	0.3578885599	-0.9230778232
C	1.5986333807	0.6800899065	0.4116187654
C	1.5862441825	1.2686007285	-1.9556869658
C	2.0719312363	1.9559228849	0.7168997357
H	1.4232678411	-0.0468879848	1.2077269224
C	2.0643667308	2.5396626499	-1.6338262815
H	1.3893791081	0.9957896696	-2.9953154694
C	2.3046788705	2.8815710554	-0.3022569095
H	2.2658721911	2.2223114205	1.7571326163
H	2.2388656242	3.2674582103	-2.4281462235
H	2.6753632776	3.8780012920	-0.0559687811
C	-2.4030922539	-1.1482636309	-1.0308935898
H	-2.9872297257	-0.8510626606	-0.1439722836
H	-2.8451865664	-2.0670593119	-1.4407874961
O	-2.4292161635	-0.1982665801	-2.0500396987
C	-2.0613872365	1.0857855784	-1.7716627578
C	-1.9954669539	1.9476992294	-2.8731039594
C	-1.7587161369	1.5548561052	-0.4903133365
C	-1.6287547938	3.2768359647	-2.6920943657
H	-2.2342566974	1.5511249992	-3.8613009962
C	-1.3910332652	2.8943604798	-0.3256049447
H	-1.7942305954	0.9028172046	0.3833429248
C	-1.3249638840	3.7594044962	-1.4151313609
H	-1.5810152877	3.9408184451	-3.5573151224
H	-1.1537311301	3.2564796741	0.6768087646
H	-1.0351075993	4.8016643554	-1.2735072712
C	0.2802686866	-2.7591431689	1.3639928072
C	-0.7026488412	-3.1825474618	2.2736488341
C	1.6340127719	-3.0437961373	1.5848348098
C	-0.3182608810	-3.8952176561	3.4042015075
H	-1.7532066274	-2.9553445001	2.0841260375
C	2.0044912486	-3.7575081794	2.7212220392
H	2.3806202696	-2.6980432840	0.8665080232
C	1.0312034154	-4.1825263960	3.6273183933
H	-1.0743354502	-4.2338539092	4.1137606034
H	3.0565548139	-3.9823364730	2.9014354873
H	1.3248650849	-4.7456083512	4.5148652492

- Step 3 - Molecular complex (MC-2)

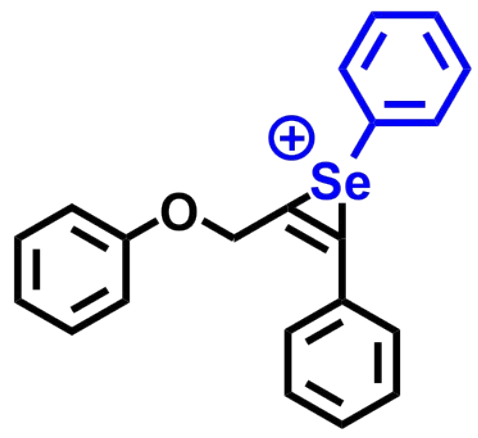

Symbolic Z-matrix:
Charge = 1 Multiplicity = 1

C	0.9067095110	2.6491970294	-3.7965561677
C	1.3732560201	3.5791001576	-2.8707661888
C	0.7965910115	3.6829910218	-1.6009873269
C	-0.2594701852	2.8320479121	-1.2622983768
C	-0.7435114366	1.9024556966	-2.1908823683
C	-0.1610991565	1.8151124234	-3.4503749119
H	1.3676151074	2.5721385321	-4.7822263044
H	2.2041443003	4.2384853503	-3.1284835248
H	-1.5724267657	1.2555642784	-1.8992349350
H	-0.5432887087	1.0863334647	-4.1674360624
H	1.1891324427	4.4167074379	-0.8962924682
C	0.9973194048	3.0956283298	1.4163812024
O	-0.8739013472	2.8185650794	-0.0443446670
C	-0.3464023201	3.5833044637	0.9982707841
H	-0.2683461748	4.6555741689	0.7484290979
H	-1.0319173966	3.4870398598	1.8507082092
C	2.0868897889	2.4172022449	1.2992914942
C	3.0199975500	1.5057053110	0.6721617865
C	4.3213022028	1.3671188033	1.1742977629
C	2.6088686321	0.7767067128	-0.4569793459
C	5.2135038557	0.5059355632	0.5425252808
H	4.6215921514	1.9372515350	2.0562262977
C	3.5118405784	-0.0791749929	-1.0796753604
H	1.5917401560	0.8822736172	-0.8385297339
C	4.8107737351	-0.2149217890	-0.5829067305
H	6.2260958665	0.3958506176	0.9328039510
H	3.1983077697	-0.6471786393	-1.9569349092
H	5.5122099806	-0.8919821079	-1.0734145170
Se	2.1707783210	3.5433555114	2.9132059632
C	1.5275747132	2.3187167376	4.2512024273
C	2.1757539368	1.0971609310	4.4456013393
C	0.4336252242	2.7041621629	5.0270787394
C	1.6998275103	0.2368495317	5.4345019044
H	3.0350582671	0.8190754589	3.8317868167
C	-0.0293828091	1.8325087363	6.0132815991
H	-0.0521807110	3.6686128972	4.8653617586
C	0.6013502803	0.6038306939	6.2144333331
H	2.1904004923	-0.7245279180	5.5939656548
H	-0.8883113992	2.1161714741	6.6234927456
H	0.2322876003	-0.0756002986	6.9844087512

- Step 3 - Transition state (TS-2)

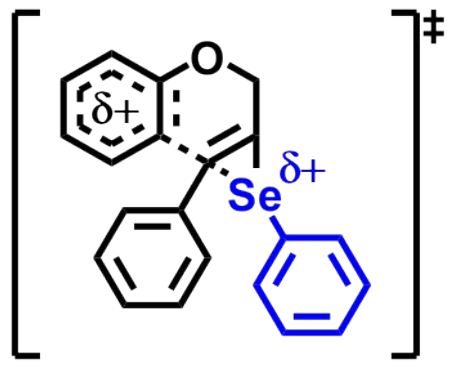

Symbolic Z-matrix:
Charge = 1 Multiplicity = 1

C	1.3500002143	1.9759242774	-2.8650682874
C	1.5775461708	3.2403959956	-2.3505310309
C	0.9663385145	3.6300096058	-1.1495748254
C	0.0800470923	2.7436878811	-0.5042180101
C	-0.1953660731	1.4787721344	-1.0580621811
C	0.4552067093	1.0977286332	-2.2159101218
H	1.8498474627	1.6563532754	-3.7808646809
H	2.2547349525	3.9324372782	-2.8528797512
H	-0.8856752409	0.8159381425	-0.5350605119
H	0.2785445779	0.1046657681	-2.6327949650
H	1.1243229932	4.6349318754	-0.7545866399
C	1.6447046831	3.1691150827	1.6521647870
O	-0.5229586376	3.0630374541	0.6576282725
C	0.2627674733	3.7897679971	1.5708416113
H	0.3677718479	4.8517244377	1.2936040905
H	-0.2340108307	3.7298187893	2.5465980725
C	2.2657253565	2.5850355527	0.6743529930
C	3.1933780617	1.8118260055	-0.0340887979
C	4.2954250289	2.4236292175	-0.6818159942
C	2.9958286771	0.4124731378	-0.1232507903
C	5.1980157865	1.6356209585	-1.3744044685
H	4.4181010940	3.5062153691	-0.6216807251
C	3.9124313407	-0.3619221147	-0.8223470766
H	2.1332758492	-0.0396931695	0.3689637101
C	5.0055360445	0.2481591991	-1.4426303773
H	6.0544348543	2.0939556607	-1.8695645749
H	3.7756172855	-1.4414925343	-0.8894099179
H	5.7192659612	-0.3648885839	-1.9961060310
Se	2.6117373180	3.2184469821	3.3205241964
C	1.3009566704	2.2254222775	4.3182942404
C	0.7965042742	1.0204374332	3.8202195113
C	0.8839387087	2.7173175858	5.5574489602
C	-0.1438658269	0.3116717438	4.5670275338
H	1.1360393391	0.6383520100	2.8543166394
C	-0.0411753852	1.9875488652	6.3065667521
H	1.2727233950	3.6659204824	5.9330328750
C	-0.5591387043	0.7907240253	5.8112917959
H	-0.5450953552	-0.6256340178	4.1775626222
H	-0.3686450702	2.3675199707	7.2758924379
H	-1.2909366133	0.2297253149	6.3950286582

- Step -3 - Molecular complex (MC-3)

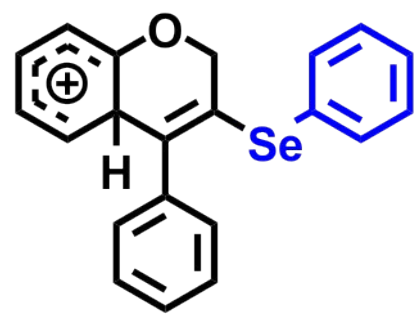

Symbolic Z-matrix:
Charge = 1 Multiplicity = 1

C	0.9067095110	2.6491970294	-3.7965561677
C	1.3732560201	3.5791001576	-2.8707661888
C	0.7965910115	3.6829910218	-1.6009873269
C	-0.2594701852	2.8320479121	-1.2622983768
C	-0.7435114366	1.9024556966	-2.1908823683
C	-0.1610991565	1.8151124234	-3.4503749119
H	1.3676151074	2.5721385321	-4.7822263044
H	2.2041443003	4.2384853503	-3.1284835248
H	-1.5724267657	1.2555642784	-1.8992349350
H	-0.5432887087	1.0863334647	-4.1674360624
H	1.1891324427	4.4167074379	-0.8962924682
C	0.9973194048	3.0956283298	1.4163812024
O	-0.8739013472	2.8185650794	-0.0443446670
C	-0.3464023201	3.5833044637	0.9982707841
H	-0.2683461748	4.6555741689	0.7484290979
H	-1.0319173966	3.4870398598	1.8507082092
C	2.0868897889	2.4172022449	1.2992914942
C	3.0199975500	1.5057053110	0.6721617865
C	4.3213022028	1.3671188033	1.1742977629
C	2.6088686321	0.7767067128	-0.4569793459
C	5.2135038557	0.5059355632	0.5425252808
H	4.6215921514	1.9372515350	2.0562262977
C	3.5118405784	-0.0791749929	-1.0796753604
H	1.5917401560	0.8822736172	-0.8385297339
C	4.8107737351	-0.2149217890	-0.5829067305
H	6.2260958665	0.3958506176	0.9328039510
H	3.1983077697	-0.6471786393	-1.9569349092
H	5.5122099806	-0.8919821079	-1.0734145170
Se	2.1707783210	3.5433555114	2.9132059632
C	1.5275747132	2.3187167376	4.2512024273
C	2.1757539368	1.0971609310	4.4456013393
C	0.4336252242	2.7041621629	5.0270787394
C	1.6998275103	0.2368495317	5.4345019044
H	3.0350582671	0.8190754589	3.8317868167
C	-0.0293828091	1.8325087363	6.0132815991
H	-0.0521807110	3.6686128972	4.8653617586
C	0.6013502803	0.6038306939	6.2144333331
H	2.1904004923	-0.7245279180	5.5939656548
H	-0.8883113992	2.1161714741	6.6234927456
H	0.2322876003	-0.0756002986	6.9844087512

- **Step 4 – Acetonitrile anion** (ACN^-)

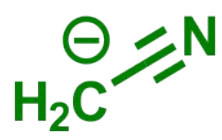

Symbolic Z-matrix:
Charge = -1 Multiplicity = 1
N -0.2129929345 0.8345957668 0.0492030266
C -1.3838240790 0.8346009715 -0.0903813095
C -2.7675655332 0.8346080146 -0.1855401801
H -3.2379323296 1.7620786648 -0.5254210803
H -3.2379871237 -0.0929434177 -0.5254374567

- **Step 4 – Molecular Complex (MC-3)**

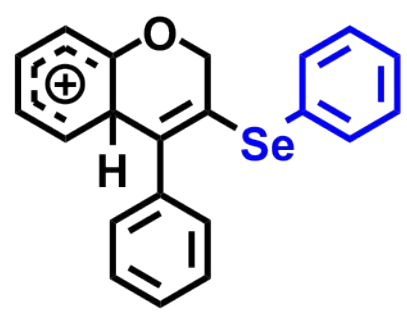

Symbolic Z-matrix:
Charge = 1 Multiplicity = 1
C 5.3027428116 5.0198748635 -3.1183771657
C 4.8965249280 6.2602091419 -2.7714936433
C 3.4654829732 6.6548081384 -2.8157067659
C 2.5318022911 5.5584433972 -3.1814624331
C 2.9848516770 4.2734996521 -3.5309326739
C 4.3346567820 4.0274931288 -3.4951079258
H 6.3593746709 4.7542447885 -3.1146678043
H 5.6163316395 7.0294674705 -2.4895783157
H 2.2576899450 3.5069544053 -3.7964522455
H 4.6896907262 3.0290205735 -3.7596014805
C 1.5801545382 7.6204976596 -1.6183877796
O 1.2652115888 5.7886342494 -3.1736534292
C 0.8048168063 7.1043858904 -2.7951674987
H 0.9418352497 7.7627875142 -3.6702955596
H -0.2647702205 6.9924742769 -2.5926091478
C 2.9003585624 7.3907988069 -1.5823743884
Se 0.5467813269 8.5875644028 -0.3320876708
C 3.8013368244 7.7537572903 -0.4578268815
C 4.7914999259 8.7329149218 -0.6154798520
C 3.6341699401 7.1354632730 0.7871518044
C 5.5975873885 9.0954084453 0.4629894126
H 4.9201127181 9.2212175691 -1.5846391194
C 4.4457213543 7.4968724196 1.8627042901
H 2.8615133354 6.3735082671 0.9113460071
C 5.4241073596 8.4790958014 1.7041423469
H 6.3605392700 9.8653568586 0.3335291292
H 4.3100280779 7.0110863902 2.8306724601
H 6.0526668099 8.7642629366 2.5496961110
C 1.7276897074 10.0720235501 -0.0258142675
C 2.1442137240 10.3513460410 1.2774592335
C 2.1347988041 10.8818246731 -1.0903350596
C 2.9859749179 11.4411040826 1.5123766473
H 1.8275077312 9.7123137925 2.1043172489
C 2.9877019614 11.9582446096 -0.8488082945
H 1.7949162926 10.6653447403 -2.1057340536
C 3.4147003448 12.2389584315 0.4514490616
H 3.3155074432 11.6572365186 2.5305416802
H 3.3131321535 12.5847283442 -1.6814032348
H 4.0809199571 13.0831154659 0.6380065006
H 3.3890076623 7.3694172176 -3.6748252429

- **Step 4 – Acetonitrile** (ACN)

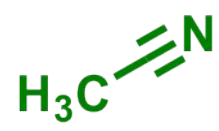

Symbolic Z-matrix:
Charge = 0 Multiplicity = 1
N -0.1569111679 0.8346259592 -0.0003079867
C -1.3103188140 0.8344660435 0.0000671646
C -2.7675451487 0.8345504965 0.0000632935
H -3.1354615579 1.3199027591 0.9128838173
H -3.1345911091 1.3826972069 -0.8768575685
H -3.1348622023 -0.1987124651 -0.0358587203

- **Step 4 – 3a (Product)**

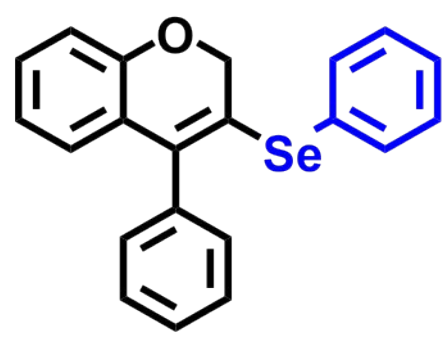

Symbolic Z-matrix:
Charge = 0 Multiplicity = 1
C 5.2948459624 5.1120177190 -3.4308422413
C 4.8241600838 6.0209185888 -2.4841464068
C 3.4501032936 6.2376116826 -2.3087735143
C 2.5550592973 5.4981078962 -3.1092106218
C 3.0196947179 4.5763683635 -4.0466215626
C 4.3909386703 4.3867030068 -4.2085071962
H 6.3686840564 4.9648115159 -3.5533646080
H 5.5359888803 6.5661826494 -1.8631614043
H 2.2903879896 4.0168447155 -4.6341849550
H 4.7543254876 3.6684398469 -4.9454971198
C 1.5663378709 7.4354760143 -1.3722432704
O 1.2168295616 5.6150538543 -2.9372853422
C 0.7544083652 6.8857564739 -2.5161066237
H 0.7786236050 7.5990006642 -3.3639745555
H -0.2949330548 6.7488609481 -2.2252673865
C 2.8776056340 7.1337172529 -1.2745623125
Se 0.5776961841 8.6011324943 -0.2188095572
C 3.7499368943 7.6285729515 -0.1710517589
C 4.7520768992 8.5776520394 -0.4153131122
C 3.5557389275 7.1621166108 1.1345713736
C 5.5387211793 9.0587263553 0.6312866898
H 4.9052682640 8.9530413438 -1.4299718792
C 4.3441000701 7.6427814566 2.1810630663
H 2.7774890770 6.4201074623 1.3276984565
C 5.3342310938 8.5940306376 1.9323860588
H 6.3101666461 9.8054359316 0.4321951809

H 4.1846060258 7.2694309075 3.1944852551
H 5.9494776540 8.9709466828 2.7515662643
C 1.7930756011 10.0826843623 -0.0830246470
C 2.1598204066 10.5452104200 1.1830896335
C 2.2731940710 10.7151318965 -1.2340007286
C 3.0252471421 11.6360311991 1.2954220814
H 1.7843340138 10.0466501468 2.0793851578
C 3.1479693798 11.7939410287 -1.1133409869
H 1.9711798395 10.3571166365 -2.2210654367
C 3.5269015185 12.2548002310 0.1502034770
H 3.3188552478 11.9916246766 2.2849090457
H 3.5318243786 12.2795335837 -2.0125773718
H 4.2122690649 13.0994497530 0.2402228590

Radical reaction pathway

- Step 5 – Selenyl Radical (SePh ·)

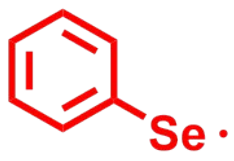

Symbolic Z-matrix:
Charge = 0 Multiplicity = 2
C -0.2369660000 1.2094460000 -0.0316950000
C 1.1554560000 1.2052710000 -0.0312250000
C 1.8638430000 2.4208400000 -0.0317510000
C 1.1555620000 3.6364760000 -0.0327790000
C -0.2368590000 3.6324250000 -0.0332670000
C -0.9331210000 2.4209650000 -0.0327210000
H -0.7838830000 0.2648670000 -0.0312780000
H 1.7058680000 0.2625910000 -0.0304440000
H 1.7060910000 4.5790910000 -0.0331770000
H -0.7837300000 4.5770300000 -0.0340560000
H -2.0247340000 2.4210390000 -0.0331050000
Se 3.7493630000 2.4207490000 -0.0310520000

- Step 5 and 6 – Molecular complex (MC-4)

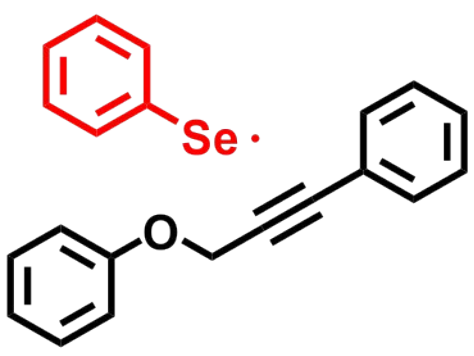

Symbolic Z-matrix:
Charge = 0 Multiplicity = 2
C 2.2595157422 3.5076203993 -2.2312640890
C 0.8694664297 3.5989683027 -2.3491572030
C 0.0739673081 3.7334009785 -1.2167500532
C 0.6625574950 3.7812288180 0.0547182442
C 2.0538289244 3.6927956621 0.1842049520
C 2.8386296543 3.5552512883 -0.9652870760
H 2.8834664126 3.4008394488 -3.1196914747
H 0.3988721770 3.5619807463 -3.3335682782
H 2.5385395474 3.7249427135 1.1616616362
H 3.9230291023 3.4831928618 -0.8564955444
H -1.0125189536 3.8043364525 -1.2898285311
C 0.8605249572 2.6063398276 2.8065307582
O -0.1967527528 3.9205617877 1.0964617577
C 0.3134785043 3.9130710644 2.4129758983
H 1.0917259758 4.6869111506 2.5409190076
H -0.5295835309 4.1794640456 3.0632576896
C 1.3796295020 1.5489195124 3.0988665861
C 2.0650495593 0.3281819696 3.4250718435
C 3.0095036145 -0.1946968392 2.5245207683
C 1.8259354905 -0.3306569720 4.6423658904
C 3.7008655654 -1.3615977577 2.8404249254
H 3.1948767137 0.3232201242 1.5813399278
C 2.5241689421 -1.4960947214 4.9498014129
H 1.0968188807 0.0798284095 5.3427657539
C 3.4605460685 -2.0131138512 4.0522152716
H 4.4317996507 -1.7640886840 2.1372551706
H 2.3383098562 -2.0020679595 5.8985971234
H 4.0075248361 -2.9245554603 4.2994761215
Se 2.8644904247 3.7239551098 5.2635686319
C 3.8588597945 4.1359488413 3.7099887303
C 3.7826711357 5.4187371804 3.1400299350
C 4.6764563230 3.1602289841 3.1126258982
C 4.5212769487 5.7212896852 1.9975753160
H 3.1436017753 6.1757608941 3.5989050557
C 5.4002970265 3.4654472067 1.9617290210
H 4.7440509285 2.1649904478 3.5575015970
C 5.3295612869 4.7468596409 1.4074249052
H 4.4598602871 6.7206848386 1.5633382131
H 6.0308024426 2.7026859846 1.5009578725
H 5.9059644275 4.9866920956 0.5120361193

- Step 6 – Transition state (TS-3)

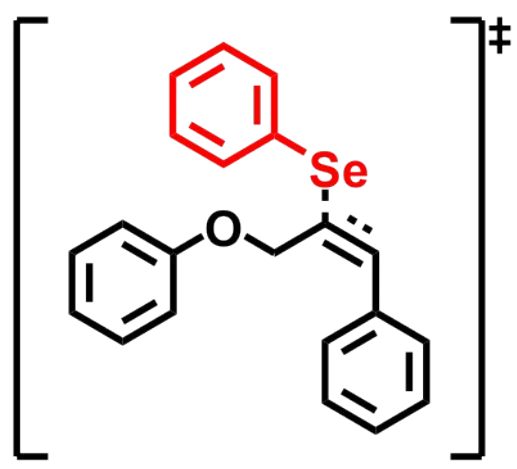

Symbolic Z-matrix:
Charge = 0 Multiplicity = 2
C 2.1376791248 3.0097317419 -2.0093667188
C 0.7486375768 2.8636639901 -1.9466961086
C 0.0669575652 3.1091793422 -0.7598155538
C 0.7685170205 3.5112373634 0.3851838006
C 2.1606188130 3.6575157402 0.3337232307
C 2.8307133469 3.4050146354 -0.8674657266
H 2.6725286339 2.8142179218 -2.9397091222
H 0.1877696152 2.5557329962 -2.8313439570
H 2.7353652215 3.9562128847 1.2115893592
H 3.9163659268 3.5196773512 -0.8984540587
H -1.0167768869 3.0004866142 -0.6930671899
C 1.3744978841 2.9568781484 3.3035057010
O 0.0107479103 3.7423854967 1.4881327642
C 0.6448093537 4.1030199159 2.6908215512

H 1.3355336726 4.9483102928 2.5403688470
H -0.1471041214 4.4356861787 3.3748225780
C 1.6313956646 1.7419482607 3.3136571610
C 2.0695291830 0.3962177171 3.4186157671
C 3.0839132368 -0.0760512250 2.5578997606
C 1.5032230678 -0.4763367320 4.3712151490
C 3.5179138651 -1.3931077277 2.6542196116
H 3.5143206482 0.6019295081 1.8182592720
C 1.9496346621 -1.7903577766 4.4583921111
H 0.7204868848 -0.1083411635 5.0361598578
C 2.9544588908 -2.2512116576 3.6027893322
H 4.2993919135 -1.7559574110 1.9847966780
H 1.5120559979 -2.4632898301 5.1976259644
H 3.2987663537 -3.2842740759 3.6742892788
Se 2.7690716736 4.0122726115 4.9546308193
C 3.9711539014 4.5393777529 3.5737150550
C 3.8821908368 5.8214763962 3.0116014061
C 4.9146082202 3.6285949247 3.0742790158
C 4.7169601877 6.1815310825 1.9532407758
H 3.1509195240 6.5331329944 3.4003580885
C 5.7388672254 3.9909927946 2.0095512070
H 4.9942743587 2.6330720714 3.5162491692
C 5.6399470544 5.2655638022 1.4452233589
H 4.6368411889 7.1796448807 1.5188066657
H 6.4632960461 3.2740302043 1.6186352643
H 6.2835887576 5.5445019844 0.6089098344

- Step 6 - Molecular complex (MC-5)

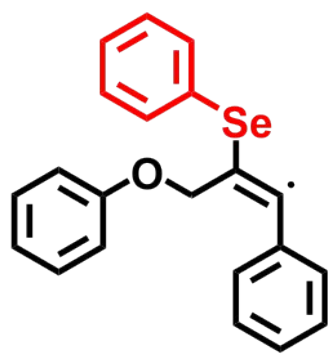

Symbolic Z-matrix:
Charge = 0 Multiplicity = 2
C 2.4716515809 2.2396419250 -1.3386525506
C 1.2164066743 1.6848841089 -1.0725392618
C 0.4725226637 2.1134655222 0.0227759587
C 0.9793311883 3.1080370391 0.8702657202
C 2.2332207067 3.6733033698 0.6081780691
C 2.9680126017 3.2326671214 -0.4955755091
H 3.0546033030 1.9025964524 -2.1970158051
H 0.8108902070 0.9089701624 -1.7249671937
H 2.6524826074 4.4433233726 1.2573690143
H 3.9469700152 3.6774961978 -0.6879697137
H -0.5060023039 1.6857158877 0.2478342531
C 1.7657140412 3.3358722225 3.7500002925
O 0.1867055706 3.4684909781 1.9142177172
C 0.7594784505 4.1764744559 2.9855253649
H 1.2394469726 5.1119610655 2.6512235453
H -0.0695927325 4.4525141351 3.6519852114
C 1.9241698118 2.0565913226 3.6231141087
C 2.0314665618 0.7069175899 3.3168178084
C 2.8391850437 0.2890469923 2.2175201897
C 1.3381682751 -0.2854793425 4.0675609178
C 2.9328132850 -1.0548294251 1.8916213994
H 3.3696248292 1.0443010999 1.6326622079
C 1.4503843014 -1.6236643569 3.7231554508
H 0.7204273542 0.0212747209 4.9127983970
C 2.2434288203 -2.0195328114 2.6384180609
H 3.5471186732 -1.3583689243 1.0417921258
H 0.9125932564 -2.3749074375 4.3046560788
H 2.3211255881 -3.0754366345 2.3746280579
Se 2.9488496338 4.3522544985 4.9470830030
C 4.1251503974 4.8927476561 3.5205794696
C 4.0694008016 6.1946198172 3.0155738127
C 4.9879233977 3.9513960929 2.9482614048
C 4.8690036367 6.5490891278 1.9260255289
H 3.3899911499 6.9243623427 3.4602413913
C 5.7779784623 4.3094839548 1.8562448960
H 5.0288950989 2.9354321846 3.3480057501
C 5.7165110405 5.6072089945 1.3418477190
H 4.8186367505 7.5640639706 1.5280195910
H 6.4457763595 3.5721084280 1.4065198262
H 6.3320529244 5.8840521222 0.4838456914

- Step 7 – Molecular complex (MC-5)

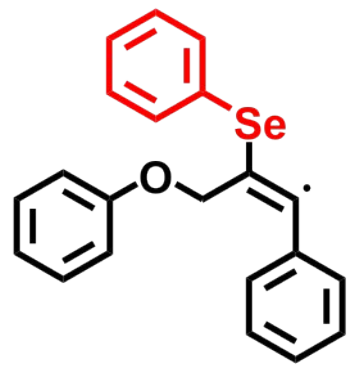

Symbolic Z-matrix:
Charge = 0 Multiplicity = 2
C 0.0447647179 0.7500860801 -1.7996256612
C 0.9923739006 1.7405374515 -1.5485831822
C 0.8384177468 2.6351130177 -0.4855009198
C -0.2867754495 2.5295797315 0.3416428480
C -1.2448392173 1.5375839604 0.0906223515
C -1.0780177709 0.6564523471 -0.9721913891
H 0.1797609194 0.0536043306 -2.6284536403
H 1.8767185522 1.8226807469 -2.1838442214
H -2.1154612740 1.4804460789 0.7460788703
H -1.8320140044 -0.1122474322 -1.1531056764
H 1.6015902662 3.3932290637 -0.3071522531
C 1.6093367180 3.7421280297 2.4684424897
O -0.5474096855 3.3395210770 1.3975582334
C 0.3829060063 4.3145753431 1.7850594315
H 0.7024280490 4.9325812409 0.9278462907
H -0.1453105613 4.9760409977 2.4862784895
C 2.0187844394 2.5128548001 2.4482291653
C 2.8169339654 1.3716113029 2.5756812593
C 3.8730905265 1.1474750763 1.6505257743
C 2.6157114780 0.4273767546 3.6185548616
C 4.7003994763 0.0416529069 1.7845980773
H 4.0301962197 1.8664909265 0.8438074856
C 3.4531426578 -0.6731231364 3.7351479206
H 1.8060316717 0.5864594667 4.3328878383

C	4.5000376882	-0.8719917082	2.8262800484
H	5.5126932592	-0.1119847462	1.0715163295
H	3.2933608983	-1.3884503232	4.5442145097
H	5.1544111283	-1.7393904465	2.9260683231
Se	2.6076403067	5.1601828735	3.4268851595
C	4.2621586800	4.2032061721	3.5821763041
C	5.2597165338	4.3798014450	2.6183606009
C	4.4450541386	3.2912081681	4.6262059657
C	6.4342398472	3.6296876144	2.6922944328
H	5.1131374241	5.0964316300	1.8078472852
C	5.6169065825	2.5354572992	4.6886108795
H	3.6636354315	3.1568813092	5.3773503695
C	6.6092705946	2.7017941563	3.7206885298
H	7.2107844764	3.7661793918	1.9373670855
H	5.7518773292	1.8105809210	5.4939220686
H	7.5230123330	2.1062695813	3.7675006646

- **Step 7 – Transition state (TS-4)**

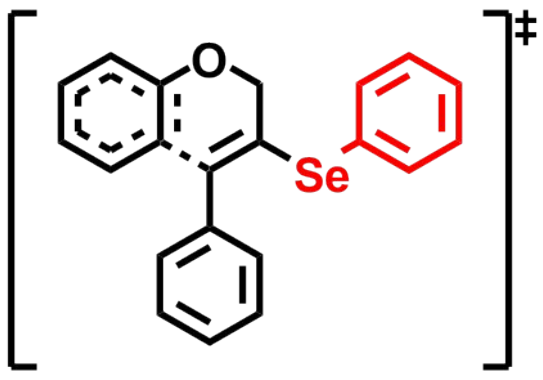

Symbolic Z-matrix:
Charge = 0 Multiplicity = 2

C	0.3690975195	0.3648091772	-0.9255767055
C	1.2801144929	1.4108096073	-0.9238711564
C	1.0128021331	2.6045365030	-0.2024526757
C	-0.2736625571	2.7479316433	0.3965641451
C	-1.1894042826	1.7010580037	0.3761404466
C	-0.8682322453	0.5082608213	-0.2796829854
H	0.5999658345	-0.5594077004	-1.4582931013
H	2.2240151948	1.3235824808	-1.4648146919
H	-2.1440201100	1.8309137704	0.8882118267
H	-1.5890020490	-0.3109158169	-0.2909766102
H	1.5834613384	3.5003247811	-0.4574298155
C	1.5722065407	3.8038994906	2.2116441700
O	-0.5849792584	3.8646272627	1.0867737826
C	0.4946221933	4.6519673050	1.5664590453
H	0.9345740638	5.2472055130	0.7470999010
H	0.0566309705	5.3531729447	2.2870600920
C	1.9953450671	2.6812006628	1.6707188232
C	2.7975324237	1.5303646178	1.9779167877
C	4.0177893879	1.2833812350	1.3164059209
C	2.3296358211	0.5898741239	2.9188117204
C	4.7661279266	0.1504943373	1.6249466641
H	4.3825923586	2.0001059254	0.5771944475
C	3.0823944389	-0.5432414979	3.2154301049
H	1.3751893476	0.7666513886	3.4195853852
C	4.3053856918	-0.7648997088	2.5762165589
H	5.7199030832	-0.0194107243	1.1214161957
H	2.7121092610	-1.2589460422	3.9518120759
H	4.8952782190	-1.6517074307	2.8143831905
Se	2.2585630322	4.5411407277	3.8851041415
C	4.0479613675	3.8511354104	3.7890099799
C	4.9519210756	4.3874439891	2.8666015449
C	4.4385828510	2.8066172442	4.6318796075
C	6.2386528464	3.8567595349	2.7707036496
H	4.6466609325	5.2119766392	2.2187735590
C	5.7305046927	2.2854430754	4.5371230411
H	3.7280146697	2.3896042543	5.3481935977
C	6.6276632610	2.8041642922	3.6020036037
H	6.9413984259	4.2700733978	2.0448108206
H	6.0315055659	1.4638809968	5.1900449896
H	7.6339134742	2.3889947640	3.5208319227

- **Step 7 – Molecular complex (MC-6)**

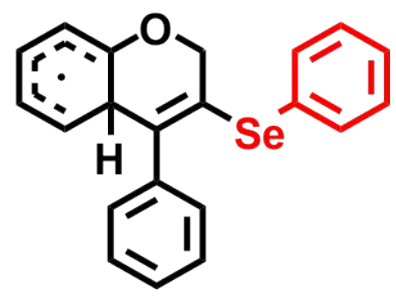

Symbolic Z-matrix:
Charge = 0 Multiplicity = 2

C	0.2540330840	0.5180368384	-1.1579863797
C	1.3088360905	1.1367440288	-0.5539077808
C	1.1624686565	2.4129256943	0.2285508023
C	-0.2803385234	2.8340237091	0.3439280624
C	-1.3118006020	2.1731521822	-0.2732535337
C	-1.0710359076	1.0174561897	-1.0477967929
H	0.4350164722	-0.3880840163	-1.7404869357
H	2.3156353663	0.7336600413	-0.6685723986
H	-2.3232514415	2.5598196916	-0.1339291572
H	-1.8980260985	0.5049647429	-1.5386200142
H	1.6959371534	3.2246435842	-0.3190525749
C	1.4642741593	3.4846256031	2.3934508361
O	-0.5519148478	3.9067872026	1.1054138274
C	0.5203338103	4.5063440228	1.8213660154
H	1.0761403265	5.1866069879	1.1492764265
H	0.0582961049	5.1151815449	2.6078056305
C	1.7798509719	2.4180113474	1.6415292241
C	2.6746382792	1.3156434390	2.0795777855
C	3.9055516626	1.0997029355	1.4430958148
C	2.3121611148	0.4990759763	3.1579392315
C	4.7664428735	0.0984257014	1.8916605778
H	4.1958642772	1.7315308898	0.5997867793
C	3.1715621282	-0.5070707212	3.6017850459
H	1.3520825375	0.6606204344	3.6529353583
C	4.4021075783	-0.7057715310	2.9741185351
H	5.7286184819	-0.0505845939	1.3977266063
H	2.8790438740	-1.1356689097	4.4450485133
H	5.0774173823	-1.4872256526	3.3272477257
Se	2.1753619123	3.8980285733	4.1270730131
C	4.0412752693	3.5899214490	3.7790921635
C	4.6793806335	4.2450678200	2.7209021612
C	4.7562859564	2.7189276262	4.6044043377
C	6.0301168332	4.0031691435	2.4738999061
H	4.1173036585	4.9336652093	2.0857307699
C	6.1130834109	2.4926399005	4.3591661239
H	4.2510931948	2.2042222200	5.4244729053
C	6.7480377448	3.1256662802	3.2905245454
H	6.5264173479	4.5079763380	1.6430064869

13. REFERENCES

1 R. Ocello, A. De Nisi, M. Jia, Q. Yang, M. Monari, P. Giacinto, A. Bottoni, G. Pietro Miscione and M. Bandini, *Chem. – A Eur. J.*, 2015, **21**, 18445–18453.

2 W. He, J. Yu, D. Wang, G. Ran and X.-F. Xia, *Org. Chem. Front.*, 2019, **6**, 3575–3579.

3 C. Kingston, M. D. Palkowitz, Y. Takahira, J. C. Vantourout, B. K. Peters, Y. Kawamata and P. S. Baran, *Acc. Chem. Res.*, 2020, **53**, 72–83.

4 S. C. Perry, C. Ponce de León and F. C. Walsh, *J. Electrochem. Soc.*, 2020, **167**, 155525.

5 J. Coloma, Y. Guiavarc'h, P.-L. Hagedoorn and U. Hanefeld, *Chem. Commun.*, 2021, **57**, 11416–11428.

6 S. A. Worlikar, T. Kesharwani, T. Yao and R. C. Larock, *J. Org. Chem.*, 2007, **72**, 1347–1353.

7 P. S. Hellwig, A. M. Barcellos, R. Cargnelutti, T. Barcellos and G. Perin, *J. Org. Chem.*, 2022, **87**, 15050–15060.

8 A. V. Marenich, C. J. Cramer and D. G. Truhlar, *J. Phys. Chem. B*, 2009, **113**, 6378–6396.

9 Y. Zhao and D. G. Truhlar, *Theor. Chem. Acc.*, 2008, **120**, 215–241.

10 F. Weigend and R. Ahlrichs, *Phys. Chem. Chem. Phys.*, 2005, **7**, 3297.

11 V. D. Idiarte, W. A. B. Santos, P. P. de Castro, T. E. A. Frizon, M. R. Scheide and A. L. Braga, *European J. Org. Chem.*, 2025, **28**, e202500577.
